# Influence of the Substitution Pattern of Terpene‐Based Seven‐Membered Lactones on Yttrium‐Mediated Ring‐Opening Polymerization: A Kinetic and Mechanistic Investigation

**DOI:** 10.1002/chem.202501380

**Published:** 2025-06-16

**Authors:** Lea‐Sophie Hornberger, Svenja Hiotidis, Shailja Jain, Michael Benz, Hugo Montan, Deven P. Estes, Johannes Kästner, Friederike Adams

**Affiliations:** ^1^ Chair of Macromolecular Materials and Fiber Chemistry, Institute of Polymer Chemistry University of Stuttgart Pfaffenwaldring 55 70569 Stuttgart Germany; ^2^ Institute of Theoretical Chemistry University of Stuttgart Pfaffenwaldring 55 70569 Stuttgart Germany; ^3^ Institute of Technical Chemistry University of Stuttgart Pfaffenwaldring 55 70569 Stuttgart Germany; ^4^ Section for Translational Research in Ophthalmology, Center for Ophthalmology University Eye Hospital Tübingen Elfriede‐Aulhorn‐Strasse 7 72076 Tübingen Germany; ^5^ Chair of Macromolecular Chemistry, TUM School of Natural Sciences, Department of Chemistry Technical University of Munich Lichtenbergstr. 4 85748 Garching Germany

**Keywords:** DFT calculations, mechanistic studies, polyester, ring‐opening polymerization, terpenes

## Abstract

Terpenes exhibit a wide range of structures, thus enabling diverse applications. Herein, (‐)‐menthide and *trans* (+)‐carvomenthide were synthesized from terpenoids (‐)‐menthone and (+)‐dihydrocarvone, differing solely in their substitution pattern. (‐)‐Menthide features a methyl group at position 4 and an isopropyl group at position 7, whereas (+)‐carvomenthide shows the inverse arrangement. Both lactones were transformed into polyesters via ring‐opening polymerization (ROP) using an amino‐alkoxy‐bis(phenolate) yttrium catalyst. Polymerization kinetics revealed first‐order behavior, with (+)‐carvomenthide polymerizing significantly faster than (‐)‐menthide, while (‐)‐menthide demonstrated a more controlled polymerization. Activation energies were 36.3 kJ mol^−1^ for (+)‐carvomenthide and 40.8 kJ mol^−1^ for (‐)‐menthide. The Gibbs free energy of activation confirmed the lower energy barrier for (+)‐carvomenthide polymerization, with experimental values of 81.9 kJ mol^−1^ compared to 89.1 kJ mol^−1^ for (‐)‐menthide. Density functional theory (DFT) calculations supported the experimental results, with computed Gibbs free energy barriers of 84.5 kJ mol^−1^ for *trans* (+)‐carvomenthide and 82.3 kJ mol^−1^ for (‐)‐menthide and mechanistic differences. Refined free energy barriers were determined to be 102.1 kJ mol^⁻1^ for (‐)‐menthide and 84.9 kJ mol^⁻1^ for *trans* (+)‐carvomenthide, agreeing with the experimental trend. These findings highlight the critical role of molecular structure and substituent position on polymerization kinetics and energy barriers in metal‐catalyzed polymerization.

## Graphical abstract

(‐)‐Menthide and *trans* (+)‐carvomenthide, differing only in substitution pattern, were polymerized via ROP using a bis(phenolate) yttrium catalyst. Kinetic studies showed faster polymerization of (+)‐carvomenthide but greater control with (‐)‐menthide. Experimental and DFT analyses revealed mechanistic differences in chain propagation, highlighting how molecular structure influences reactivity and polymerization control.

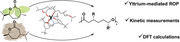

## 1 Introduction

Due to limited fossil resources, the demand for renewable and bio‐based chemicals is constantly increasing. Terpenes represent a large group of natural products and offer diverse structures and functionalities, making them ideal candidates for sustainable chemistry.^[^ ^1^ ^]^ Originating from isoprene, terpenes consist of C_5_ molecules, with monoterpenes featuring a C_10_ carbon skeleton formed from two isoprene groups. Terpenes are commonly found in essential oils, such as mint or caraway.^[^ ^2^ ^]^ (‐)‐Limonene, synthesized via enzyme‐catalyzed reactions in spearmint and peppermint, has garnered significant attention. Its biosynthesis involves a cyclization of geranyl pyrophosphate, which is catalyzed by limonene synthase enzyme.^[^ ^3^ ^]^ (‐)‐Limonene can be converted into (‐)‐menthone via hydroxylation to (‐)‐*trans*‐isopiperitenol and subsequent steps, such as dehydrogenation, hydrogenation, isomerization, and hydrogenation.^[^ ^4^ ^]^ (‐)‐Carvone is synthesized enzymatically from (‐)‐limonene via hydroxylation to (‐)‐*trans*‐carveol and subsequent dehydrogenation.^[^ ^5^ ^]^

In the field of environmentally friendly polymers, aliphatic polyesters are characterized by their degradability and biocompatibility. The synthesis of polyesters via ring‐opening polymerization (ROP) of lactones offers advantages over polycondensation, minimizing the formation of byproducts.^[^ ^6^ ^]^ Unlike step‐growth polycondensation, which requires precise stoichiometric balance and often leads to broad molecular weight distributions, chain‐growth polymerization such as ROP enables controlled polymerization with well‐defined molecular weights and compositions.^[^ ^6^ ^]^ The coordination‐insertion mechanism, in particular, ensures high polymerization efficiency and allows fine‐tuning of polymer properties.^[^ ^7^ ^]^ In 1958, Hall and Schneider were the first to explore the ROP of (‐)‐menthide, the corresponding lactone of (‐)‐menthone obtained by Baeyer‐Villiger oxidation, using sodium.^[^ ^8^ ^]^ Subsequent research by Hillmyer and Tolman utilized (‐)‐menthide in a zinc‐alkoxide‐mediated ROP.^[^ ^9^ ^]^ The phenoxyamino‐zinc catalyst enabled a polymerization process under mild conditions, resulting in poly((‐)‐menthide) with a high molecular mass of 90 kg mol^−1^. However, due to transesterification reactions, the high molecular weight polymers had high polydispersity indices.^[^ ^9^ ^]^ Lanthanide‐based catalysts that are stabilized by a *tert*‐butyl‐substituted amino‐methoxy‐bis(phenolate) ligand (ONOO)*^t^* ^Bu^ ((ONOO)*^t^* ^Bu^ = 6,6′‐(((2‐methoxyethyl)azane‐diyl)‐bis(methylene))‐bis(2,4‐di‐*tert*‐butylphenolate) show high activity and stereocontrol in the polymerization of small rings like *β*‐butyrolactone,^[^ ^8^, ^10^ ^]^ medium‐sized lactones,^[^ ^8^, ^10^, ^11^ ^]^ as well as macrolactones.^[^ ^10a^ ^]^ In a first study on terpene‐based lactones, Rieger et al. used amino alkoxy bis(phenolate) catalysts [(ONOO)*^t^* ^Bu^Y(bdsa)(THF)] (bdsa = bis(dimethylsilyl)amide, THF = tetrahydrofuran) to polymerize (‐)‐menthide.^[^ ^8^, ^11^ ^]^ This catalyst enabled polymerization under mild conditions, resulting in poly((‐)‐menthide) with a narrow molar mass distribution (*Ð* = 1.06–1.12) without showing substantial transesterification reactions.^[^ ^8^ ^]^ A summary of active catalytic systems for (‐)‐menthide and (+)‐carvomenthide polymerization is given in Table S1. Poly‐(‐)‐menthide is an amorphous polymer with a glass transition temperature of ‐25 °C, making it well‐suited as a soft segment in block copolymers.^[^ ^12^ ^]^ When copolymerized with other monomers such as *β*‐butyrolactone,^[^ ^8^ ^]^ lactide,^[^ ^12^, ^13^ ^]^ or acrylates,^[^ ^14^ ^]^ poly‐(‐)‐menthide has been shown to significantly reduce the Young's modulus and increase the elongation at break, thereby improving the elasticity of the resulting materials.^[^ ^12^, ^15^ ^]^ In combination with lactide, Young's moduli ranging from 1.4 to 25 MPa, and elongations of break of up to 960% have been reported, depending on the poly‐(‐)‐menthide content and polymer architecture.^[^ ^12^, ^13^ ^]^ Due to its slight miscibility with other polymer blocks, poly((‐)‐menthide) can also reduce the brittleness of adjacent glassy segments, enhancing mechanical performance in thermoplastic elastomer applications.^[^ ^12^, ^15^ ^]^ Another seven‐membered lactone with the same substituents is (+)‐carvomenthide. (+)‐Carvomenthide is derived from (+)‐carvone, which can be hydrogenated to (+)‐dihydrocarvone.^[^ ^16^ ^]^ Such hydrogenation reactions either involve the use of Wilkinson's catalyst^[^ ^17^ ^]^ or palladium on carbon^[^ ^18^ ^]^ to obtain (+)‐carvomenthone. (+)‐Carvomenthide can then be produced by oxidizing (+)‐carvomenthone.^[^ ^16c‐e^ ^]^ Herein, we describe another route to (+)‐carvomenthone by hydrogenation of (+)‐dihydrocarvide. To obtain (+)‐dihydrocarvide, (+)‐dihydrocarvone was oxidized via Baeyer‐Villiger oxidation using environmentally friendly Oxone.^[^ ^16c^ ^]^ Hillmyer and Tolman polymerized (+)‐carvomenthide using a diethylzinc (ZnEt_2_) catalyst at 100 °C, similar to the polymerization of (‐)‐menthide.^[^ ^16c,e^ ^]^ In another study, Kim and Shin employed tin(II) 2‐ethylhexanoate (Sn(Oct)_2_) in combination with diethylene glycol (DEG) as a bifunctional initiator at 100 °C, achieving 94% conversion with low polydispersity (1.05) after 22 hours.^[^ ^16d^ ^]^ Poly((+)‐carvomenthide) which exhibits material properties similar to poly‐(‐)‐menthide, is completely amorphous with a glass transition temperature around ‐27 °C.^[^ ^16c^ ^]^ Crosslinked poly((+)‐carvomenthide) could find application in thermoset elastomers, as demonstrated by Shin et al. They obtained high‐viscosity polymer networks with favorable mechanical properties, such as a storage modulus up to 340 kPa and a Young's modulus up to 1.13 MPa, both controllable through the degree of cross‐linking. The tensile strength was approximately 1.14 MPa for all samples and independent of the crosslinking density. All thermoset elastomers displayed excellent recovery behavior with a maximum strain of 50%.^[^ ^16d^ ^]^ Triblock copolymers of (+)‐carvomenthide and lactide have been investigated for thermoset elastomers showing superior and tunable mechanical strength. Storage moduli ranged between 2 and 168 MPa, Young's moduli between 2 and 77 MPa, and tensile strengths between 8 and 16 MPa. The strain at break exceeded 860% and reached up to 1100%.^[^ ^19^ ^]^ Further research on these terpene‐based seven‐membered lactones remains limited.

In this study, an in‐depth investigation of the kinetics of ROP of differently substituted terpene‐based seven‐membered lactones is presented. While both lactones, (‐)‐menthide and (+)‐carvomenthide, share the same molecular weights and compositions, they differ in the arrangement of their substituents. The polymerization kinetics of both monomers using [(ONOO)*^t^* ^Bu^Y(*i*PrO)(THF)] as a catalyst are studied, and the energies required for the respective polymerizations are compared. Utilizing the Arrhenius plot enables the calculation of activation energy, while the Eyring‐Polanyi equation, rooted in transition state theory, facilitates the determination of enthalpy and entropy of activation. The results are used for computing the Gibbs free energy of activation, representing the energy barrier between monomer and activated transition states. Furthermore, to elucidate the underlying reaction mechanisms, density functional theory (DFT) calculations are employed, focusing on the ROP of seven‐membered terpene‐based lactones with the [(ONOO)*^t^* ^Bu^Y(*i*PrO)(THF)] complex.

## 2 Results and Discussion

**Scheme 1:**
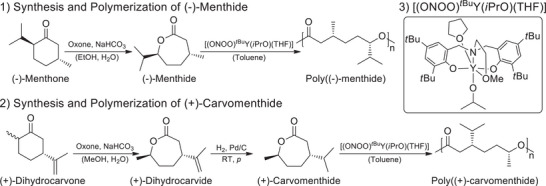
1) Synthesis of (‐)‐menthide from (‐)‐menthone via Baeyer‐Villiger oxidation and subsequent ROP to obtain poly((‐)‐menthide). 2) Synthesis of (+)‐carvomenthide from (+)‐dihydrocarvone through Baeyer‐Villiger oxidation and subsequent hydrogenation, followed by ROP to yield poly((+)‐carvomenthide). 3) Structure of the [(ONOO)*^t^* ^Bu^Y(*i*PrO)(THF)] complex used as a catalyst in yttrium‐mediated ROP.

Both monomers, (‐)‐menthide and (+)‐carvomenthide, were synthesized from their respective terpenoids. The synthesis of (‐)‐menthide was achieved through Baeyer‐Villiger oxidation of (‐)‐menthone, using Oxone as the oxidizing agent (Scheme 1). The conversion to the lactone was verified by ^1^H NMR spectroscopy, which revealed a downfield shift of the proton adjacent to the newly inserted oxygen atom due to its electron‐withdrawing effect (Figure S2). Gas chromatography‐mass spectrometry (GC‐MS) analysis confirmed the absence of impurities in the synthesized lactone (Figures S7, S8). (+)‐Carvomenthide was synthesized via a two‐step process starting from (+)‐dihydrocarvone (Scheme 1). In the first step, (+)‐dihydrocarvone, purchased as a mixture of *cis* and *trans* isomers, underwent Baeyer‐Villiger oxidation under conditions analogous to those used for (‐)‐menthone, yielding (+)‐dihydrocarvide. The oxidation involved the insertion of an oxygen atom into the C6‐ring of (+)‐dihydrocarvone, which was confirmed by ^1^H NMR spectroscopy (Figure S9). The doublet signal of the double bond in (+)‐dihydrocarvide remained visible, indicating successful oxidation without side reactions of the double bond. The isomers were separated via column chromatography. Consistent with literature reports, the *trans* isomer of (+)‐dihydrocarvide, referred to as DHC, eluted first and was primarily collected for further processing (Figures S10, S11).^[^ ^16c^ ^]^ The second step involved the hydrogenation of DHC to form (+)‐carvomenthide. The hydrogenation was performed at room temperature in an autoclave under high pressure of hydrogen, with palladium on carbon as the catalyst. ^1^H NMR spectroscopy confirmed full conversion by the disappearance of the ‐C = C*H*_2_ proton signal of DHC (Figure S12). GC‐MS analysis of (+)‐carvomenthide confirmed a purity of 96% (Figure S17). The substance generating the second elution peak in the GC‐MS showed the same molecular weight, suggesting that the remaining 4% impurity corresponds to the *cis* isomer, which is consistent with a small signal in the ^1^H‐NMR spectrum at 1.0 ppm, which could be assigned to the methyl groups assigned as *h* and *g* of the *cis* isomer (Figure S12). Ring‐opening polymerizations (ROPs) of (‐)‐menthide and (+)‐carvomenthide using [(ONOO)*^t^* ^Bu^Y(*i*PrO)(THF)] yielded poly((‐)‐menthide) and poly((+)‐carvomenthide), respectively. During polymerization experiments, reaction kinetics were monitored through time‐dependent sampling and analysis, enabling the determination of monomer conversion over time and the generation of linear first‐order kinetic plots to evaluate reaction rates. All components were weighed under an inert atmosphere in a glovebox and subsequently transferred to a heating block for polymerization. Aliquots were periodically removed from the reaction mixture for analysis, allowing for the determination of monomer conversion and molar masses and polydispersities (*Ɖ*) of the resulting polymers. The catalyst, [(ONOO)*^t^* ^Bu^Y(*i*PrO)(THF)], was prepared in situ by stirring the precursor complex, [(ONOO)*^t^* ^Bu^Y(bdsa)(THF)], itself already active in ROP, with one equivalent of isopropanol prior to polymerization. Polymerizations of (‐)‐menthide were conducted both in the presence and absence of isopropanol to investigate the effect of different initiators (Table S1; Figures S32‐S39). A targeted degree of polymerization (DP) of 25 was used, and reaction temperatures of 80 and 100 °C were selected. Turnover frequencies (TOF) were determined from the slope of conversion versus reaction time, and initiator efficiency was calculated as the ratio of theoretical to experimental molecular weight (Table S1). The initiator choice had little impact on monomer conversion, which remained high in all reactions (96–99%). Both initiator systems enabled controlled polymerizations, as indicated by relatively low dispersity values (*Ð* ≤ 1.24). However, polymerizations conducted with isopropanol exhibited slightly lower dispersities compared to those without isopropanol. The normalized turnover frequency TOF* (normalized TOF for the active metal centers) values for polymerizations with isopropanol were higher than those without, indicating a faster reaction. Ideally, the TOF* values for the same catalytic center should be identical, as the active metal centers are the same and the initiator has no influence on the propagation step. However, the observed discrepancy indicates that using bdsa as an initiator increases side reactions, leading to variations in TOF* values. Possible side reactions include induction periods, transesterification, and chain transfer, which can alter the number of active chains and affect polymerization kinetics. Due to the improved control observed with isopropanol as a nucleophile, [(ONOO)*^t^* ^Bu^Y(*i*PrO)(THF)] was selected as the preferred catalyst for in‐depth ROP studies of (‐)‐menthide and (+)‐carvomenthide.

To understand the chain initiation process, oligomerization experiments were performed with (‐)‐menthide and the in situ generated [(ONOO)*^t^* ^Bu^Y(*i*PrO)(THF)] catalyst in toluene. The resulting oligomerization mixture was analyzed via atmospheric pressure chemical ionization‐mass spectrometry (APCI‐MS) and showed signals for *m/z* = [*n* x *M*_M _+ *M_i_*_PrOH_ + H]^+^ with *n* = 2–7, confirming chain initiation by the isopropoxide unit (Figure S21). The same results were found in the APCI‐MS spectrum of oligomerization experiments of (+)‐carvomenthide (Figure S24), indicating the same initiation process for both monomers independent of the substitution pattern.

**Figure 1:**
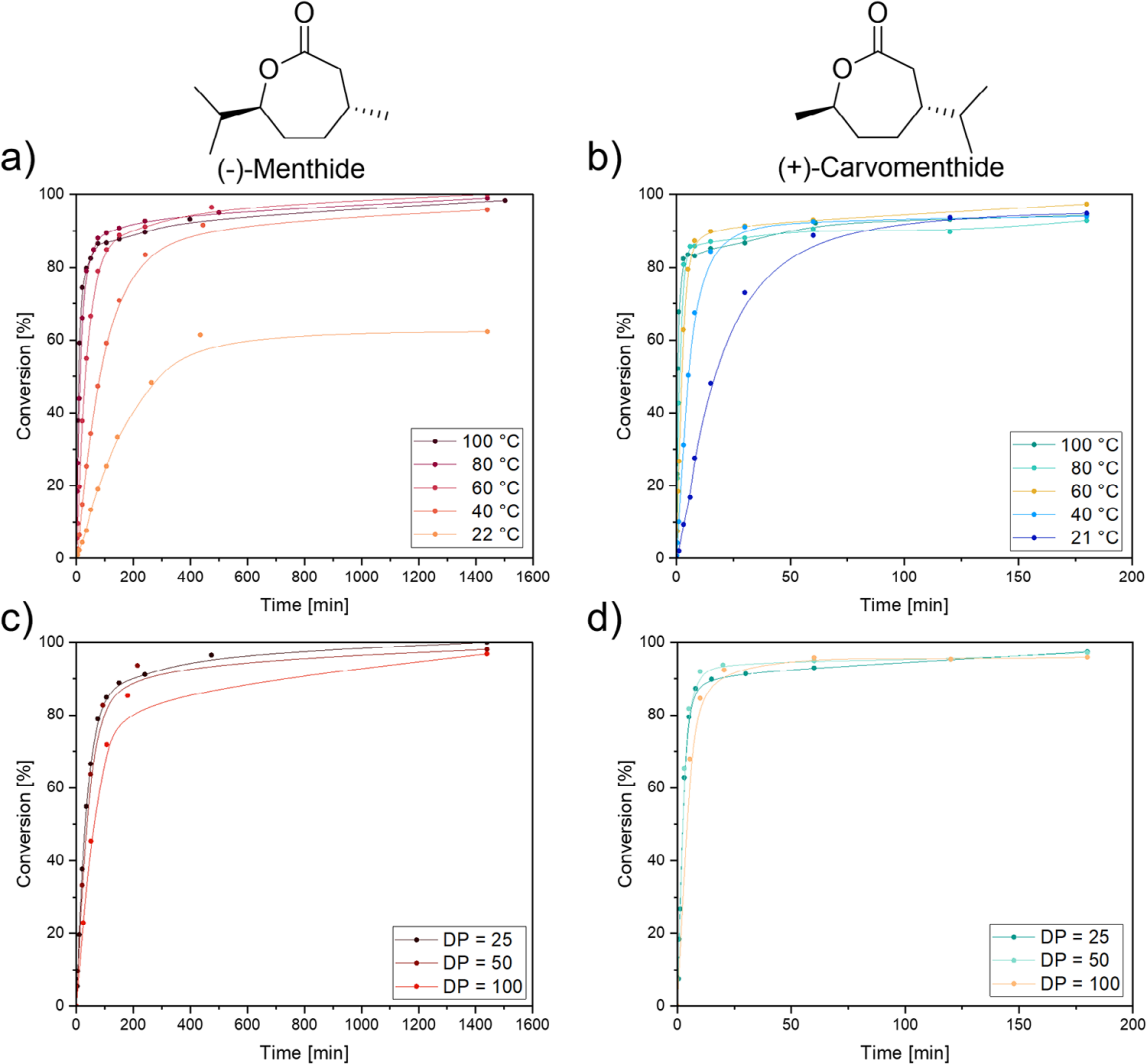
Top) Determination of monomer conversion over time through time‐dependent sampling and analysis at different temperatures. All reactions were performed with 25 equivalents of monomer and one equivalent of [(ONOO)*^t^* ^Bu^Y(*i*PrO)(THF)] in toluene for a) (‐)‐menthide and b) (+)‐carvomenthide; Bottom) Determination of monomer conversion over time through time‐dependent sampling and analysis using different targeted DPs. All reactions were performed at 60 °C and with one equivalent of [(ONOO)*^t^* ^Bu^Y(*i*PrO)(THF)] in toluene for c) (‐)‐menthide and d) (+)‐carvomenthide.

Further polymerizations were conducted solely with the in situ generated [(ONOO)*^t^* ^Bu^Y(*i*PrO)(THF)] catalyst in toluene under varying temperatures ranging from room temperature to 100 °C and different monomer‐to‐catalyst ratios between 25 and 100. First polymerizations were conducted using 25 equivalents of (‐)‐menthide (Table 1, Entries 1–3 and 6–7; Figures S25–S37). The conversion rates over time for all temperatures are depicted in Figure 1a. At 40 to 100 °C reaction temperature, polymerizations reached high monomer conversion and yielded high‐molecular‐weight polymers with low dispersities (*Ð* < 1.22; Table 1, Entries 1–3 and 6). In general, the polydispersity increased with rising temperature, likely due to side reactions such as transesterification or catalyst deactivation. Evidence of transesterification was observed as a deviation from the expected linear trend in the molecular weight versus conversion plot at higher monomer conversions. Specifically, polymerizations at 80 and 100 °C exhibited higher molecular weights than predicted at conversions exceeding 85%, suggesting that side reactions increase at elevated temperatures and are likely temperature‐dependent (Figures S35, S37). At room temperature, (‐)‐menthide polymerization exhibited a significantly reduced conversion of 62% (Table 1, Entry 7), resulting in lower molecular weight polymers. This suggests the presence of an energy barrier that limits reaction efficiency under these conditions. Although polydispersity increases with conversion, it remains constant with a value under 1.08 after reaching high conversions, indicating insignificant side reactions. Among these, polymerizations conducted at 40 and 60 °C provided the best results, achieving high conversions with minimal side reactions. The calculated TOF* values for (‐)‐menthide polymerization decreased with decreasing temperature, reflecting the influence of reduced thermal energy input on the reaction kinetics and monomer conversion per hour. At lower temperatures, the system lacks sufficient energy to efficiently surpass the activation barrier, leading to a slower reaction.

**Table 1:** Results of the polymerization of (‐)‐menthide and (+)‐carvomenthide at different temperatures and with varying target degrees of polymerization (DP).^[a]^

Entry	Monomer	DP	*T* [°C]	Conv. [%]^[b]^	*M*_n, calc_ [g mol^−1^]^[c]^	*M*_n, SEC_ [g mol^−1^]^[d]^	*Ɖ* ^[d]^	*I* ^[e]^	TOF* [h^−1^]^[f]^
**1**	M	25	100	98	4200	13 900	1.22	0.30	365
**2**	M	25	80	>99	4300	10 200	1.16	0.42	184
**3**	M	25	60	>99	4300	8000	1.12	0.54	54
**4**	M	50	60	98	8300	19 100	1.04	0.44	114
**5**	M	100	60	97	16 500	24 000	1.04	0.69	84
**6**	M	25	40	96	4100	10 600	1.07	0.39	24
**7**	M	25	22	62	2700	6900	1.08	0.39	9
**8**	CM	25	100	94	4000	13 700	1.37	0.29	4473
**9**	CM	25	80	93	4000	13 800	1.28	0.29	3266
**10**	CM	25	60	97	4100	12 700	1.20	0.33	2060
**11**	CM	50	60	97	8200	13 700	1.20	0.60	1087
**12**	CM	100	60	96	16 300	23 600	1.07	0.69	1069
**13**	CM	25	40	94	4000	10 800	1.16	0.37	468
**14**	CM	25	21	95	4000	7700	1.12	0.52	156

^[a]^ All reactions were performed with [(ONOO)*^t^* ^Bu^Y(*i*PrO)(THF)] catalyst in 0.5 mL of toluene; M = (‐)‐menthide; CM = (+)‐carvomenthide; M was polymerized for 24 hours and CM for 3 hours.

^[b]^ Determined via ^1^H NMR spectroscopy.

^[c]^ Calculated from *M*_n, calc_ = (*M*_monomer_ x DP x conversion).

^[d]^ Relative molecular weights determined via size exclusion chromatography (SEC) in chloroform at 40 °C relative to polystyrene standards.

^[e]^ Initiator efficiency *I* = *M*_n, calc_/*M*_n, SEC_ with the relative obtained molar masses.

^[f]^ TOF adjusted using the calculated initiator efficiency based on the relative molecular weights as determined by SEC: TOF* = TOF/*I*.

In contrast, (+)‐carvomenthide polymerization proceeded significantly faster than (‐)‐menthide polymerization, overcoming the activation barrier more readily (Figures S40–S54; Figure 1b). As a result, reaction times were reduced to 3 hours. Data from Table 1 (Entries 8–10 and 13–14) show that, even at room temperature, polymerizations with a DP of 25 achieved high monomer conversions, indicating that (+)‐carvomenthide polymerization requires less energy to overcome its activation barrier. Compared to (‐)‐menthide, polydispersities were generally higher under the same conditions. This can be attributed to the faster reaction rate of (+)‐carvomenthide polymerization, which may lead to a less controlled process. Additionally, reduced steric hindrance in (+)‐carvomenthide could contribute to a greater variation in chain growth rates within the polymerization, further broadening the molecular weight distribution. Lower polydispersities were observed at lower temperatures for (‐)‐carvomenthide polymerizations due to reduced side reactions, consistent with the observed trend of transesterification. The linear increase in molar mass with conversion confirms the living nature of the polymerization for both monomers. For (+)‐carvomenthide, molecular weight versus time plots reveal deviations from the linear trend, indicating the onset of transesterification at different conversion thresholds: at 40 °C after 90% conversion (Figure S43), at 60 and 80 °C after 85% (Figures S45, S48), and at 100 °C already at 65% conversion (Figure S50). These deviations occur once a molecular weight of approximately 9000 g mol^−1^ is reached, suggesting that a molecular weight cut‐off is attained. This may indicate a critical chain length beyond which transesterification becomes kinetically favorable, likely due to increased chain mobility or enhanced intramolecular interactions. These observations further support the conclusion that transesterification is a temperature‐dependent phenomenon. At 100 °C, the ROP of (+)‐carvomenthide exhibited a TOF* of 4473 h^−1^, which decreased with lower temperature to 156 h^−1^ at room temperature. This trend reflects the temperature dependence of the reaction kinetics while maintaining high efficiency across all tested conditions for the polymerization of (+)‐carvomenthide. The higher reactivity of (+)‐carvomenthide can potentially lead to a less controlled polymerization compared to (‐)‐menthide.

In addition to temperature variations, the effect of different target degrees of polymerization on polymerization efficiency was investigated. Polymerizations were performed at 60 °C with target DPs of 25, 50, and 100 (Table 1, Entries 3–5 and Figure 1c for (‐)‐menthide; Table 1, Entries 10–12 and Figure 1d for (+)‐carvomenthide). Polymerization of both monomers was successful up to a DP of 100. For (‐)‐menthide, an increase in monomer feed (DP = 100) resulted in a slightly reduced conversion rate. This effect is likely due to side or termination reactions, which may be facilitated by trace impurities in the monomer.^[^ ^8^ ^]^ Despite this minor decrease in conversion, the polymerizations remained well‐controlled across all tested DPs, as indicated by consistently low dispersities (*Ð* ≤ 1.12 for (‐)‐menthide; *Ð* ≤ 1.20 for (+)‐carvomenthide). The TOF* values for (‐)‐menthide polymerization ranged from 54 h^−1^ (DP = 25) to 114 h^−1^ (DP = 50), showing an increase with higher monomer feed, probably due to improved coordination dynamics between the catalyst and the monomer since the amount of toluene was kept at the same amount. In contrast, for (+)‐carvomenthide, the TOF* decreased from 2060 h^−1^ (DP = 25) to 1069 h^−1^ (DP = 50) and remained nearly constant for DP = 100 (TOF* = 1090 h^−1^). This decrease could be attributed to the faster polymerization rate of (+)‐carvomenthide, leading to rapid monomer depletion near the catalyst active sites. Additionally, the decrease in TOF* could be linked to the molecular weight cut‐off, where transesterification or other chain‐transfer reactions become kinetically favorable at higher molecular weights, thus reducing the overall propagation rate. Moreover, at higher monomer‐to‐catalyst ratios, impurities in (+)‐carvomenthide may have a greater impact on catalyst deactivation and side reactions, contributing to the observed decrease in the TOF*. These trends suggest that TOF* is influenced not only by temperature but also by monomer concentration and purity.

**Figure 2:**
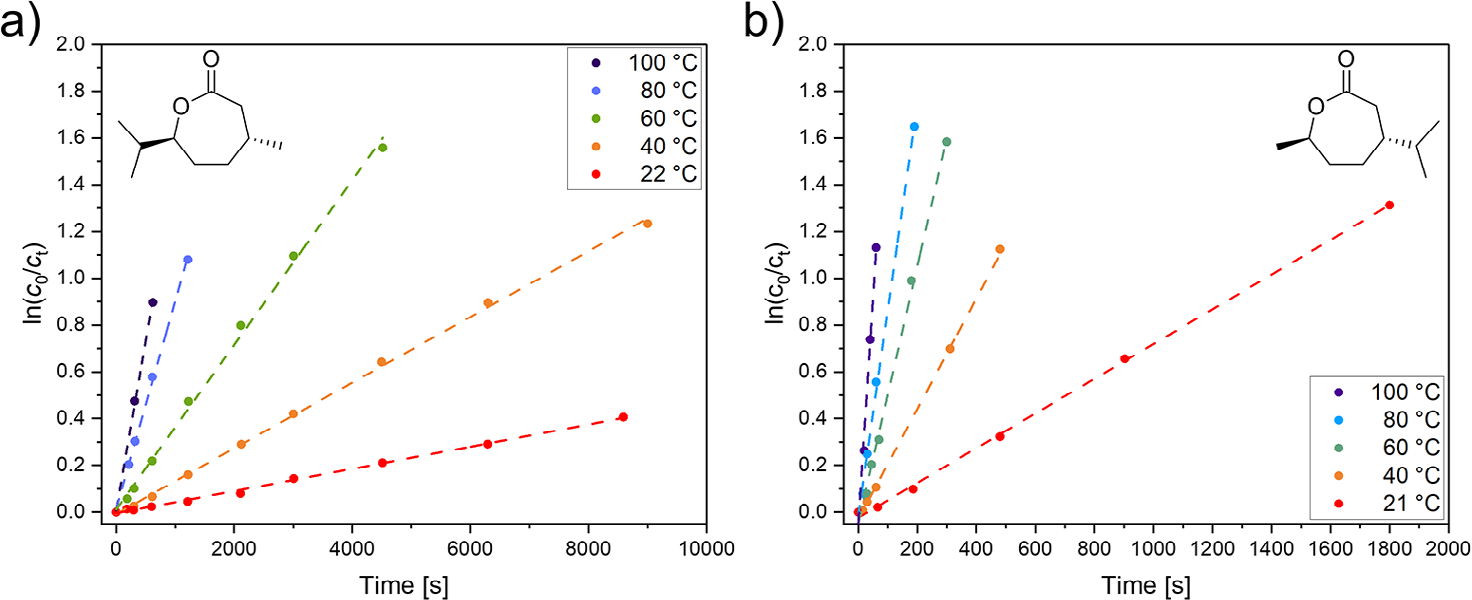
Linear first‐order kinetic plot for the polymerization of a) (‐)‐menthide and b) (+)‐carvomenthide at 100 °C, 80 °C, 60 °C, 40 °C, and room temperature.

A direct comparison of (‐)‐menthide and (+)‐carvomenthide polymerization reveals key differences in reactivity and control. Both monomers exhibit high conversions exceeding 93% under the tested conditions, except for (‐)‐menthide at room temperature, where conversion was limited to 62%. The polymerization of (‐)‐menthide demonstrated a high degree of control, as indicated by low polydispersities (*Đ* ≤ 1.22). In contrast, (+)‐carvomenthide exhibited slightly higher polydispersities (*Đ* ≤ 1.37), suggesting reduced control over molecular weight distribution. The significantly higher TOF* values observed for (+)‐carvomenthide confirm its faster polymerization kinetics, which can be attributed to a lower activation energy. However, the increased reaction rate also promotes side reactions. For (‐)‐menthide, transesterification became evident only at temperatures above 80 °C and conversions exceeding 90%. This is in accordance with previous block copolymerization studies with terpene‐based lactones, in which major transesterification reactions were not observed at optimized reaction conditions using ^13^C NMR spectroscopy.^[^ ^8^, ^20^ ^]^ In contrast, for (+)‐carvomenthide, transesterification was already observed at 40 °C after 90% conversion and at 100 °C as early as 65% conversion. This trend highlights a stronger temperature dependence of side reactions in (+)‐carvomenthide polymerization. Prior work on substituted lactones has shown that minor changes in substituent size and position can affect thermodynamic polymerization behavior. Compared to the unsubstituted ε‐caprolactone, (‐)‐menthide exhibited less favorable thermodynamic parameters, likely due to reduced ring strain and increased steric hindrance.^[^ ^21^ ^]^ Observed reactivity differences between (‐)‐menthide and (+)‐carvomenthide are probably due to kinetic factors related to steric interactions and transition state geometries. In (‐)‐menthide, the methyl group is located at position 4, while the isopropyl group is positioned at 7, adjacent to the ester group. In (+)‐carvomenthide, this arrangement is reversed, with the bulkier isopropyl group at position 4 and the smaller methyl group at position 7 near the ester group. The lower polymerization rate observed for (‐)‐menthide may arise from steric hindrance imposed by the isopropyl group adjacent to the ester group, which undergoes cleavage during polymerization. To further elucidate these differences, polymerization kinetics were analyzed to determine activation energies and Gibbs free energies, providing deeper insights into the mechanistic factors governing monomer reactivity. Data for the kinetic investigations of (‐)‐menthide polymerization are shown in Figures S25‐37, and (+)‐carvomenthide data can be found in Figures S40‐54. In both cases, monomer conversion initially increased linearly before reaching a plateau. For kinetic calculations, a linear first‐order kinetic plot was generated, utilizing the linear segment of the monomer conversion at different temperatures (Figure 2a for (‐)‐menthide and Figure 2b for (+)‐carvomenthide). Reaction rate constants *k*_p_, calculated from the slopes, are summarized in Table 2. Across all temperatures, (+)‐carvomenthide polymerized faster than (‐)‐menthide, as evidenced by its higher *k*_p_ values.

**Table 2:** Calculated reaction rate constants *k*_p_ from the slopes *m* from the linear first‐order kinetics plot (Figure 2), and the Gibbs free energy of activation Δ*G* ^‡^ for the polymerization of (‐)‐menthide (M) and (+)‐carvomenthide (CM).

Monomer	Temperature [°C]	*m* [s^−1^]	*k*_p_ [L (mol s)^−1^]	Δ*G* ^‡^ [kJ mol^−1^]
M	100	1.45E‐03	2.91E‐02	102.63
CM		1.93E‐02	3.88E‐01	94.93
M	80	8.88E‐04	1.81E‐02	99.17
CM		8.81E‐03	1.77E‐01	91.64
M	60	3.52E‐04	7.10E‐03	95.71
CM		5.48E‐03	1.10E‐01	88.35
M	40	1.40E‐04	2.81E‐03	92.25
CM		2.36E‐03	4.74E‐02	85.06
M	22 21	4.74E‐05	9.52E‐04	89.13
CM		7.42E‐04	1.49E‐02	81.94

The activation energies *E*_A_ were determined using the Arrhenius plots (Figures S55b, S56b). With slopes of ‐4908.58 K for (‐)‐menthide and ‐4366.55 K for the (+)‐carvomenthide polymerization and Equation S7, the activation energies yielded 40.8 kJ mol^−1^ for (‐)‐menthide and 36.3 kJ mol^−1^ for (+)‐carvomenthide. This 11% reduced activation energy for (+)‐carvomenthide indicates that its polymerization requires significantly less energy than for (‐)‐menthide.

**Figure 3:**
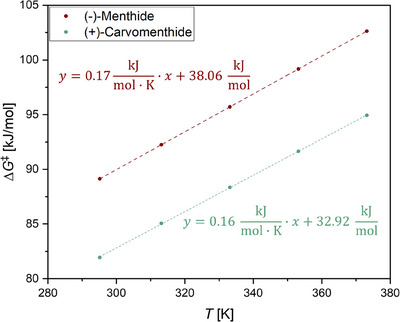
Gibbs free energy of activation Δ*G* ^‡^ for the ROP of (‐)‐menthide (red) and (+)‐carvomenthide (green).

Further thermodynamic parameters, including the enthalpy (Δ*H* ^‡^) and entropy (Δ*S* ^‡^) of activation, were calculated using the Eyring‐Polanyi plots with Equations S8 and S9 (Figures S57, S58). For (‐)‐menthide, Δ*H* ^‡^ and Δ*S* ^‡^ were determined to be 38.06 kJ mol^−1^ and ‐173.03 J (mol K)^−1^. For (+)‐carvomenthide, Δ*H* ^‡^ was 33.54 kJ mol^−1^, and Δ*S* ^‡^ was ‐164.53 J (mol K)^−1^. The higher enthalpy of activation for (‐)‐menthide suggests that more energy is required to induce its polymerization. Conversely, the smaller entropy change for (+)‐carvomenthide indicates a more favorable transition state. A reduced entropy change implies a closer resemblance between the initial state, including the monomer prior to activation, and its activated transition state, facilitating the polymerization process. Utilizing the calculated enthalpy and entropy values, the Gibbs free energy of activation (Δ*G* ^‡^) was determined across all temperatures with the Gibbs‐Helmholtz equation (Equation S3) (Table 2; Figure 3). The Gibbs free energy reflects the energy difference between the resting state of the polymerizing system and the activated transition state. Across all tested temperatures, (+)‐carvomenthide exhibited lower Δ*G* ^‡^ values than (‐)‐menthide, further confirming its reduced energy barrier for polymerization.

The observed kinetic and thermodynamic disparities can be attributed to the structural differences between the monomers. In (‐)‐menthide, the isopropyl group is positioned near the ester group that is cleaved during polymerization, introducing steric hindrance. In contrast, the smaller methyl group in (+)‐carvomenthide reduces steric hindrance near the ester group, allowing for more efficient chain propagation and faster reaction rates. These findings highlight the impact of substituent positioning on monomer reactivity and energy barriers in ROPs.

**Figure 4:**
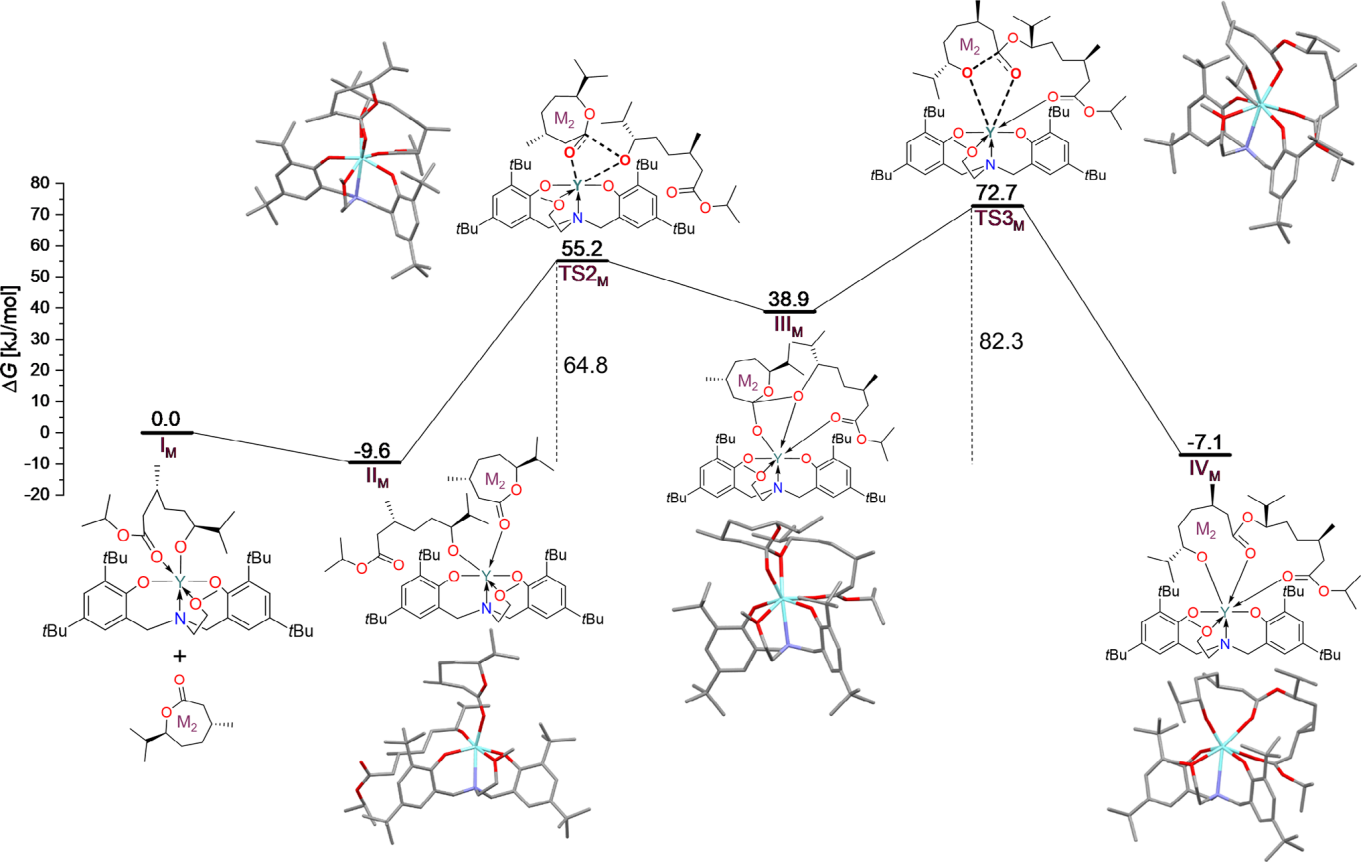
The Gibbs free energy profile (kJ mol^−1^) for ROP of (‐)‐menthide by investigating the second monomer insertion (M_2_). The bold dashed lines in **TS2_M_** and **TS3_M_** represent bond breaking and forming for the particular step. Hydrogen atoms are omitted from optimized geometries for clarity. Atoms are color‐coded as follows: C (grey), O (red), N (blue), and Y (cyan).

**Figure 5:**
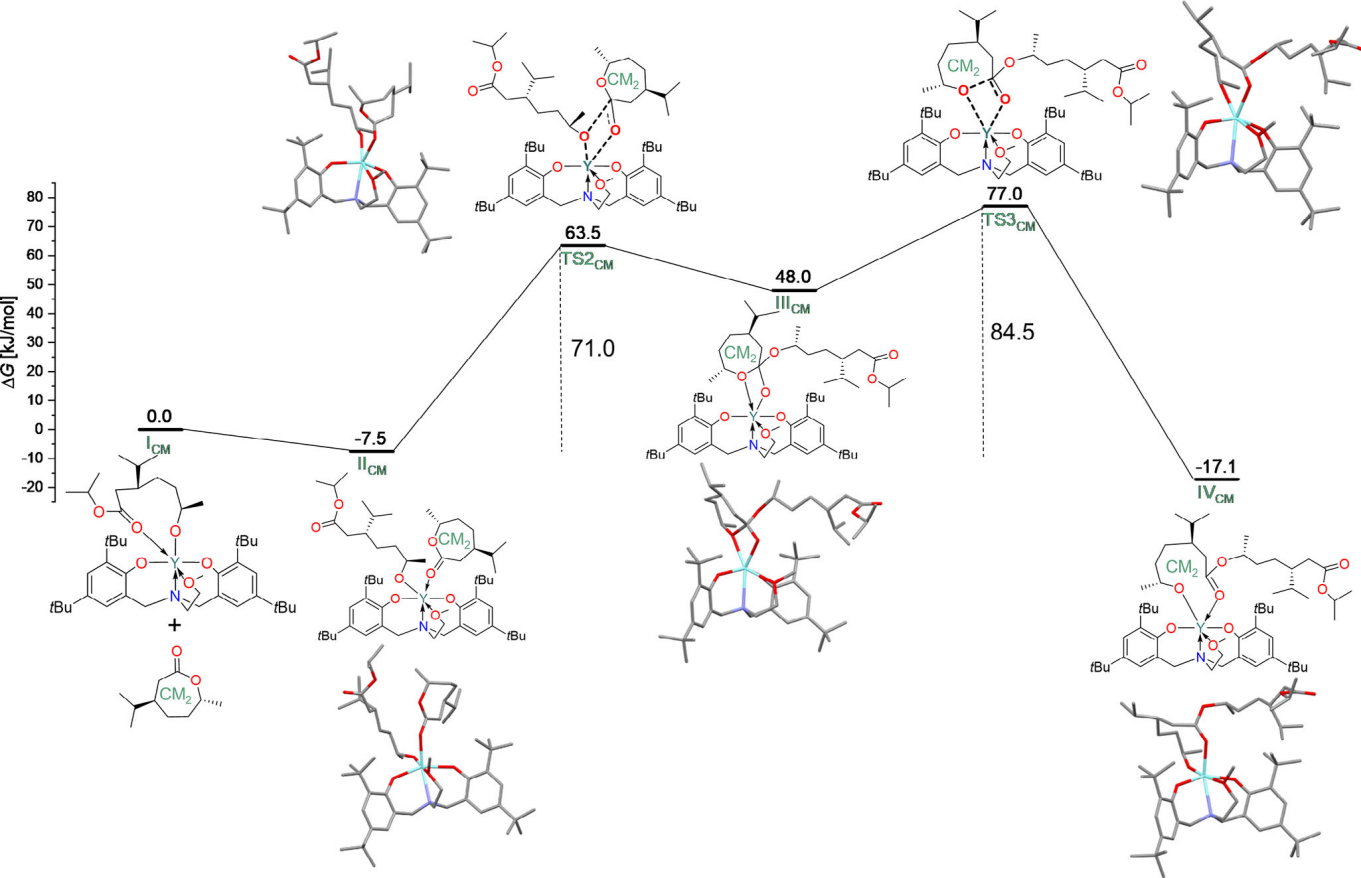
The Gibbs free energy profile (kJ mol^−1^) for ROP of *trans* (+)‐carvomenthide by investigating the second monomer insertion (CM_2_). The bold dashed lines in **TS2_CM_** and **TS3_CM_** represent bond breaking and forming. Hydrogen atoms are omitted from optimized geometries for clarity. Atoms are color‐coded as follows: C (grey), O (red), N (blue), and Y (cyan).

To further understand the underlying mechanisms, the chain elongation processes in the ROPs of (‐)‐menthide and (+)‐carvomenthide were analyzed by means of DFT calculations (BP86‐D3/def2‐TZVP toluene as implicit solvent) in detail. By focusing on the key steps and comparing their respective energy profiles (Figure 4 for (‐)‐menthide and Figure 5 for (+)‐carvomenthide), insights into the efficiency of chain propagation and the influence of steric hindrance were gained. Since (+)‐carvomenthide exists as diastereomers, the reaction mechanism for ROP was additionally investigated with regard to influences by stereoisomers, and DFT calculations were performed for both *trans* and *cis* (+)‐carvomenthide (Figures 5 and S59). Nevertheless, mainly the *trans* isomer is experimentally relevant, with up to 4% contamination by the *cis* isomer. For all calculations, the focus was set on simulating the propagation step rather than the initiation process. Thus, the polymer chain was modeled with the first inserted monomer unit coordinated to the yttrium complex, which then acts as the nucleophilic alkoxide species (systems **I_M_** and **I_CM_**).

In the initial step of (‐)‐menthide polymerization, coordination of the carbonyl group of the incoming (‐)‐menthide monomer (**M_2_**) to the yttrium center in intermediate **I_M_** leads to the formation of intermediate **II_M_**. This coordination can occur through various modes, such as equatorial or axial binding, and via the re‐ (i.e., the isopropyl group in monomer M_2_ pointing to the back) or the si‐face (i.e., the isopropyl group in M_2_ pointing to the front). Coordination of **M_2_** replaces the carbonyl‐metal coordination of the penultimate unit of the existing polymer chain, leading to the formation of **II_M_**. In the free energy profile in Figure 4, the most stable conformation, **II_M_**, was considered, featuring equatorial coordination of **M_2_** to the yttrium center and via its re‐face. The transformation is exergonic with a free energy change of ‐9.6 kJ mol^−1^, showing that monomer coordination is favored in the course of propagation. Calculations, as presented in Figure S60 (Supporting Information), suggest that the conformation **II_M_** shown in Figure 4 is more stable than its alternatives, with relative free energies with respect to **II_M_** of 11.1 (**II_M_, _si_**), 8.6 (**II_M, axial_)**, and 7.5 (**II_M, κ‐3_**) kJ mol^−1^. These correspond to conformations where the ester ring oxygen of **M_2_** is oriented si (**II_M, si_**), **M_2_** is bound at the axial position (**II_M, axial_**), and with an additional coordination from the carbonyl oxygen atom of the first inserted monomer to the yttrium center (**II_M, κ‐3_**). The axial coordination of (−)‐menthide was also examined with re/si‐facial rotation; however, only the configuration shown in Figure S60 was sterically feasible and was therefore considered.

Similarly, for the coordination of *trans* (+)‐carvomenthide, conformations with axial binding (**II_CM, axial_**) and si‐facial orientation (**II_CM, si_**) are less stable relatively by 11.9 and 4.9 kJ mol^−1^ (Figure S61), compared to **II_CM_** (Figure 5), which features equatorial coordination of **CM_2_** to the yttrium center via its re‐face. A conformation of **II_CM_** that includes additional coordination from the carbonyl oxygen atom of the first inserted monomer to the yttrium center is not observed, likely due to steric hindrance and the stereochemistry of the isopropyl group. Therefore, in the free energy profile of Figure 5, the most stable conformation for **II_CM_** was considered, which is a slightly less exergonic process (‐7.5 kJ mol^−1^) than the formation of **II_M_**.

**Figure 6:**
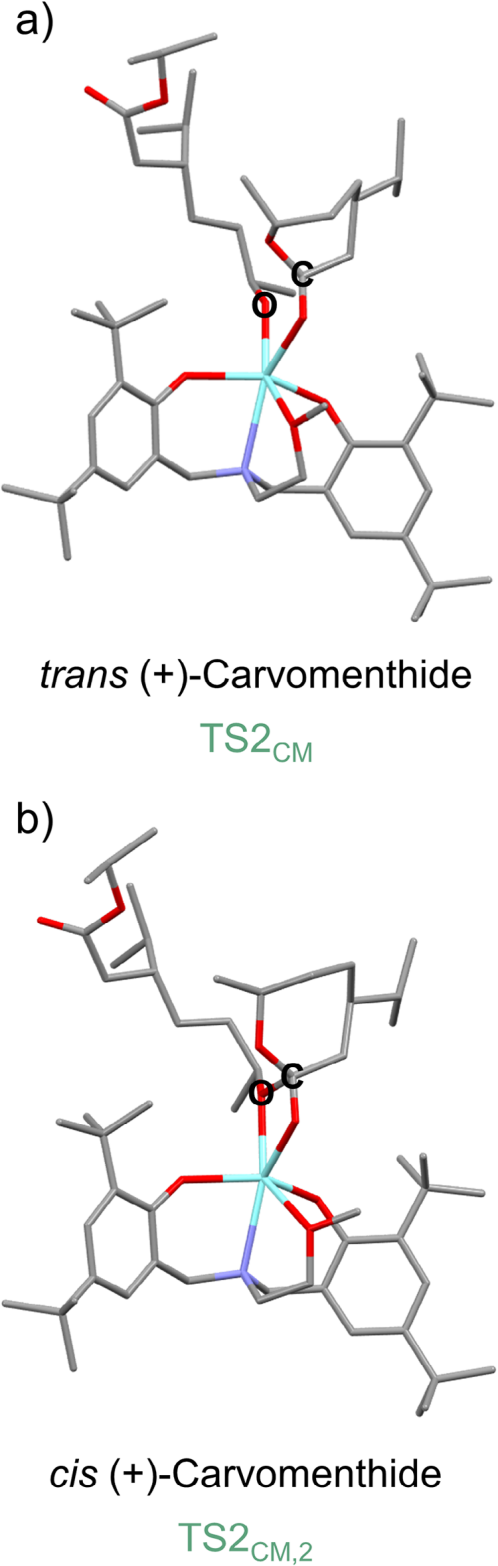
Four‐membered transition states for the ROP of *trans* (+)‐carvomenthide (a; **TS2_CM_**) and *cis* (+)‐carvomenthide (b; **TS2_CM,2_**). The nucleophilic alkoxide attacks the carbonyl carbon of the new monomer via a four‐membered transition state. The atoms involved in the C‐O bond formation between the carbonyl carbon (C) and the alkoxide nucleophile (O) are assigned. Hydrogen atoms are omitted from optimized geometries of both transition states for clarity. Atoms are color‐coded as follows: C (grey), O (red), N (blue), and Y (cyan).

In the subsequent nucleophilic attack, the alkoxide nucleophile is transferred from the yttrium center to the carbonyl carbon atom of the newly coordinated monomer (M_2_ or CM_2_) via a four‐membered cyclic transition state. For (‐)‐menthide, this transition state (**TS2_M_**) leads to the formation of the tetrahedral intermediate **III_M_**. The calculated free energy barrier for this step (**II_M_** to **TS2_M_**) is 64.8 kJ mol^−1^. For *trans* (+)‐carvomenthide, the nucleophile transfer occurs similarly via **TS2_CM_**, leading to the intermediate **III_CM_**. For *cis* (+)‐carvomenthide, the respective structures are **TS2_CM, 2_** and **III_CM, 2_**. The *cis* (+)‐carvomenthide shows a lower calculated free energy barrier from **II_CM, 2_** to **TS2_CM, 2_** of 53.5 kJ mol^−1^, highlighting the influence of steric interactions and stereochemistry on the polymerization kinetics. The transition states for both (+)‐carvomenthide isomers are shown in detail in Figure 6. The C─O bond distances between the carbonyl carbon atom and the alkoxide nucleophile in the respective monomer within the four‐membered transition states **TS2_CM, 2_**, **TS2_M_**, and **TS2_CM_** are 1.70 Å, 1.87 Å, and 2.00 Å. The shortest bond length in **TS2_CM, 2_** may contribute to its lower activation barrier. After the formation of the tetrahedral intermediates, the ester carbonyl O‐atom of the first inserted monomer coordinates to the yttrium center during (‐)‐menthide polymerization (**III_M_**); however, this coordination is not observed in **III_CM_** and **III_CM, 2_**.

The final step in the mechanism involves the ring‐opening of the cyclic monomer. For (‐)‐menthide, a step proceeds through the cleavage of the C‐O bond via **TS3_M_**, where the carbonyl oxygen of the ester moiety coordinates to the yttrium center, forming system **IV_M_**. The free energy barrier for that ring‐opening step is 82.3 kJ mol^−1^, and the overall cycle is exergonic with a free energy change of ‐7.1 kJ mol^−1^. For *trans* (+)‐carvomenthide, the ring‐opening follows a similar mechanism via **TS3_CM_**, leading to **IV_CM_**. The free energy barrier for that step is 84.5 kJ mol^−1^, but it is significantly lower for the *cis* isomer (13.8 kJ mol^−1^). Additionally, the free energy changes of the overall cycle are ‐17.1 kJ mol^−1^ for the *trans* and ‐29.3 kJ mol^−1^ for the *cis* isomer, reflecting a more favorable exergonic process for the *cis* configuration. Furthermore, in both *cis* and *trans* (+)‐carvomenthide, the carbonyl oxygen of the ester group of the first incorporated monomer does not coordinate to the yttrium center, whereas in (‐)‐menthide, this coordination is maintained. This distinction may further contribute to the observed kinetic and experimental differences. The lack of metal‐carbonyl coordination of the penultimate unit in **IV_CM_** may influence the electronic environment of the growing chain end, potentially affecting the propagating species. This could, on the one hand, lead to a faster reaction by faster coordination of the next monomer, but it could, on the other hand, also lead to higher chances of side reactions due to a free binding site on the metal center.

The energetic span model is used to identify the rate‐determining intermediates and transition states.^[^ ^22^ ^]^ The resulting energetic span can then directly be compared to the experimentally determined Gibbs free energy of activation Δ*G* ^‡^. The rate‐determining step for the polymerization of (‐)‐menthide was identified as the difference between **TS3_M_** and **II_M_** with a calculated Δ*G* ^‡^_DFT_ of 82.3 kJ mol^−1^. For *trans* (+)‐carvomenthide, the rate‐determining step is between **TS3_CM_** and **II_CM_** with Δ*G* ^‡^_DFT_ = 84.5 kJ mol^−1^. A significantly lower overall free‐energy barrier of only Δ*G* ^‡^_DFT_ = 53.5 kJ mol^−1^ was found for *cis* (+)‐carvomenthide, with the rate‐determining step being between **TS2_CM,2_** and **II_CM,2_**. Thus, overall, the lowest barrier was found for *cis* (+)‐carvomenthide. Since *trans* (+)‐carvomenthide dominates the experiments and the polymerization of the *cis* isomer is expected to be faster, small amounts of *cis* isomer should not hinder efficient polymerization. The comparison also clearly shows that the Gibbs free energy barrier for the rate‐determining step of (‐)‐menthide (82.3 kJ mol^−1^) and that of *trans* (+)‐carvomenthide (84.5 kJ mol^−1^) agree well with the experimental data presented above. While the computed value indicated a slightly higher barrier for *trans* (+)‐carvomenthide than for (‐)‐menthide, this contradicts the experimental observations. However, the difference between the two calculated barriers is small and lies within the expected error margin of the computational method. To obtain more accurate energy estimates, single‐point energy calculations were performed using the more accurate hybrid functional B3LYP with D3‐BJ dispersion corrections (Figures S62 and S63). These calculations were carried out at the B3LYP‐D3BJ/def2‐TZVP//BP86‐D3/def2‐TZVP level of theory and included implicit solvation in toluene. The refined free energy barriers were determined to be 102.1 kJ mol⁻^1^ for (‐)‐menthide and 84.9 kJ mol⁻^1^ for *trans* (+)‐carvomenthide, qualitatively agreeing with the experimental trend, which indicates a lower free energy barrier for (+)‐carvomenthide compared to (‐)‐menthide. These calculations also suggest that the formation of **IV_CM_** is not only kinetically but also thermodynamically more favorable than the formation of **IV_M_**, further supporting the experimental observation of a faster and more favorable (+)‐carvomenthide polymerization.

This analysis highlights the crucial role of both the substitution pattern and stereochemistry in ROP. The steric effects of the isopropyl group, particularly in *cis* (+)‐carvomenthide, reduce steric hindrance around the transition states, facilitating chain propagation and lowering energy barriers. Moreover, the trend in the observed free energy barrier in (‐)‐menthide, *trans* (+)‐carvomenthide, and *cis* (+)‐carvomenthide is attributed to the C‐O bond distance between the carbonyl carbon atom and the alkoxide nucleophile (Figure 6). Shorter C‐O bond distances in (‐)‐menthide and *cis* (+)‐carvomenthide polymerization stabilize the transition states more than the longer C‐O bond distance in *trans* (+)‐carvomenthide polymerization. Additionally, the absence of coordination between the carbonyl oxygen of the ester group of the opened monomer of the existing chain in **IV_CM_** and **IV**_CM,2_, unlike in **IV_M_**, may further influence polymerization kinetics and experimental outcomes. In these cases, the isopropyl group likely prevents coordination of the carbonyl group of the penultimate unit in the polymer chain, potentially promoting side reactions due to unoccupied coordination sites.

## 3 Conclusion

The lactones (‐)‐menthide and *trans* (+)‐carvomenthide were synthesized from the terpenoids (‐)‐menthone and (+)‐dihydrocarvone, differing only in their substitution pattern. The ROP kinetics of these terpene‐based seven‐membered lactones were investigated with polymerizations using the [(ONOO)*^t^* ^Bu^Y(*i*PrO)(THF)] catalyst under varying temperatures and monomer‐to‐catalyst ratios. All reactions proceeded in a controlled manner, as evidenced by relatively low polydispersity values (*Ð* ≤ 1.34). At higher temperatures and high conversions, increased polydispersities suggest the occurrence of side reactions. The experiments revealed differences in the polymerization kinetics of the two lactones despite their identical molecular weights and compositions. However, their substitution patterns differ from each other, with (‐)‐menthide bearing a methyl group at position 4 and an isopropyl group at position 7 near the ester, while (+)‐carvomenthide features the opposite arrangement. (‐)‐Menthide polymerization required 24 hours to reach high conversions and achieved a TOF* of 30 h^−1^ at 60 °C (DP = 25). In contrast, the reaction time for ROP of (+)‐carvomenthide yielded substantially higher TOFs* at all temperatures (e.g., 60 °C, DP = 25, TOF* = 2060 h^−1^). The activation energy, enthalpy, entropy, and Gibbs free energy of activation were determined, revealing that the polymerization of *trans* (+)‐carvomenthide required less energy than that of (‐)‐menthide. However, enhanced reactivity also led to an increase in polydispersity, probably due to enhanced transesterification reactions. DFT calculations were performed for the reaction pathways of both monomers, as well as two diastereomers of (+)‐carvomenthide, that is, the *trans* (4*R*, 7*R*) and *cis* (4*R*, 7*S*) isomers. The DFT results confirmed mechanistic differences in chain propagation for (‐)‐menthide and (+)‐carvomenthide and also between the *cis* and *trans* isomer of (+)‐carvomenthide. The experimental Gibbs free energy of activation for the ROP of (‐)‐menthide was determined to be 89.1 kJ mol^−1^ at 22 °C, aligning closely with the theoretically calculated value of 82.3 kJ mol^−1^. For *trans* (+)‐carvomenthide, the experimental Gibbs free energy of activation was calculated as 81.9 kJ mol^−1^ at 22 °C. Additionally, the theoretical energy barrier for the ROP of *trans* (+)‐carvomenthide was calculated to be 84.5 kJ mol^−1^, while that of the *cis* isomer was significantly lower with 53.5 kJ mol^−1^, indicating a clear kinetic preference for the *cis* isomer. Since the monomer mixture used in polymerization predominantly contains the *trans* isomer, the presence of small amounts of the *cis* isomer is not expected to impede efficient polymerization. The lack of coordination of the carbonyl group of the penultimate unit in the polymer chain observed in both (+)‐carvomenthide isomers, but not in (‐)‐menthide, may promote a faster propagation but also side reactions, further affecting the polymerization kinetics. Overall, this study provides a comprehensive understanding of the ROP mechanisms of (‐)‐menthide and (+)‐carvomenthide, demonstrating the critical influence of molecular structure and stereoinformation on the energy requirements, reaction rates, and control of these processes. These insights offer guidance for the design and optimization of terpene‐based lactone polymerizations, paving the way for more efficient and sustainable materials development.

## Supporting Information

The authors have cited additional references within the Supporting Information.^[^ ^23^ ^]^

## Conflict of Interest

The authors declare no conflict of interest.

## Supporting information



Supporting Information

## Data Availability

The data that support the findings of this study are available in the supplementary material of this article.
