# Supplementary material for: Influence of the Substitution Pattern of Terpene‐Based Seven‐Membered Lactones on Yttrium‐Mediated Ring‐Opening Polymerization: A Kinetic and Mechanistic Investigation
Source: Chemistry. 2025 Jun 16;31(38):e202501380. doi: 10.1002/chem.202501380 (PMC12238913; doi:10.1002/chem.202501380)
Supplement: Supplementary file 1 — Supporting Information [file CHEM-31-e202501380-s001.pdf]

Supporting Information

Influence of the Substitution Pattern of Terpene-Based Seven-Membered Lactones on Yttrium-Mediated Ring-Opening Polymerization: A Kinetic and Mechanistic Investigation

Lea-Sophie Hornberger,[a] Svenja Hiotidis,[a] Shailja Jain,[b] Michael Benz,[c] Hugo Montan,[a] Deven P. Estes,[c] Johannes Kästner[b] and Friederike Adams*[a,d,e]

1. Theory

The Arrhenius plot can be used to determine the activation energy of a reaction. With the pre-factor A, the activation energy E_A, the ideal gas constant R and the temperature T, the Arrhenius equation is:

$$k = A \cdot \exp\left(-\frac{E_A}{RT}\right) \tag{S1}$$

The graphical representation of Equation S1 is called the Arrhenius plot (Figure S1a). The molar Gibbs energy (free enthalpy) $\Delta G^{\ddagger}$ signifies the energy barrier between the monomers and an active center, often conceptualized as a transition state. The relation between the Gibbs energy and the rate constant k is described in the Eyring-Polanyi equation[1]:

$$k = \frac{k_{\mathrm{B}}T}{h} \exp\left(-\frac{\Delta G^{\ddagger}}{RT}\right) \tag{S2}$$

k_B is the Boltzmann-constant and h Planck's constant. Eyring's assumptions are based on the transition state theory (TST) (Figure S1c). The Gibbs energy ΔG is defined via the Gibbs-Helmholtz equation as:

$$\Delta G = \Delta H - T \cdot \Delta S \tag{S3}$$

with the enthalpy ΔH and the entropy ΔS. With the Gibbs-Helmholtz equation (Equation S3), Equation (S2) is:

$$k = \frac{k_{\mathrm{B}}T}{h} \exp\left(-\frac{\Delta H^{\ddagger}}{RT} + \frac{\Delta S^{\ddagger}}{R}\right) \tag{S4}$$

where $\Delta H^{\ddagger}$ is the enthalpy and $\Delta S^{\ddagger}$ the entropy of the activation. By rearranging Equation (S2),

$$\ln\left(\frac{k}{T}\right) = -\frac{\Delta H^{\ddagger}}{R} \cdot \frac{1}{T} + \ln\left(\frac{k_B}{h}\right) + \frac{\Delta S^{\ddagger}}{R} \tag{S5}$$

is obtained, its plot is known as the Eyring-Polanyi plot (Figure S1b).

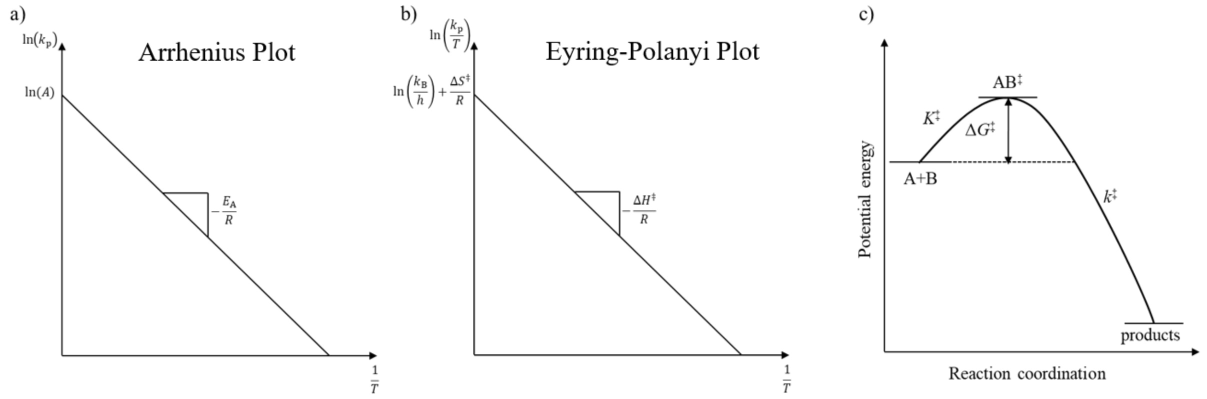

Figure S1: a) Schematic diagram of the Arrhenius-plot; b) Schematic diagram of the Eyring-Polanyi-plot; c) Energy diagram with transition state AB‡, rate constants $K^‡$, and $k^‡$, and Gibbs energy of activation $\Delta G^‡$.

For the kinetic calculations in this research, a linear first-order kinetic plot was created using only the linear portion of the conversion growth (Figure 2). The slope m was used to calculate the reaction rate k_p:

$$k_\mathrm{p} = \frac{m}{[I]_0} \tag{S6}$$

$[I]_0$ is the initial concentration of the catalyst. Further, the Arrhenius plot was used for the determination of the activation energy E_A (Figures S55 and S56). The activation energy was derived from the slope of the resulting linear trend line:

$$E_\mathrm{A} = -(m \cdot R) \tag{S7}$$

The enthalpy and entropy of activation were determined with the Eyring-Polanyi plot (Figures S57 and S58). The enthalpy of activation ($\Delta H^‡$) was calculated from the slope using:

$$\Delta H^‡ = -(m \cdot R) \tag{S8}$$

The entropy of activation ($\Delta S^‡$) was determined by the intersection c with the y-axis:

$$\Delta S^‡ = R \cdot \left(c - ln\left(\frac{k_\mathrm{B}}{h}\right)\right) \tag{S9}$$

Literature Data for the Ring-Opening Polymerization of (-)-Menthide and (+)-Carvomenthide

Table S1: Reported studies on ring-opening polymerization of (-)-menthide (M) and (+)-carvomenthide (CM) with different catalytic systems, including key experimental parameters.

**Monomer**	**Catalytic System**	**Eq.**	**Solv.**	***T*** **[°C]**	***t*** **[h]**	**Conv. [%]**	**$M_{n,SEC}$ [kg mol^{-1}]**	***Đ***	**Ref.**	**Year**
**M**	Zn alkoxide	100	Tol	21	16	96	41.6	1.4	[2]	2005
**M**	$ZnEt_2$, DEG	80	Tol	100	4.5	73	20.0	1.3	[3]	2007
**M**	$Sn(Oct)_2$, DEG	635	Tol	135	72	95	95.0	1.1	[4]	2011
**M**	$Mg(BHT)_2(THF)_2$	50	Tol	80	96	71	8.0	1.3	[5]	2015
**M**	$[(ONOO)^{tBu}Y$ (bdsa)(THF)]	100	Tol	100	1.5	82	19.0	1.1	[6]	2020
**M**	$[(ONOO)^{tBu}Y$ (bdsa)(THF)]	100	Tol	RT	18	63	33.4	1.1	[6]	2020
**M**	CTPB organocatalyst, BnOH	100	Tol	110	5	84	18.4	1.7	[7]	2020
**CM**	$ZnEt_2$, BnOH	100	bulk	100	8	95	14.1	1.1	[8]	2011
**CM**	$Sn(Oct)_2$, DEG	74	bulk	100	22	94	17.4	1.1	[9]	2015

DEG = diethylene glycol; Oct = ethylhexanoate; BHT = 2,6-di-tert-butyl-4-methylphenoxide; CTPB = cyclic trimeric phosphazene base; BnOH = benzyl alcohol

2. Materials and Methods

Materials:

All chemicals were purchased from *Thermo Fischer Scientific, Tokio Chemical Industry Germany* or *Sigma-Aldrich* and were used as received unless otherwise stated. Toluene was dried using a solvent purification system (*MBraun*) and stored inside an argon-filled glovebox (*MBraun EcoLab*) over molecular sieves. All polymerization reactions were performed under an argon atmosphere using Schlenk technique or in a glovebox. The synthesis of the amino-alkoxy-bis(phenolate) yttrium amido complex [(ONOO)^{tBu}Y(bdsa)(THF)] was performed according to literature.[10] It involved the synthesis of an yttrium-bdsa-precursor[11] ($Y(bdsa)_3(THF)_2$, bdsa = bis(dimethylsilyl)amide)) and the 2-methoxyethylamino-bis(phenolate) ligand[12].

Analysis:

Nuclear magnetic resonance (NMR) spectra were recorded on a *Bruker* Avance III 400 or *Bruker* Avance III HD 700 NMR spectrometer with a 176, 400, or 700 MHz magnet. Chemical shifts δ are reported in ppm and calibrated to the residual proton signal of the deuterated solvent in which the substance was dissolved.

Size-exclusion chromatography (SEC) was measured in chloroform with an SEC setup including one *PSS* SDV 5 µm 8*50 mm guard column and three *PSS* SDV 100,000 Å 5 µm 8*50 mm columns, heated at 40 °C. The substances were detected with a refractive index detector (*Agilent* 1200 Series G1362A) connected to the columns. All samples were prepared with a concentration between 2 and 4 mg mL^{-1}. Molar masses were determined relative to polystyrene standards.

For gas chromatography-mass spectrometry (GC-MS), an *Agilent* 5977D system was used. The sample concentration was 2 mg mL^{-1} in acetone.

Electrospray ionization-mass spectrometry (ESI-MS) was performed with either a *Varian* 500-MS spectrometer or a micrOTOF-Q *Bruker* Daltonics in positive ionization mode. The samples were dissolved in acetonitrile.

Atmospheric pressure chemical ionization-mass spectrometry (APCI-MS) was performed with a *Thermo Scientific* Q Exactive Plus Orbitrap MS spectrometer in positive ionization mode. Samples were dissolved in acetonitrile.

Synthesis of (-)-Menthide:
(-)-Menthide was synthesized from (-)-menthone (97%, *thermo scientific*, LOT: 10237765) using a literature procedure.[4, 8]

^{1}H NMR (700 MHz, $CDCl_3$, 298 K): δ [ppm] = 3.99 (dd, J = 9.3, 4.4 Hz, 1H), 2.52 – 2.45 (m, 1H), 2.39 (dd, J = 13.4, 1.8 Hz, 1H), 1.90 – 1.85 (m, 1H), 1.52 (dddd, J = 15.8, 12.8, 9.2, 3.1 Hz, 1H), 1.28 – 1.19 (m, 1H), 0.97 (d, J = 6.9 Hz, 3H), 0.91 (d, J = 7.0 Hz, 3H), 0.89 (d, J = 6.8 Hz, 3H).
^{13}C NMR (176 MHz, $CDCl_3$, 298 K, 512 scans): δ [ppm] = 17.0, 18.3, 23.9, 30.4, 30.9, 33.3, 37.4, 42.5, 84.6, 174.9.

Elemental Analysis:

	C	H
**Calculated [%]**	70.55	10.66
**Found [%]**	70.62	10.66

Synthesis of (+)-Carvomenthide:
(+)-Dihydrocarvone was purchased from *Sigma-Aldrich* as a mixture of isomers (≥97%, LOT number: SHBP1822) and used as received. (+)-Dihydrocarvide was synthesized from (+)-dihydrocarvone according to literature.[8] The obtained (+)-dihydrocarvide was isolated as the *trans* isomer (4*R*,7*R*)-4-isopropenyl-7-methyloxepan-2-one ((4*R*,7*R*)-dihydrocarvide) in a yield of 21% as a colorless liquid. The (+)-dihydrocarvide (2.4 g, 1 eq., 14.3 mmol) was dissolved in ethyl acetate (15 mL) and mixed with one spatula of palladium on carbon (Pd/C, 10% Pd). This mixture was transferred into an autoclave and stirred under H_2 pressure (15 – 30 bar) at room temperature for 3 days. The catalyst was removed using an aluminum oxide column with ethyl acetate as eluent. The solvent was removed in vacuo and the resulting (+)-carvomenthide (94% yield, 2.25 g) was obtained as a colorless oil and was further purified via Schlenk vacuum distillation (T_{Oil} = 122 °C; T_{Head} = 93 °C; p = 2.55 · 10^{-2} mbar).

^{1}H NMR: (700 MHz, $CDCl_3$, 298 K): δ [ppm] = 4.40 (dq, J = 9.2, 6.4 Hz, 1H), 2.47 – 2.41 (m, 2H), 1.89 (dt, J = 15.3, 3.9 Hz, 1H), 1.79 (dd, J = 13.5, 3.7 Hz, 1H), 1.63 (dddd, J = 24.9, 12.9, 10.1, 5.0 Hz, 2H), 1.54 (qq, J = 10.4, 6.6, 5.1 Hz, 1H), 1.44 (td, J = 12.8, 3.7 Hz, 1H), 1.32 (d, J = 6.5 Hz, 3H), 0.87 (d, J = 7.0 Hz, 3H), 0.84 (d, J = 6.9 Hz, 3H).
^{13}C NMR: (176 MHz, $CDCl_3$, 512 scans) δ [ppm] = 18.6, 18.8, 22.6, 31.3, 33.6, 35.8, 38.1, 40.3, 76.7, 175.7.

Elemental Analysis:

	C	H
**Calculated [%]**	70.55	10.66
**Found [%]**	70.43	10.75

Homopolymerizations with [(ONOO)^{tBu}Y(iPrO)(THF)]:

For the polymerization of the two monomers, (-)-menthide and (+)-carvomenthide, 24.9 µmol of the amino-alkoxy-bis(phenolate) yttrium amido complex [(ONOO)^{tBu}Y(bdsa)(THF)] (1 eq., 19.94 mg) was dissolved in 0.5 mL of toluene. 1 eq. of isopropanol (24.9 µmol, 1.9 µL) was added to the solution and stirred for five minutes at room temperature to give [(ONOO)^{tBu}Y(*i*PrO)(THF)]. The required equivalents of monomer were weighed into a screw cap vial in a glovebox. The catalyst mixture was drawn into a syringe and the cannula was inserted through the septum of the vial containing the monomer. The vial was removed from the glovebox and transferred to a heating block in the fume hood. The monomer was preheated to the respective temperature and the catalyst was added in one portion. The mixture was stirred for the respective time. To quench the reaction, 1.5 mL of deuterated chloroform was added, and an aliquot was taken to determine the conversion using ^{1}H NMR spectroscopy. The polymer was precipitated in methanol. It was filtered and dried under vacuum. The molar mass and polydispersity of the resulting polymer were measured with SEC relative to polystyrene standards in chloroform.

Kinetic measurements of the homopolymerization with [(ONOO)^{tBu}Y(iPrO)(THF)]:

The kinetic measurements for both monomers, (-)-menthide and (+)-carvomenthide, were conducted with an aliquot method. 49.8 µmol (1 eq., 39.89 mg) of [(ONOO)^{tBu}Y(bdsa)(THF)] was dissolved in 0.5 mL toluene. 1 eq. of isopropanol (49.8 µmol, 3.8 µL) was added to the solution and stirred for five minutes at room temperature to give [(ONOO)^{tBu}Y(*i*PrO)(THF)]. The respective equivalents of monomer were weighed into a screw cap vial and dissolved in 0.5 mL toluene. The catalyst solution was drawn into a syringe and the cannula was inserted through the septum of the vial containing the monomer. The vial was removed from the glovebox and transferred to a heating block in the fume hood. The monomer was preheated to the respective temperature and the catalyst was added in one portion. After regular time intervals, aliquots were taken from the reaction solution and quenched by adding 0.5 mL $CDCl_3$. From each aliquot, the conversion was determined via ^{1}H NMR spectroscopy, and SEC was used to determine the molar mass and polydispersity of each sample.

Computational Details:

The quantum chemical calculations were conducted using density functional theory (DFT) in Turbomole version 7.4.1.[13] The Becke-Perdew-86 (BP86)[14] functional with D3 dispersion correction[15] and the def2-TZVP[16] basis set has been employed. We report energies in a solvent, taken into account by using the COSMO model[17] with a relative dielectric constant ε = 2.38 for toluene. The resolution of identity (RI),[18] along with the multipole accelerated resolution of identity (MARI-J)[19] approximations, has been used for an accurate and efficient treatment of the electronic Coulomb term in the DFT calculations (for both; i) geometry optimization at BP86-D3/def2-TZVP, and ii) single-point calculations at B3LYP[20]-D3BJ/def2-TZVP and BP86-D3/def2-TZVP). The energy values reported are Δ*G* values at 298.15 K with a reference concentration of 1 mol L^{-1}. Harmonic frequency calculations were performed for all stationary points to confirm them as local minima or transition state structures. Furthermore, intrinsic reaction coordinate (IRC) calculations were carried out with all transition states to further confirm that they connect to the correct reactant and product geometries. Furthermore, to determine the energetic span of the reaction, we employed the energetic span model (ESM) developed by Shaik and Kozuch[21] for all free energy profiles discussed herein. According to ESM, the energetic span (δE) is defined as:

$$\begin{bmatrix} \delta\mathbf{E} = T_{TDTS} - I_{TDI} \text{ if TDTS appears after TDI} \\ \delta\mathbf{E} = T_{TDTS} - I_{TDI} + \Delta G_r \text{ if TDTS appears before TDI} \end{bmatrix}$$

Where TDTS and TDI are transition state and intermediate state that maximize the energetic span.

3. Results

3.1 Monomer Characterization

Characterization of (-)-Menthide

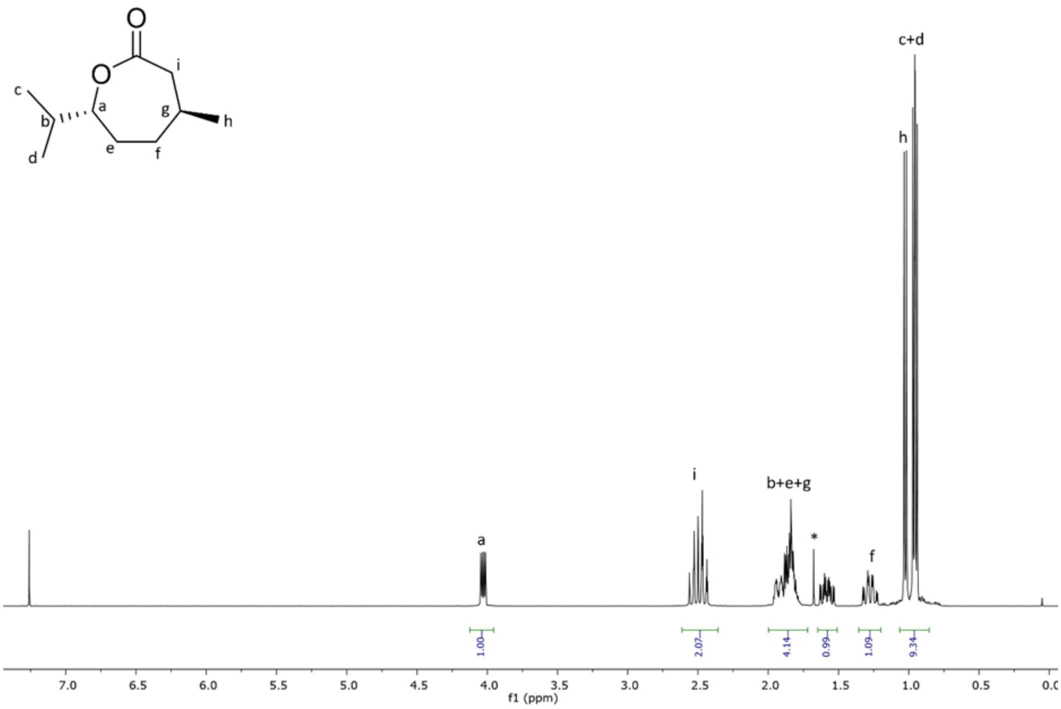

Figure S2: ^{1}H NMR spectrum of (-)-menthide in $CDCl_3$ (400 MHz, 298 K; * impurity in used $CDCl_3$). Assigned based on the COSY NMR spectrum in Figure S4.

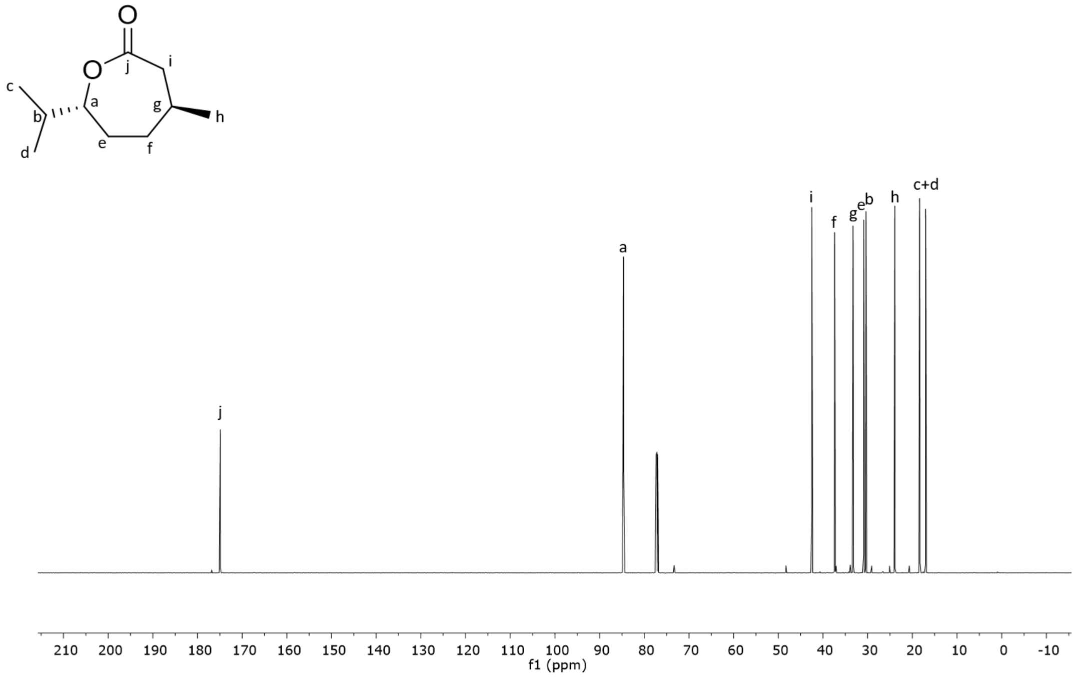

Figure S3: ^{13}C NMR spectrum of (-)-menthide in $CDCl_3$ (176 MHz, 298 K). Assigned based on the HSQC NMR spectrum in Figure S5.

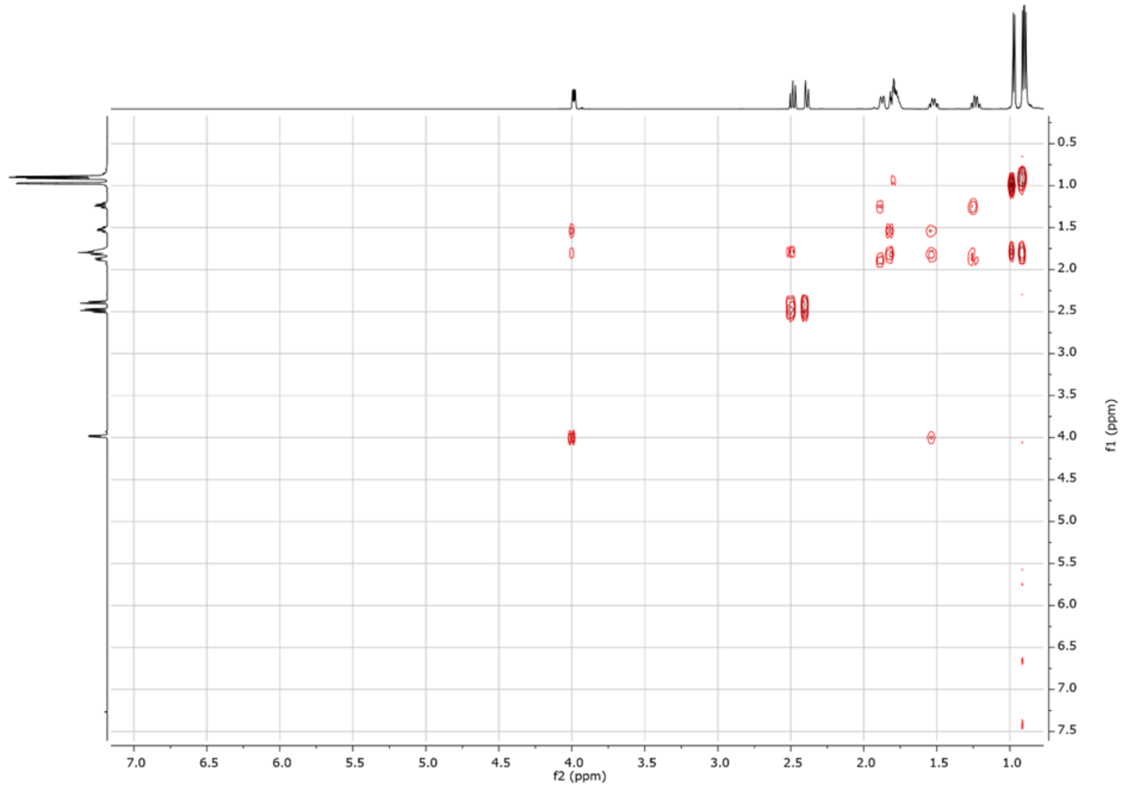

Figure S4: COSY NMR spectrum of (-)-menthide in $CDCl_3$ (700 MHz, 298 K).

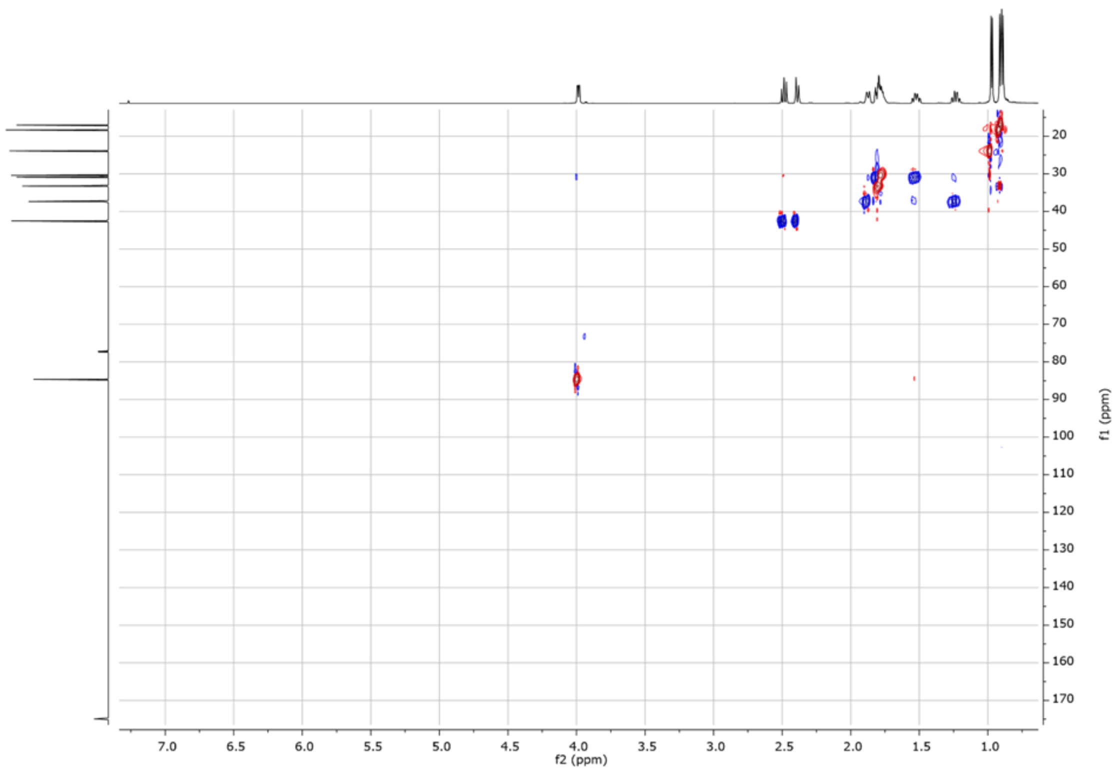

Figure S5: HSQC NMR spectrum of (-)-menthide in $CDCl_3$ (700 MHz, 176 MHz, 298 K).

ESI-MS: m/z = 171.14 ($[M+H]^+$: $C_{10}H_{19}O_2^+$)

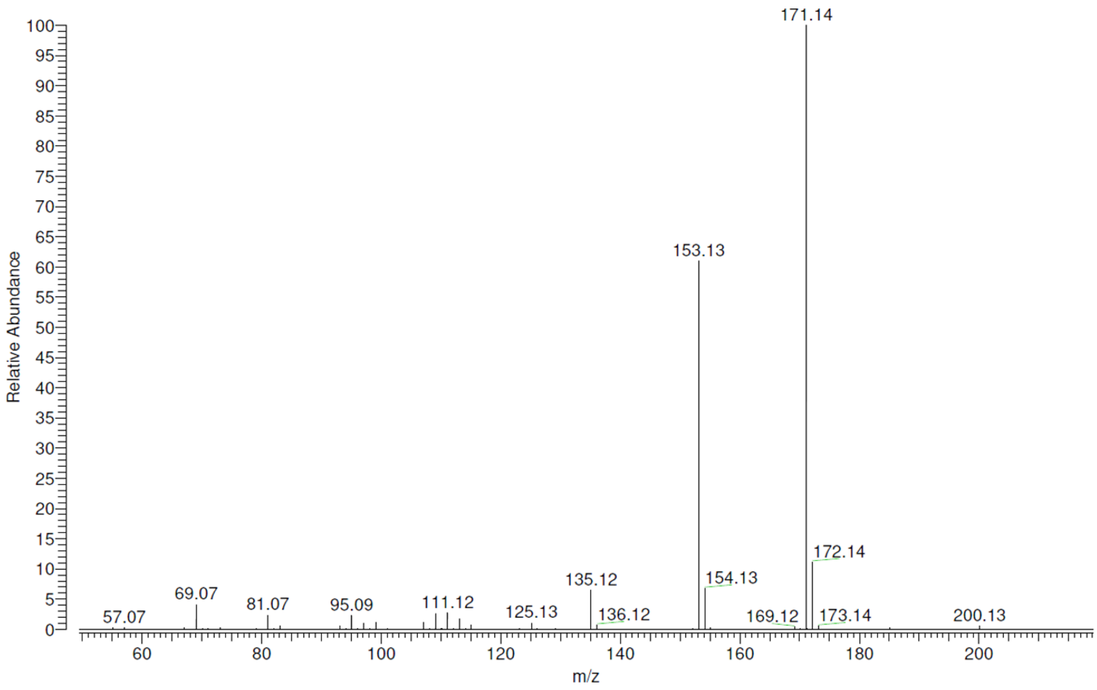

Figure S6: Mass spectrum of ESI-MS analysis of (-)-menthide.

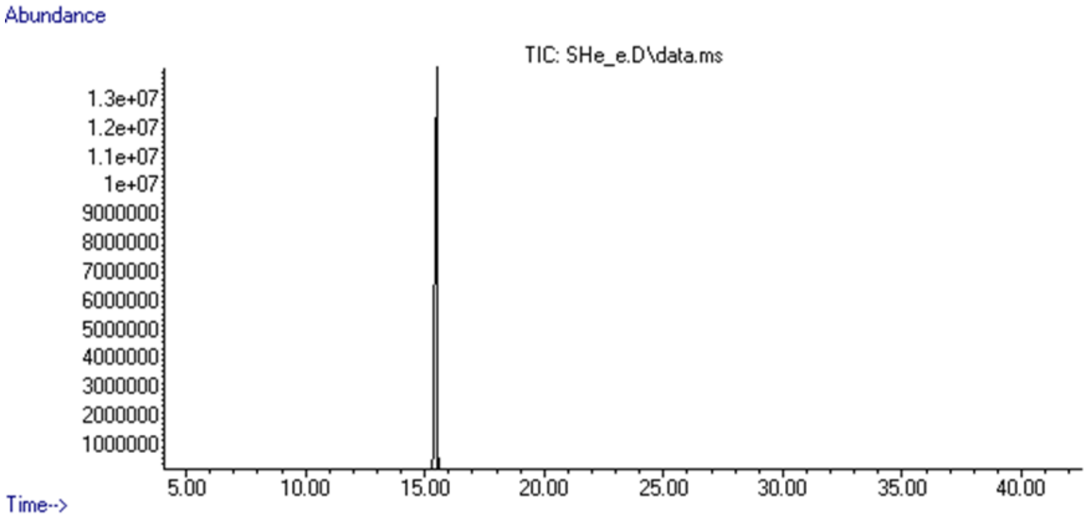

Figure S7: Chromatogram of abundance over time for the GC-MS measurement of (-)-menthide in acetone.

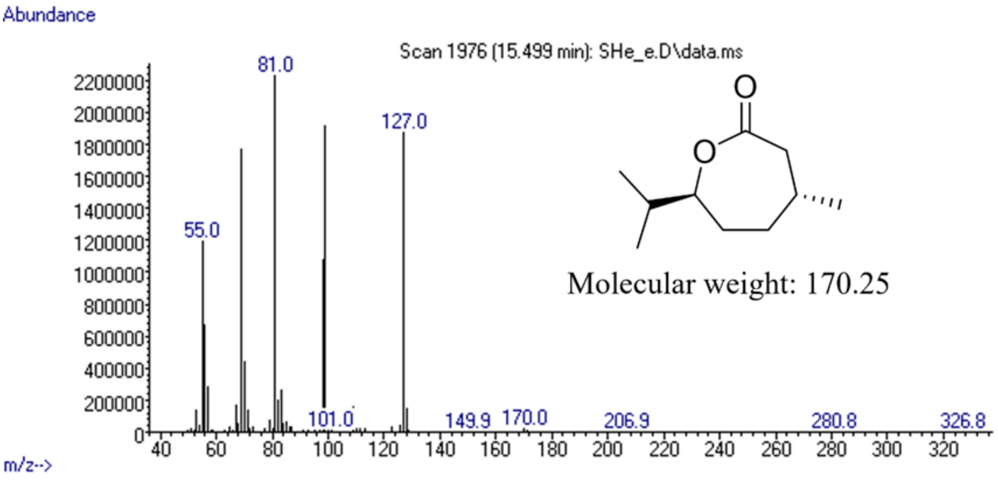

Figure S8: Mass spectrum of the (-)-menthide analysis.

Characterization of (+)-Dihydrocarvide

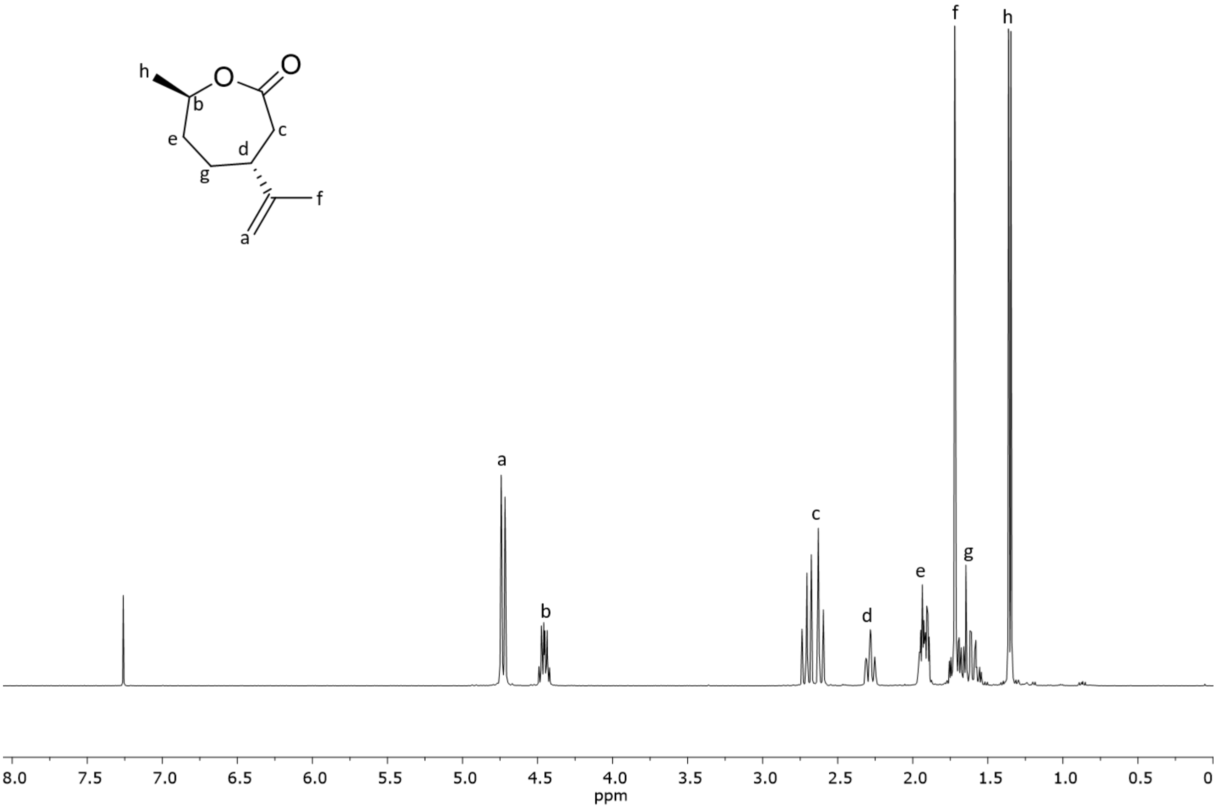

Figure S9: ^{1}H NMR spectrum of (+)-dihydrocarvide in $CDCl_3$ (400 MHz, 298 K).

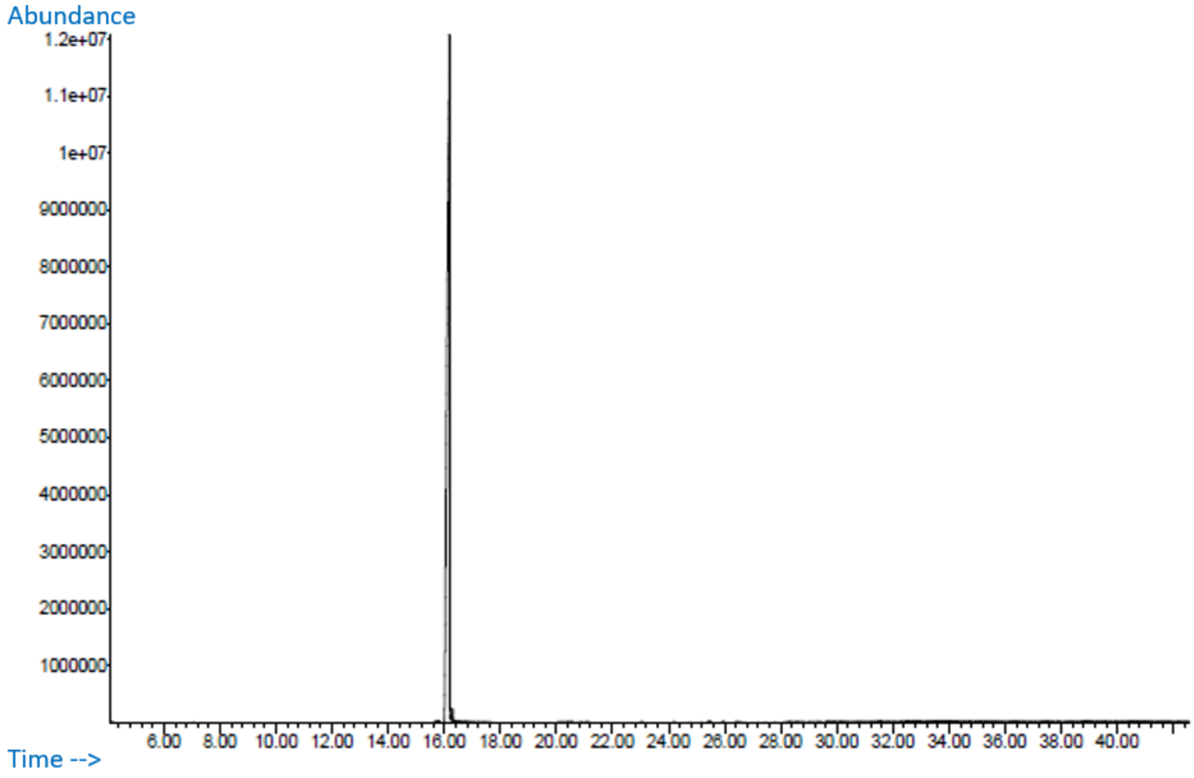

Figure S10: Chromatogram of abundance over time for the GC-MS measurement of (+)-dihydrocarvide.

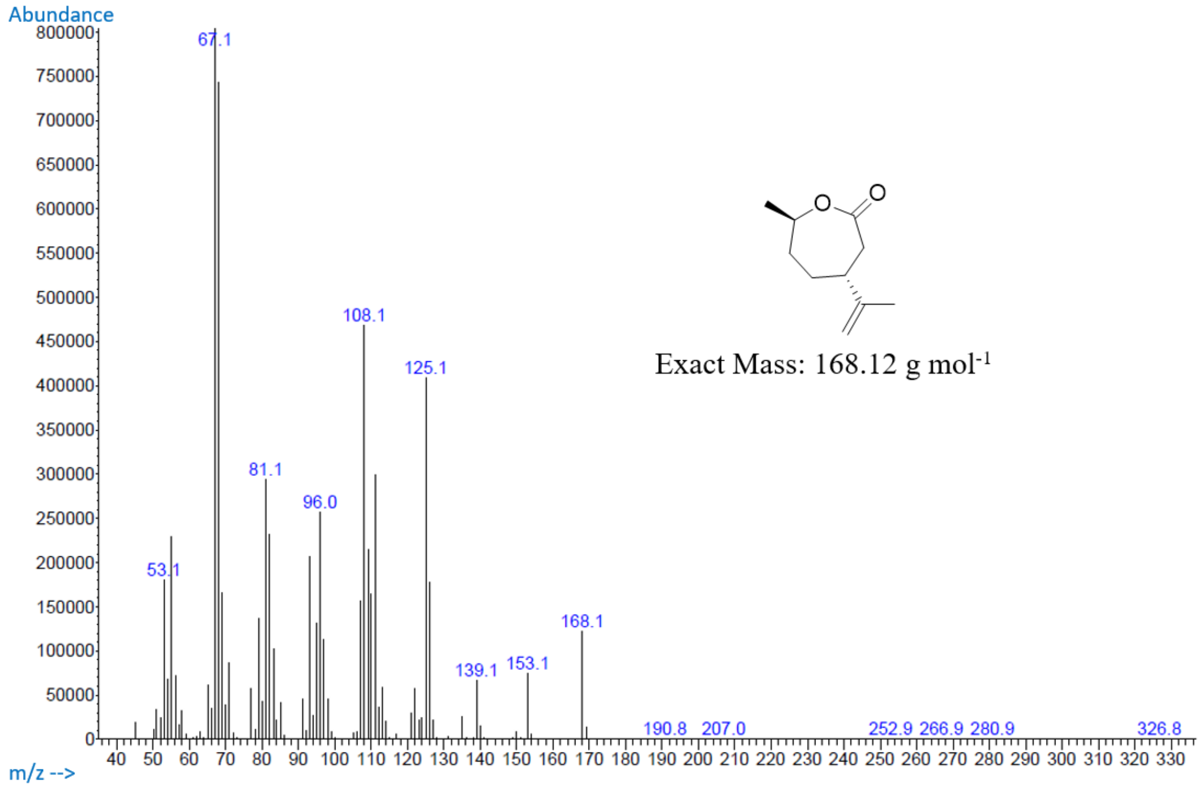

Figure S11: Mass spectrum of the (+)-dihydrocarvide GC-MS analysis.

Characterization of (+)-Carvomenthide

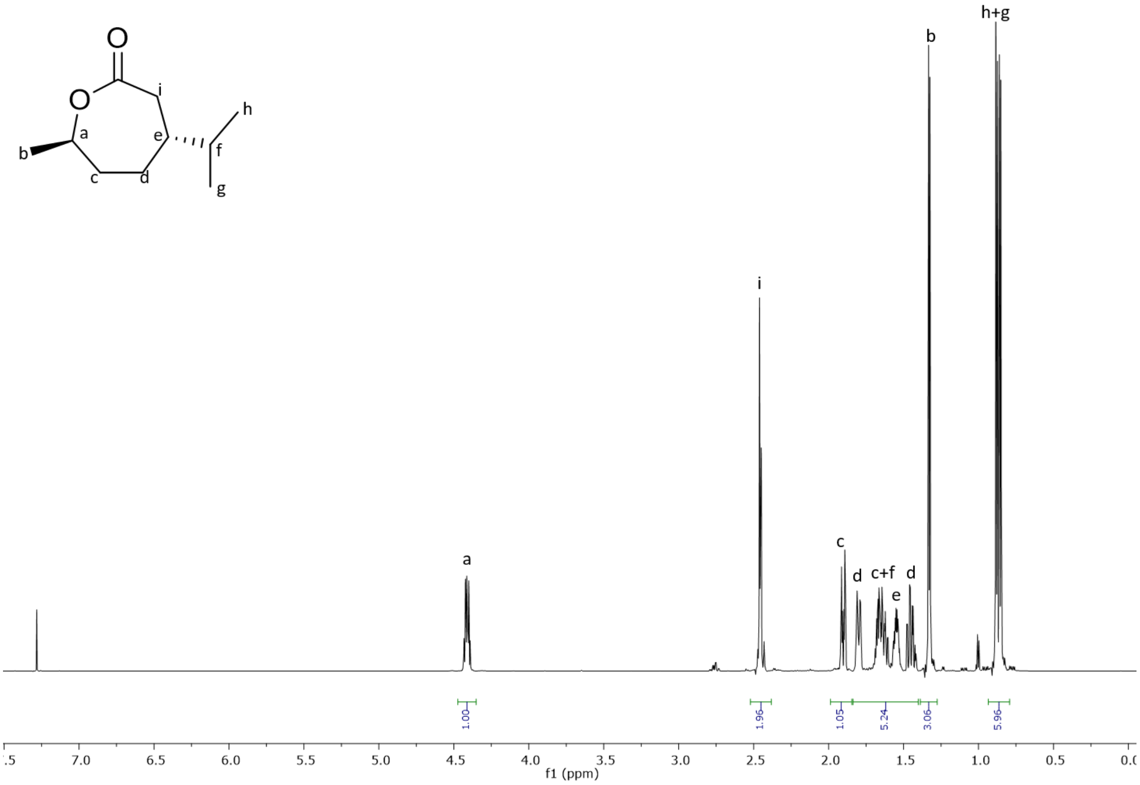

Figure S12: ^{1}H NMR spectrum of (+)-carvomenthide in $CDCl_3$ (400 MHz, 298 K). Assigned based on the COSY NMR spectrum in Figure S14.

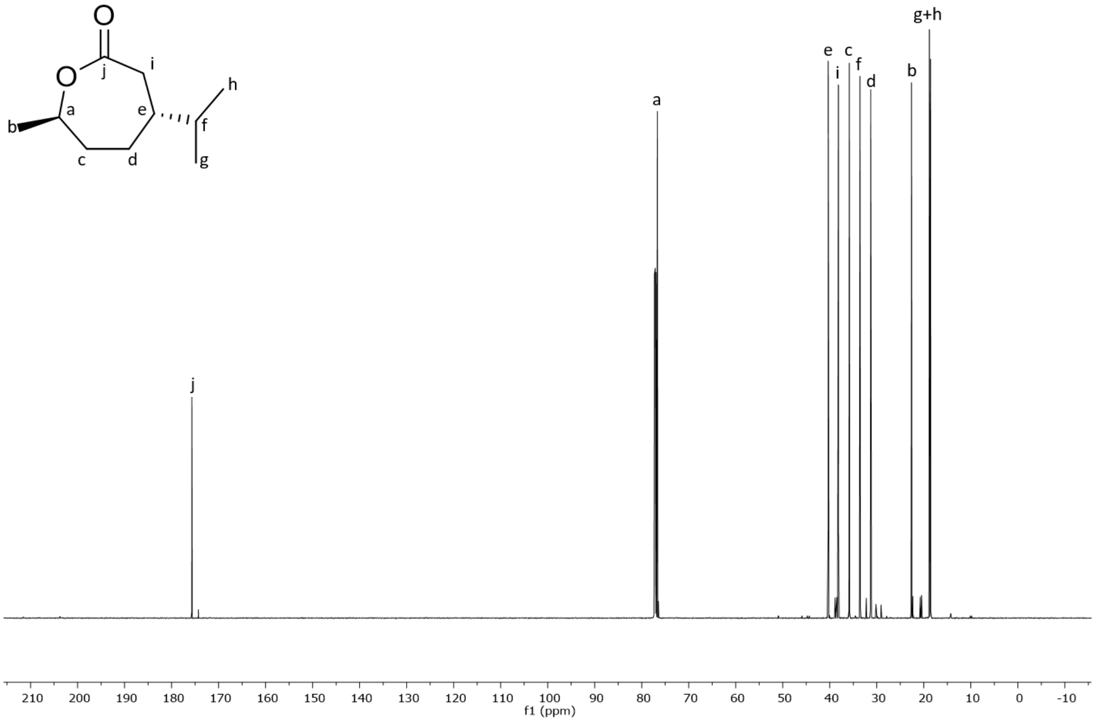

Figure S13: ^{13}C NMR spectrum of (+)-carvomenthide in $CDCl_3$ (176 MHz, 298 K). Assigned based on the HSQC spectrum in Figure S15.

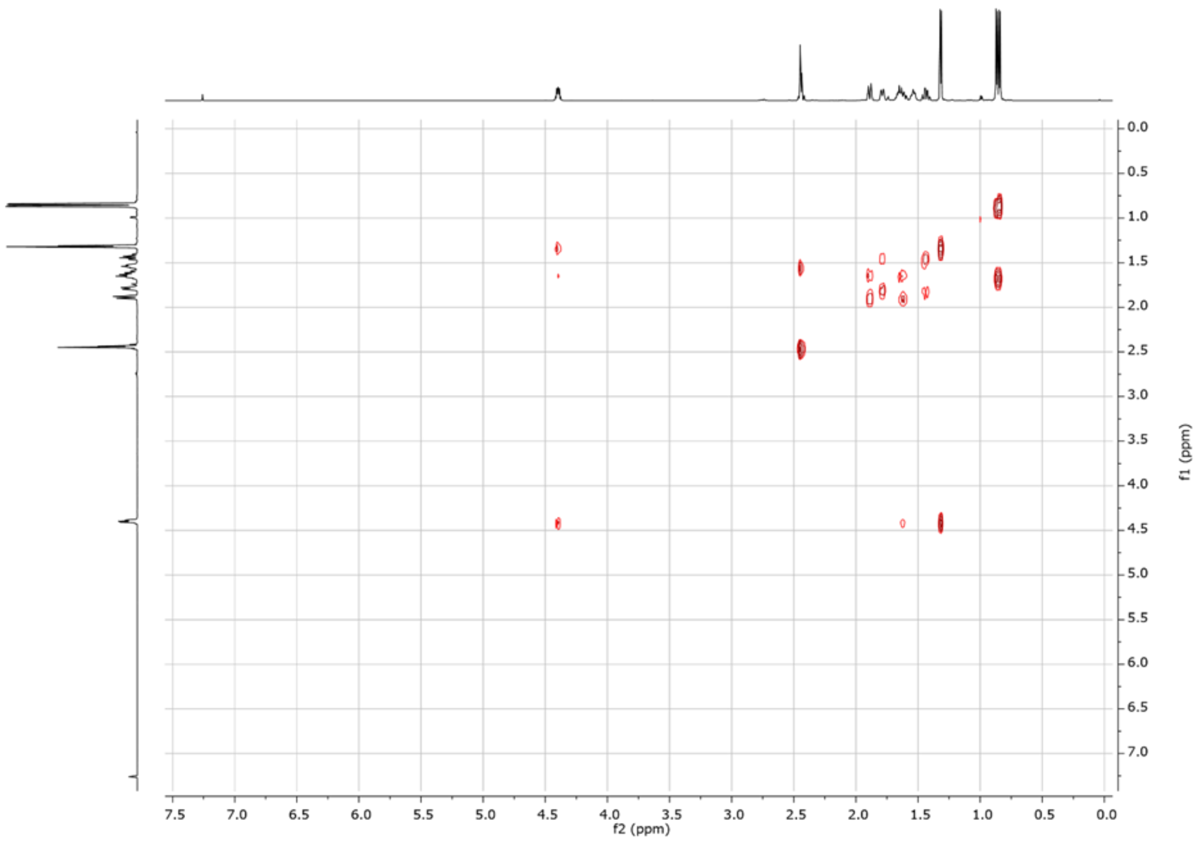

Figure S14: COSY NMR spectrum of (+)-carvomenthide in $CDCl_3$ (700 MHz, 298 K).

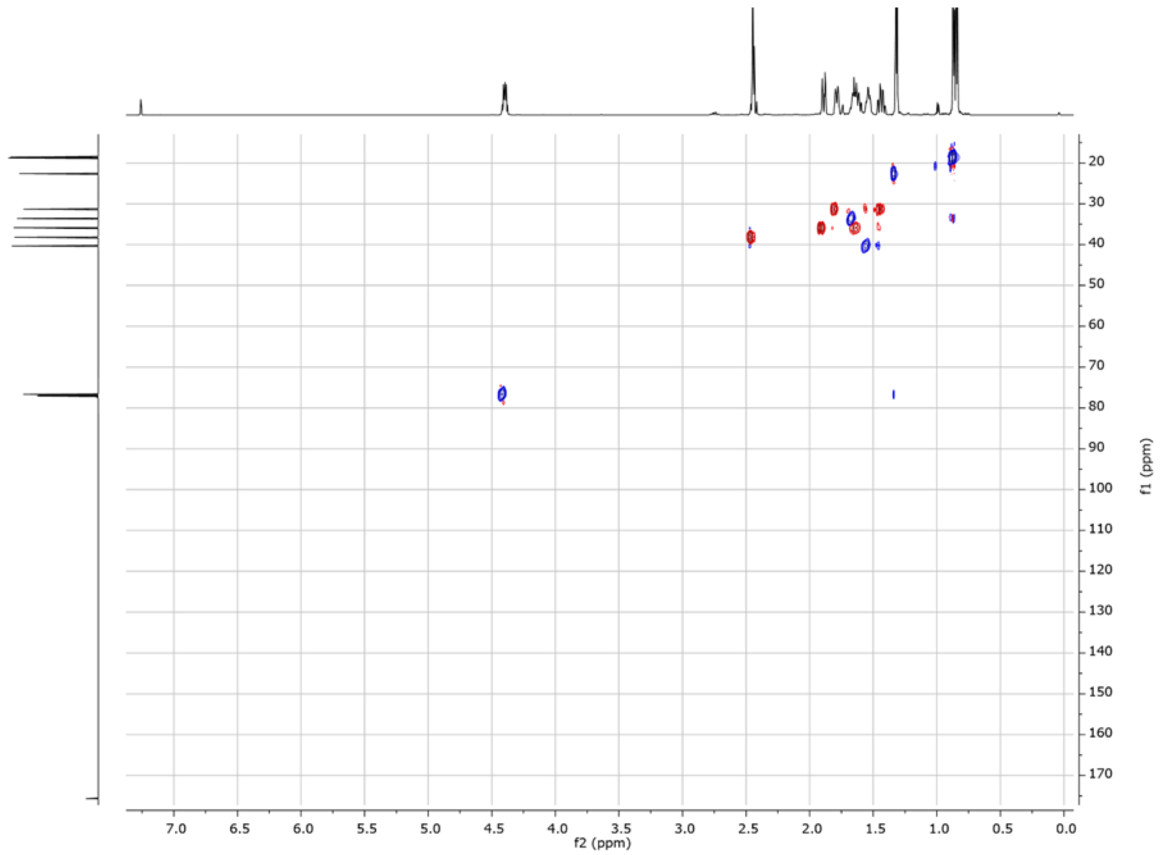

Figure S15: HSQC NMR spectrum of (+)-carvomenthide in $CDCl_3$ (700 MHz, 176 MHz, 298 K).

ESI-MS: m/z = 171.1 ([M+H]$^+$: $C_{10}H_{19}O_2^+$)

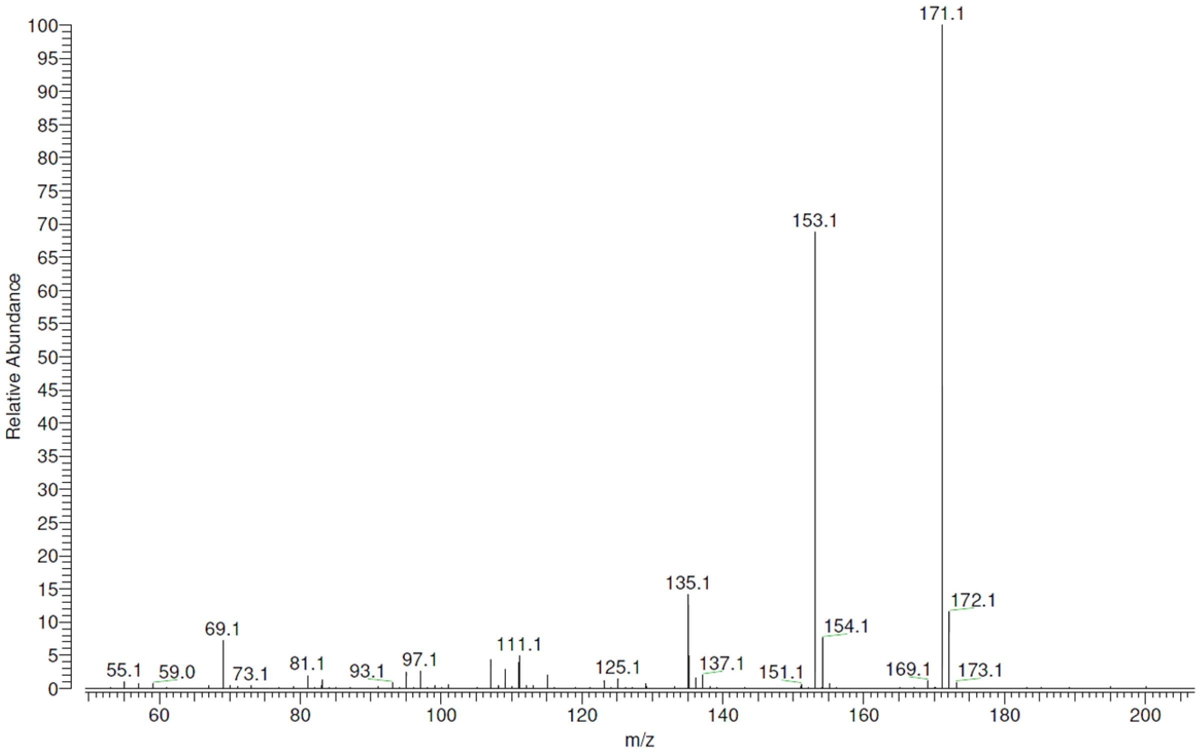

Figure S16: ESI-MS analysis of (+)-carvomenthide.

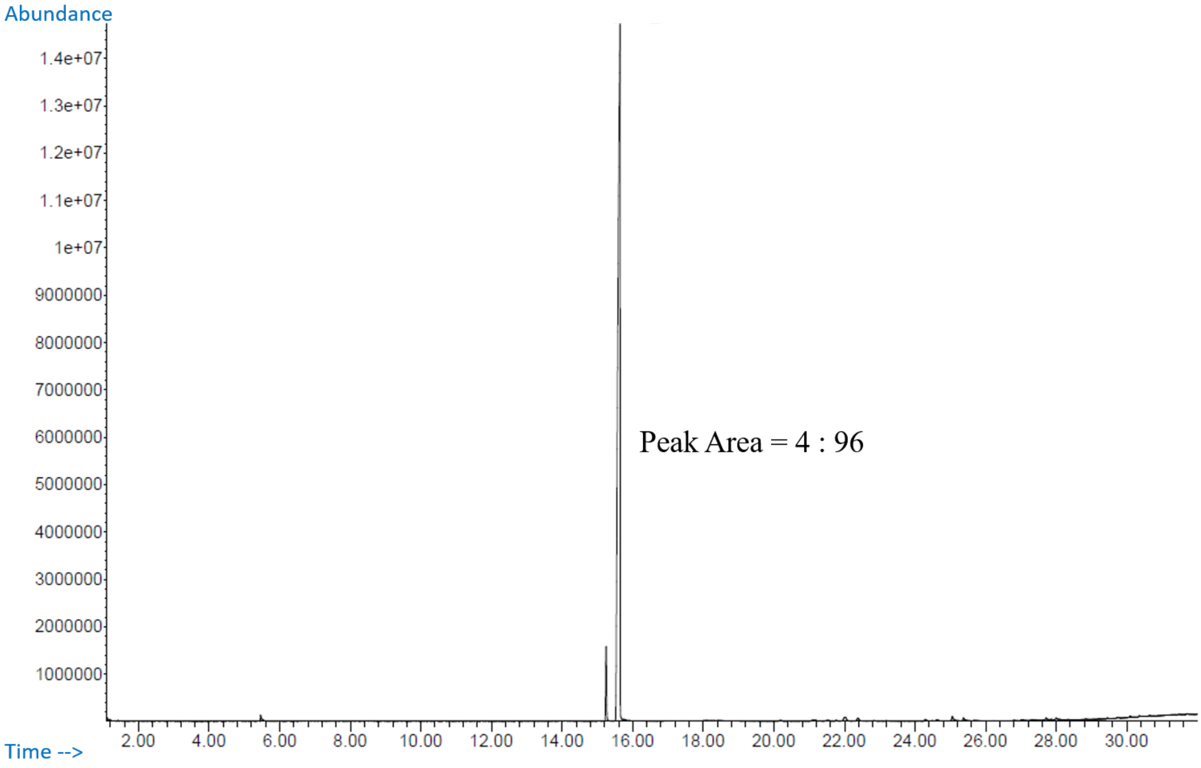

Figure S17: Chromatogram of abundance over time for the GC-MS measurement of (+)-carvomenthide.

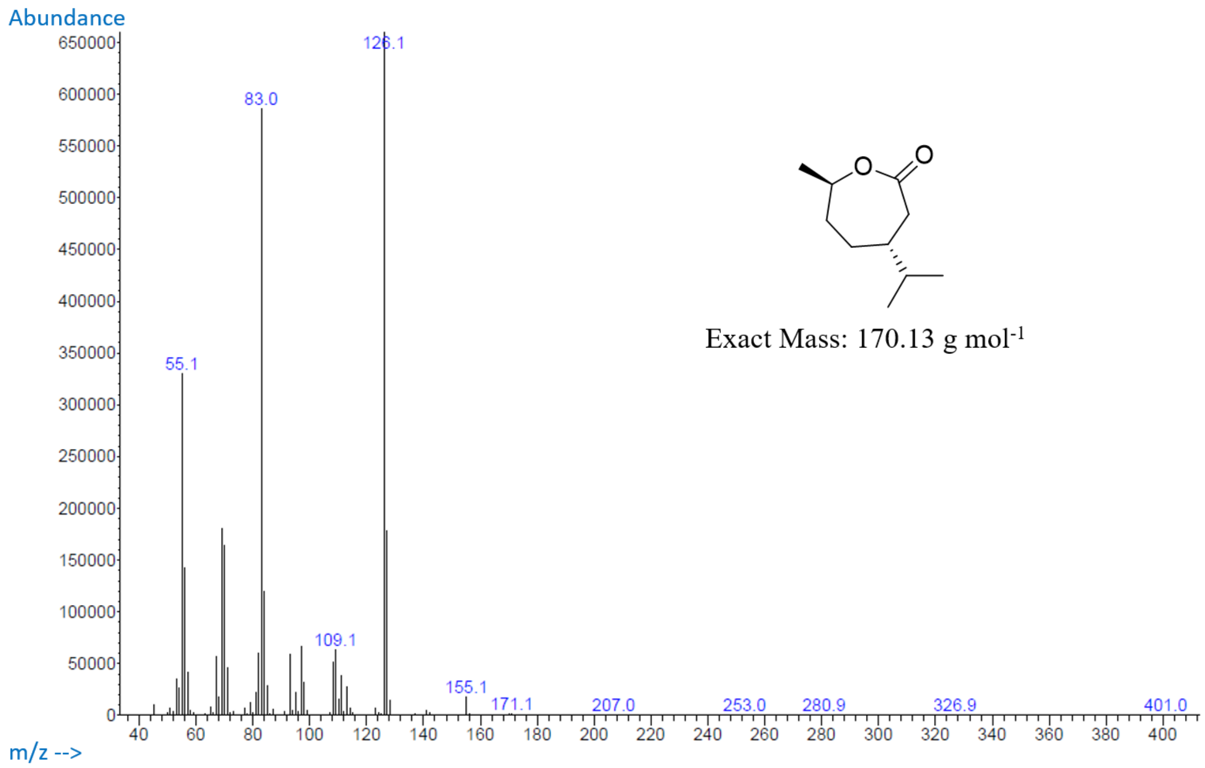

Figure S18: Mass spectrum of the GC-MS analysis of (+)-carvomenthide.

3.2 Polymer Characterization

Characterization of Poly((-)-menthide)

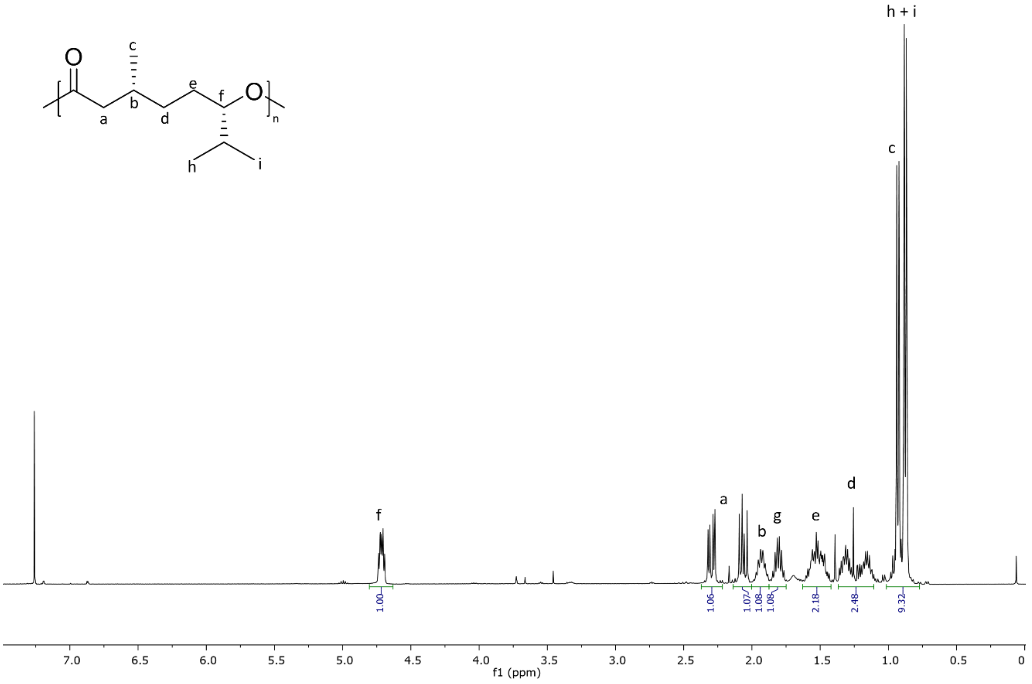

Figure S19: ^{1}H NMR spectrum of poly((-)-menthide) in $CDCl_3$ (Table 1, Entry 5; 400 MHz, 298 K).

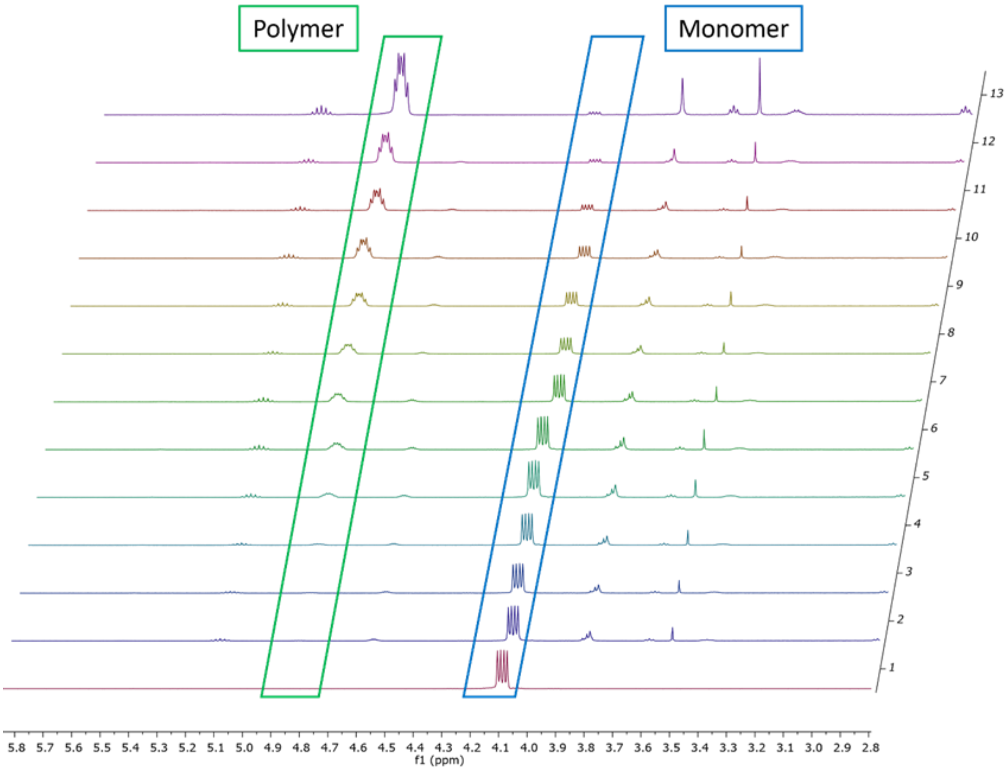

Figure S20: ^{1}H NMR spectra to illustrate the change in peaks during the polymerization of (-)-menthide with 25 equivalents of monomer at 40 °C (Table 1, Entry 6).

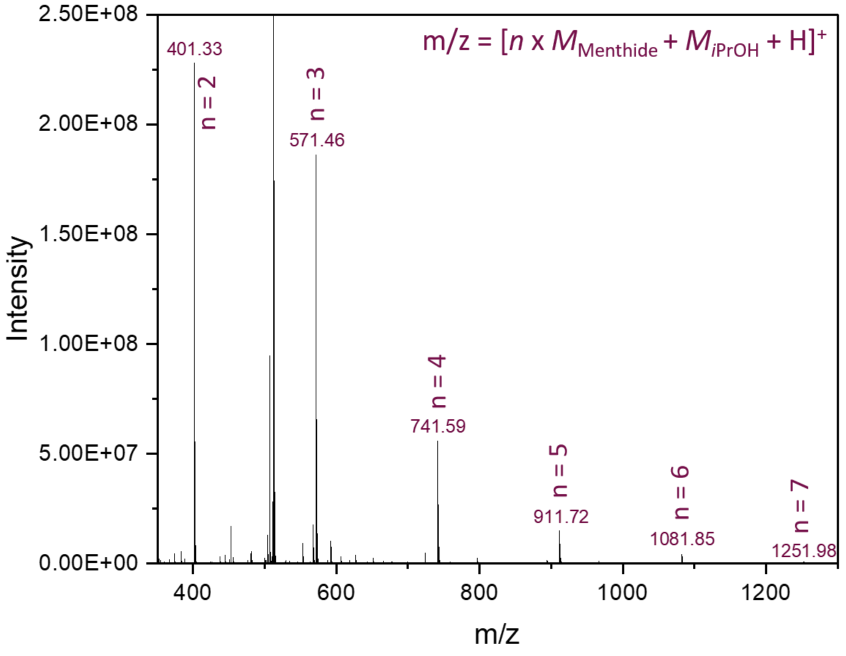

Figure S21: APCI-MS measurement in acetonitrile with [(ONOO)^{tBu}Y(iPrO)(THF)] and (-)-menthide (4.98 µmol of catalyst, 24.9 µmol of monomer, 0.5 mL toluene, 60 °C).

Characterization of Poly((+)-carvomenthide)

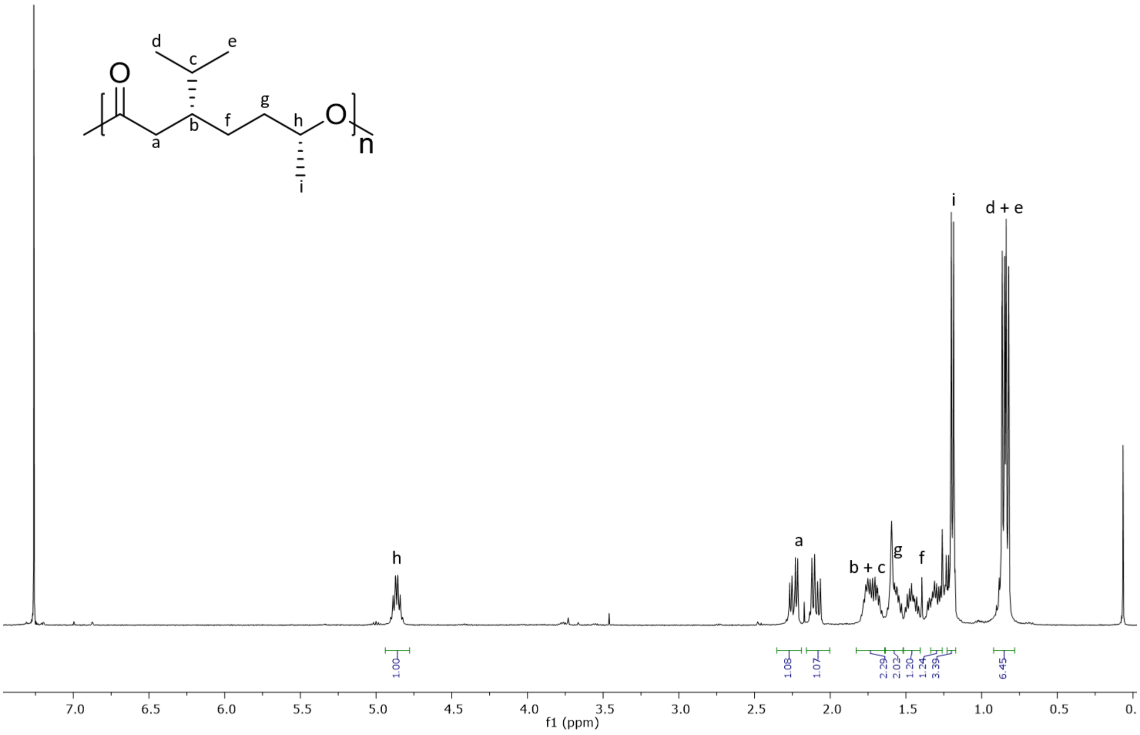

Figure S22: ^{1}H NMR spectrum of poly((+)-carvomenthide) in $CDCl_3$ (Table 1, Entry 12; 400 MHz, 298 K).

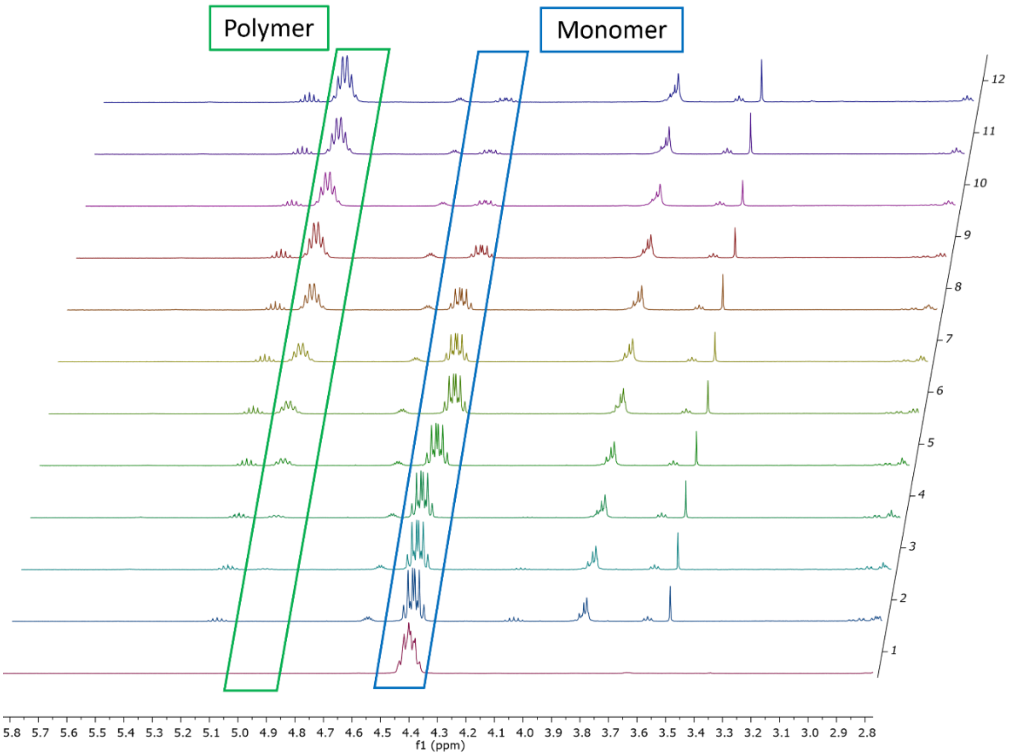

Figure S23: ^{1}H NMR spectra to illustrate the change in peaks during the polymerization of (+)-carvomenthide with 25 equivalents of monomer at 40 °C (Table 1, Entry 9).

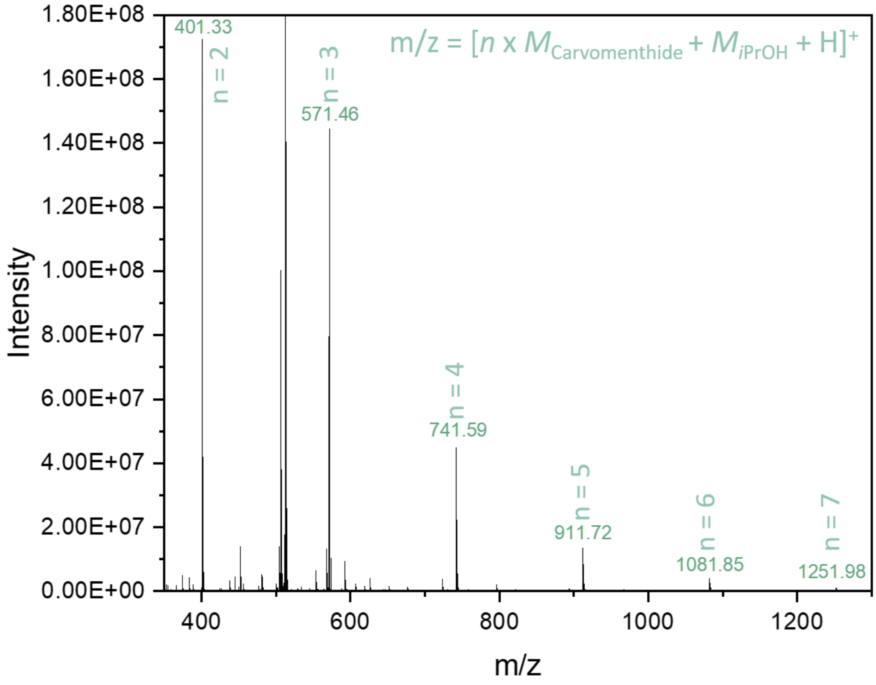

Figure S24: APCI-MS measurement in acetonitrile with [(ONOO)^{tBu}Y(iPrO)(THF)] and (+)-carvomenthide (4.98 µmol of catalyst, 24.9 µmol of monomer, 0.5 mL toluene, 60 °C).

3.3 Polymerization Studies

(-)-Menthide

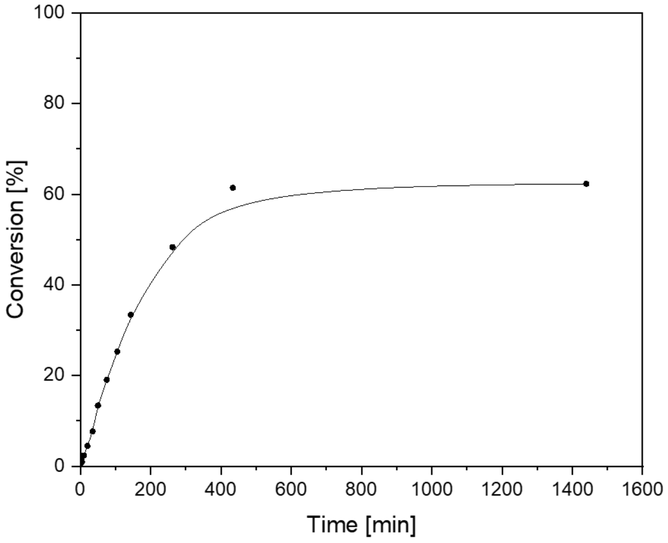

Figure S25: Conversion over reaction time plot for the ROP of (-)-menthide with 25 equivalents of monomer at 22 °C in toluene (Table 1, Entry 7).

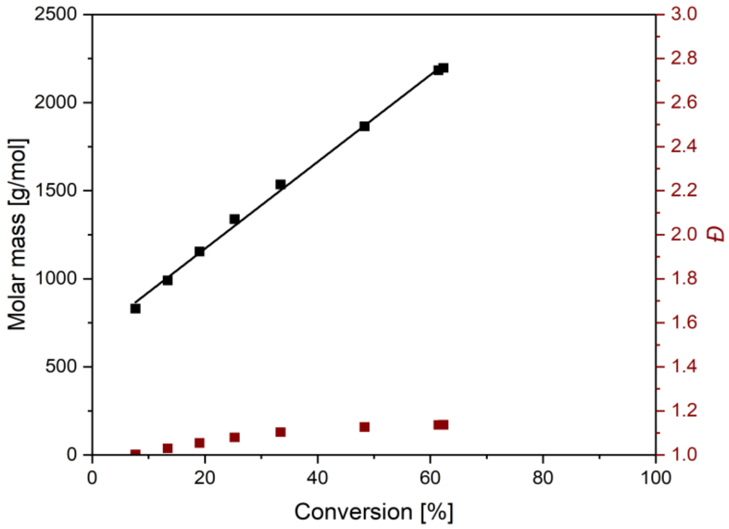

Figure S26: Molar mass and polydispersity vs. the conversion for the ROP of 25 equivalents of (-)-menthide at 22 °C (Table 1, Entry 7). *The molar masses were measured using the same SEC system, but with a previous column set, leading to values that differ from those in the main text (Table 1) by approximately a factor of three. However, since the measurements were performed relative to polystyrene standards, the relative values remain comparable, preserving the linearity of the growth.

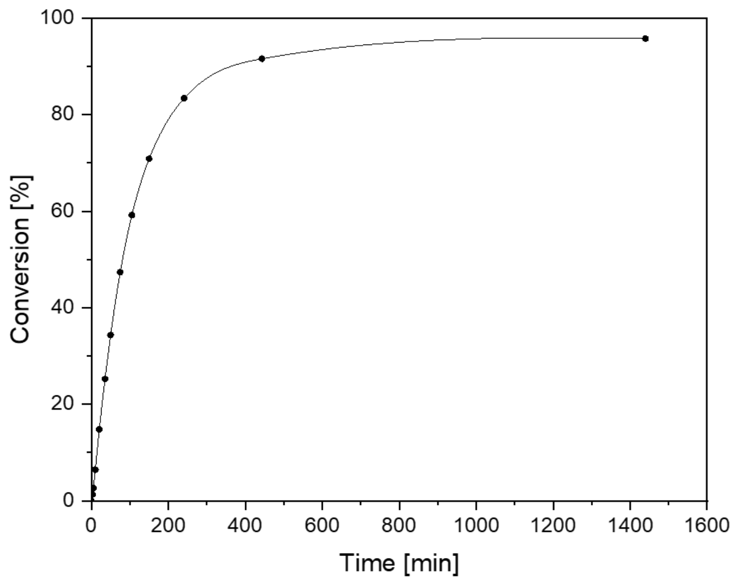

Figure S27: Conversion over reaction time plot for the ROP of (-)-menthide with 25 equivalents of monomer at 40 °C in toluene (Table 1, Entry 6).

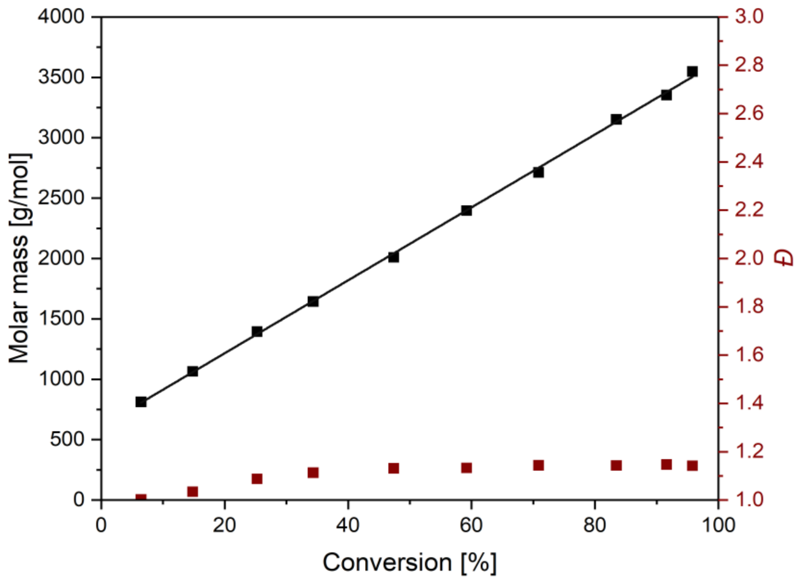

Figure S28: Molar mass and polydispersity plotted over the conversion for the ROP of 25 equivalents of (-)-menthide at 40 °C (Table 1, Entry 6). *The molar masses were measured using the same SEC system, but with a previous column set, leading to values that differ from those in the main text (Table 1) by approximately a factor of three. However, since the measurements were performed relative to polystyrene standards, the relative values remain comparable, preserving the linearity of the growth.

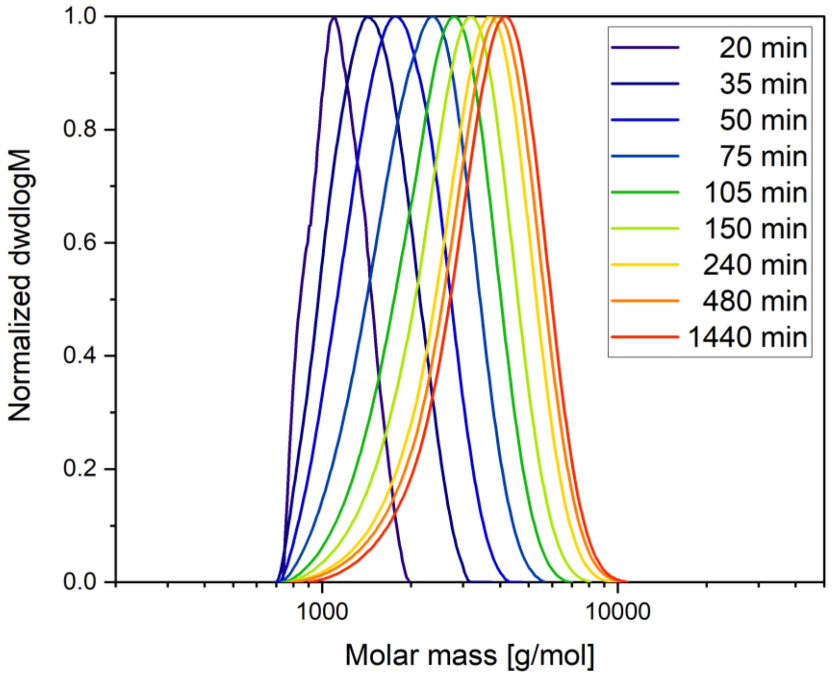

Figure S29: SEC traces of the ROP of (-)-menthide (25 eq.) at 40 °C in toluene. Molar masses are determined relative to polystyrene standards. *The molar masses were measured using the same SEC system, but with a previous column set, leading to values that differ from those in the main text (Table 1) by approximately a factor of three.

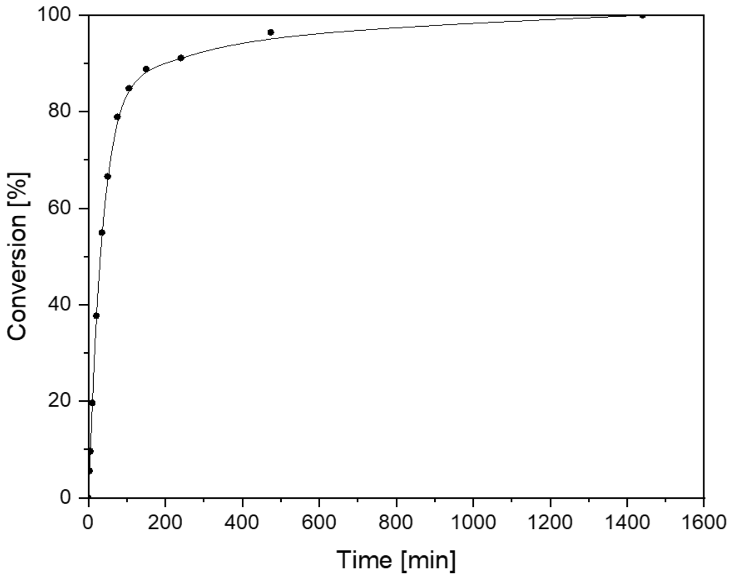

Figure S30: Conversion over reaction time plot for the ROP of (-)-menthide with 25 equivalents of monomer at 60 °C in toluene (Table 1, Entry 3).

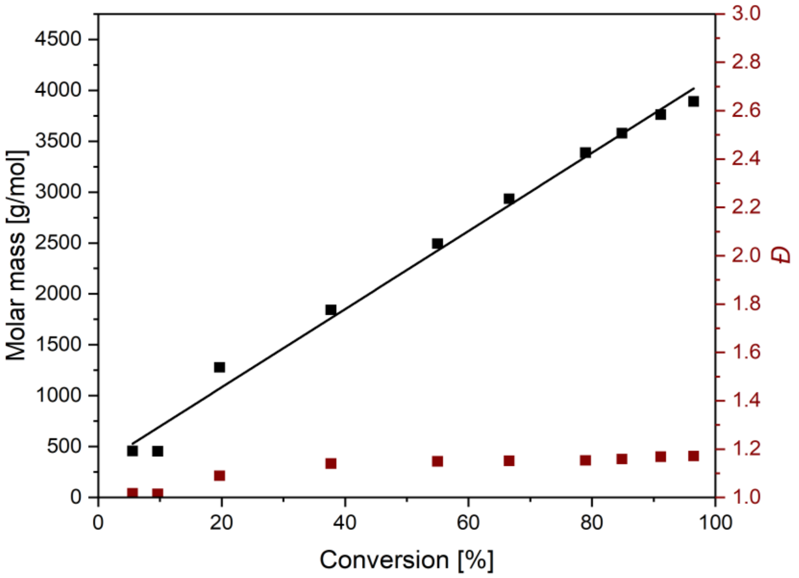

Figure S31: Molar mass and polydispersity plotted over the conversion for the ROP of 25 equivalents of (-)-menthide at 60 °C (Table 1, Entry 3). *The molar masses were measured using the same SEC system, but with a previous column set, leading to values that differ from those in the main text (Table 1) by approximately a factor of three. However, since the measurements were performed relative to polystyrene standards, the relative values remain comparable, preserving the linearity of the growth.

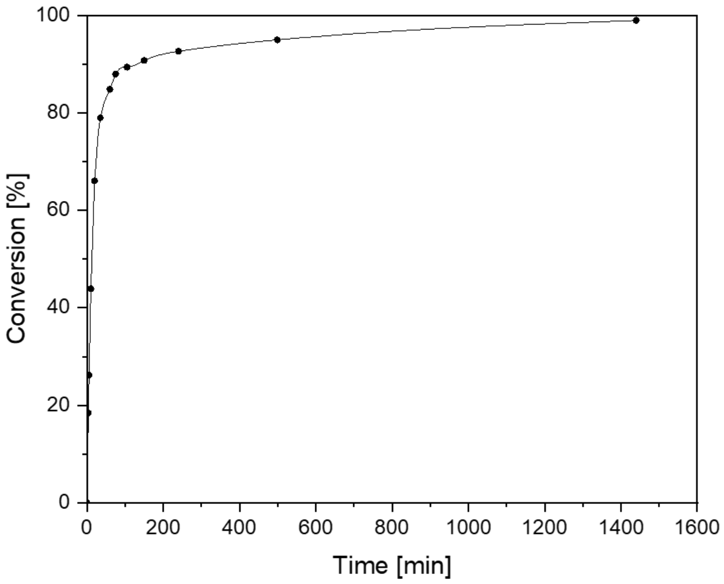

Figure S32: Conversion over reaction time plot for the ROP of (-)-menthide with 25 equivalents of monomer at 80 °C in toluene (Table 1, Entry 2).

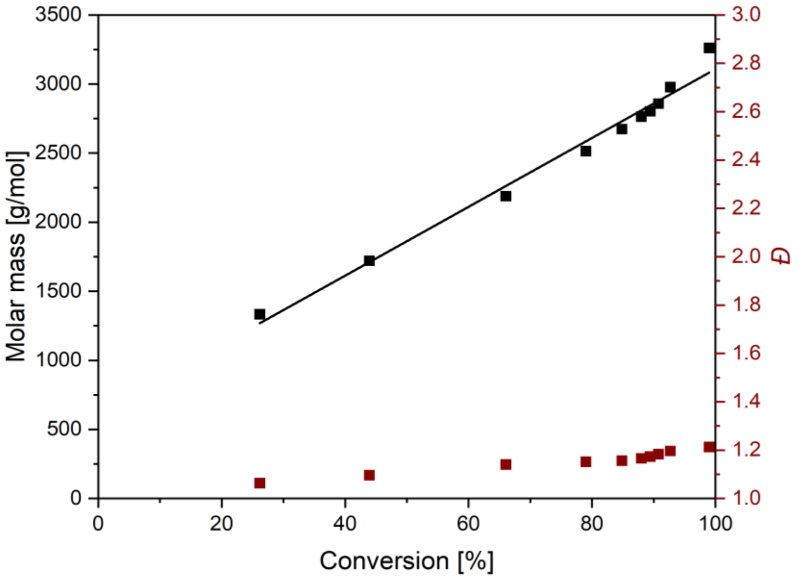

Figure S33: Molar mass and polydispersity plotted over the conversion for the ROP of 25 equivalents of (-)-menthide at 80 °C (Table 1, Entry 2). *The molar masses were measured using the same SEC system, but with a previous column set, leading to values that differ from those in the main text (Table 1) by approximately a factor of three. However, since the measurements were performed relative to polystyrene standards, the relative values remain comparable, preserving the linearity of the growth.

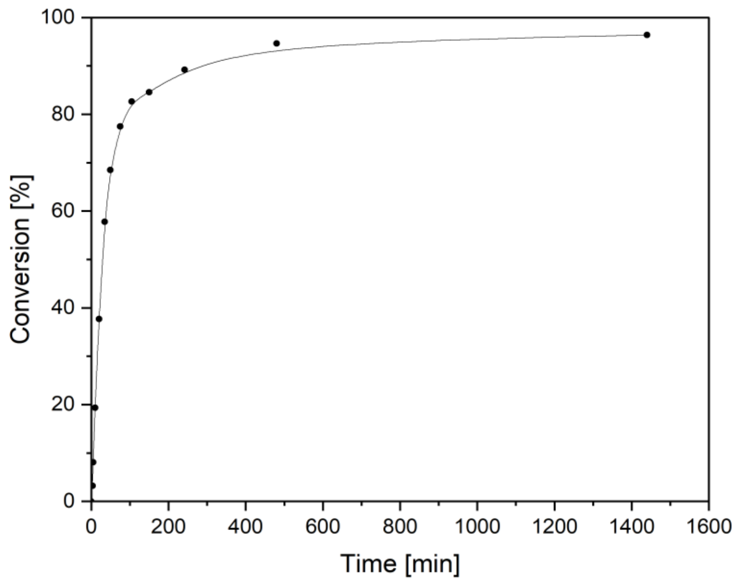

Figure S34: Conversion over reaction time plot for the ROP of (-)-menthide with the [(ONOO)^{tBu}Y(bdsa)(THF)] catalyst and 25 equivalents of monomer at 80 °C in toluene (Table S1, Entry 4).

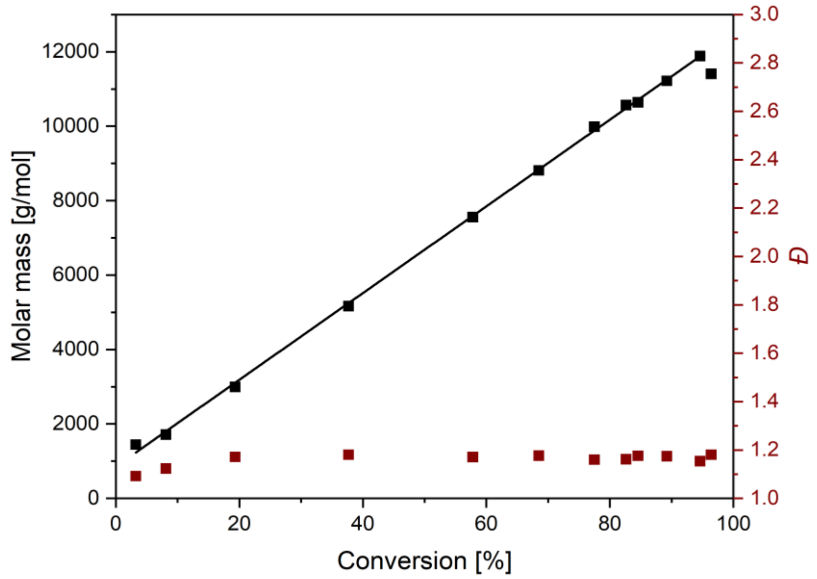

Figure S35: Molar mass and polydispersity plotted over the conversion for the ROP of 25 equivalents of (-)-menthide with the [(ONOO)^{tBu}Y(bdsa)(THF)] catalyst at 80 °C (Table S1, Entry 4).

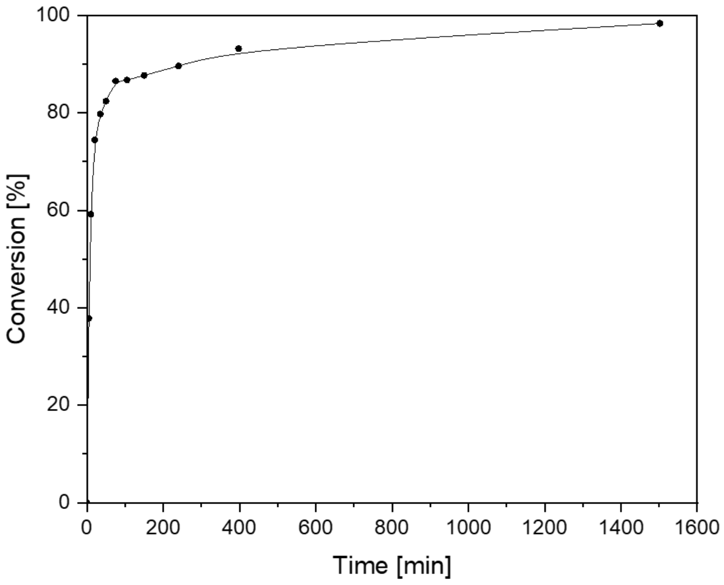

Figure S36: Conversion over reaction time plot for the ROP of (-)-menthide with 25 equivalents of monomer at 100 °C in toluene (Table 1, Entry 1).

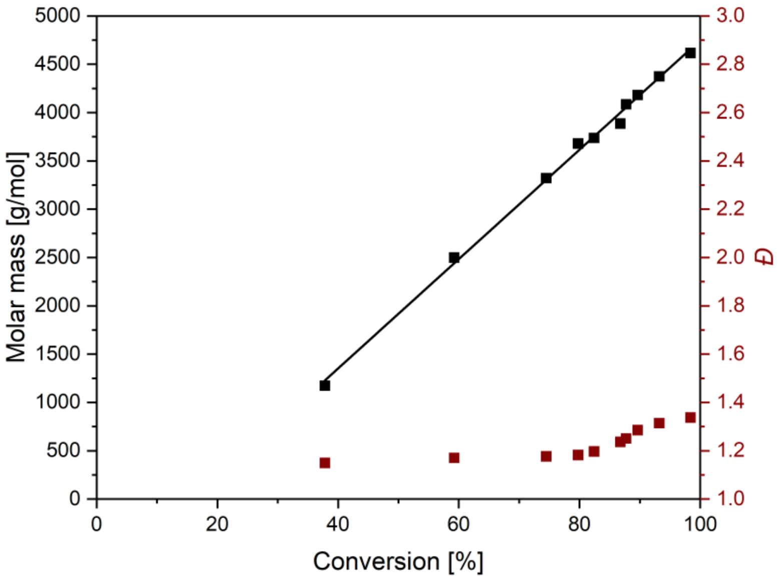

Figure S37: Molar mass and polydispersity plotted over the conversion for the ROP of 25 equivalents of (-)-menthide at 100 °C (Table 1, Entry 1). *The molar masses were measured using the same SEC system, but with a previous column set, leading to values that differ from those in the main text (Table 1) by approximately a factor of three. However, since the measurements were performed relative to polystyrene standards, the relative values remain comparable, preserving the linearity of the growth.

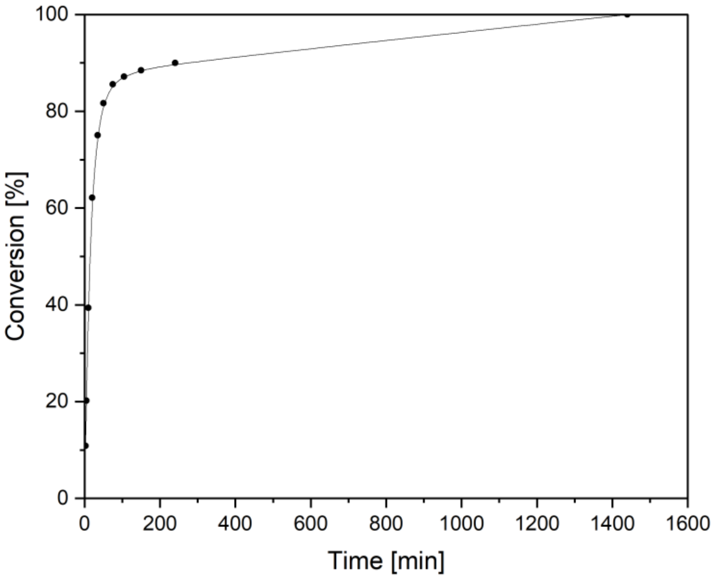

Figure S38: Conversion over reaction time plot for the ROP of (-)-menthide with the [(ONOO)^{tBu}Y(bdsa)(THF)] catalyst and 25 equivalents of monomer at 100 °C in toluene (Table S1, Entry 2).

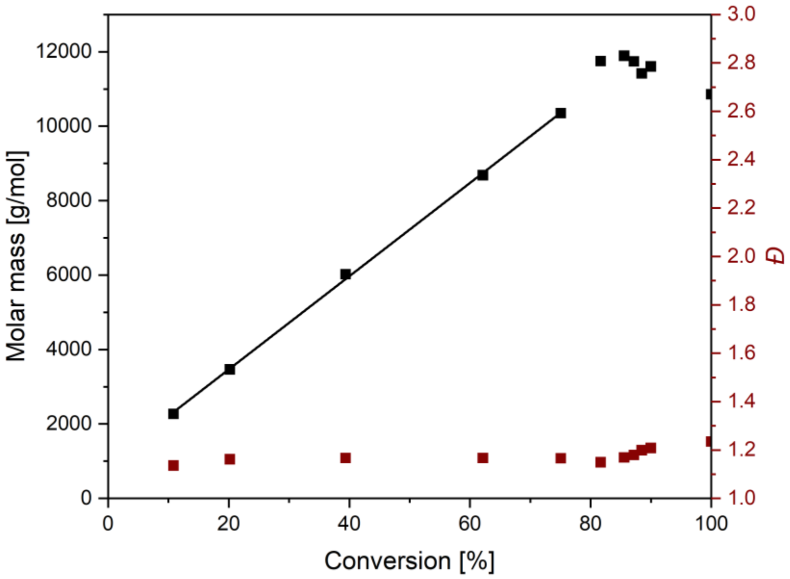

Figure S39: Molar mass and polydispersity plotted over the conversion for the ROP of 25 equivalents of (-)-menthide with the [(ONOO)^{tBu}Y(bdsa)(THF)] catalyst at 100 °C (Table S1, Entry 2).

(+)-Carvomenthide

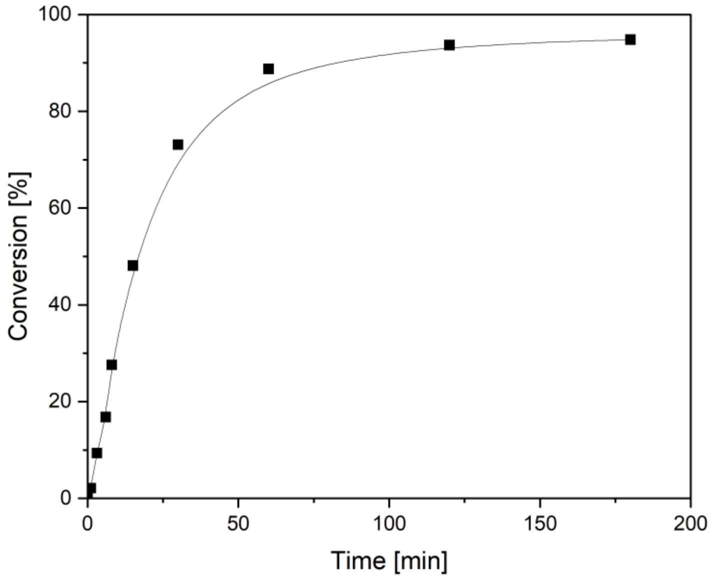

Figure S40: Conversion over reaction time plot for the ROP of (+)-carvomenthide with 25 equivalents of monomer at 21 °C in toluene (Table 1, Entry 14).

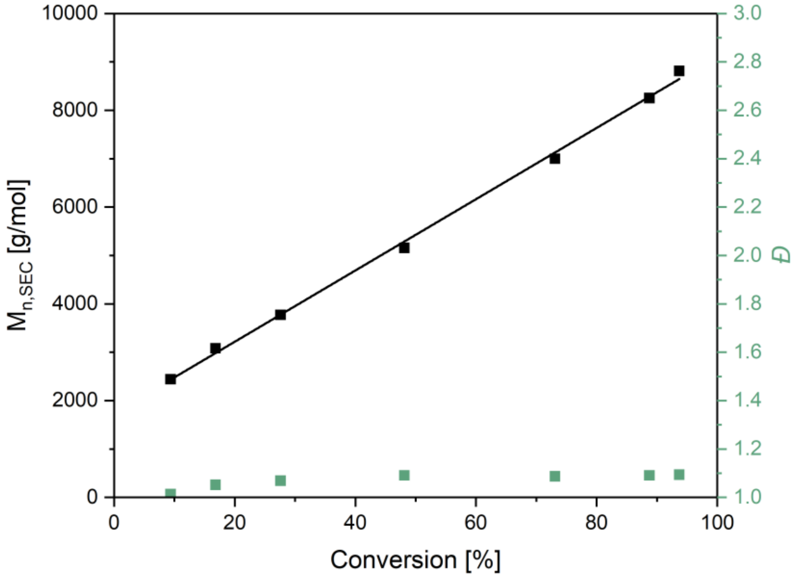

Figure S41: Molar mass and polydispersity plotted over the conversion for the ROP of 25 equivalents of (+)-carvomenthide at 21 °C (Table 1, Entry 14).

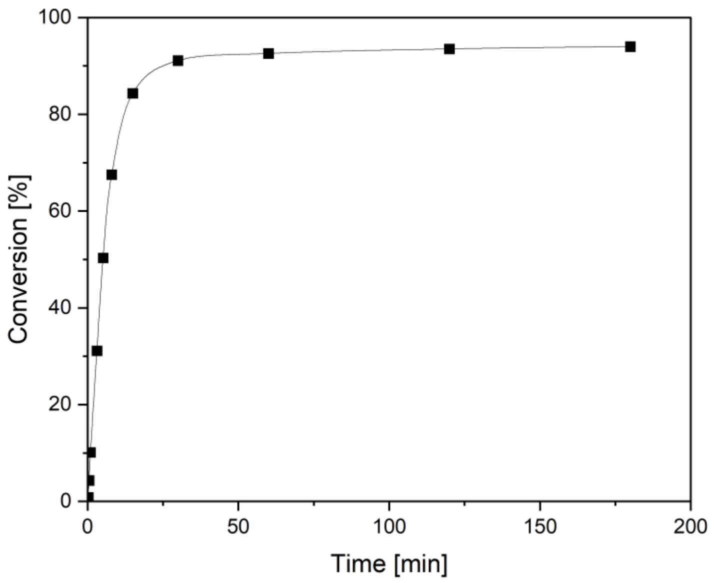

Figure S42: Conversion over reaction time plot for the ROP of (+)-carvomenthide with 25 equivalents of monomer at 40 °C in toluene (Table 1, Entry 13).

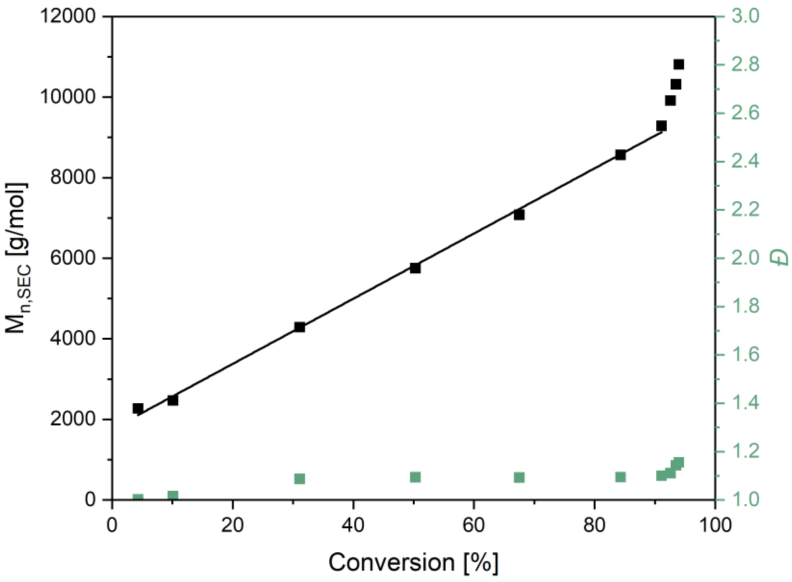

Figure S43: Molar mass and polydispersity plotted over the conversion for 25 equivalents of (+)-carvomenthide at 40 °C (Table 1, Entry 13).

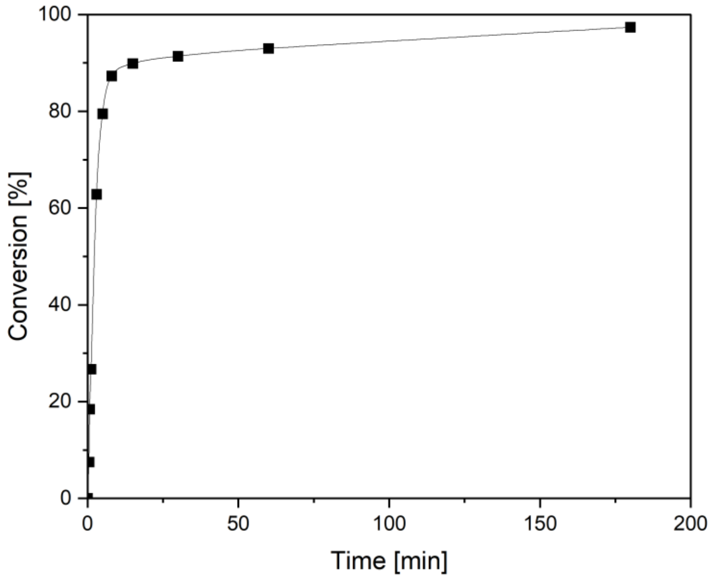

Figure S44: Conversion over reaction time plot for the ROP of (+)-carvomenthide with 25 equivalents of monomer at 60 °C in toluene (Table 1, Entry 10).

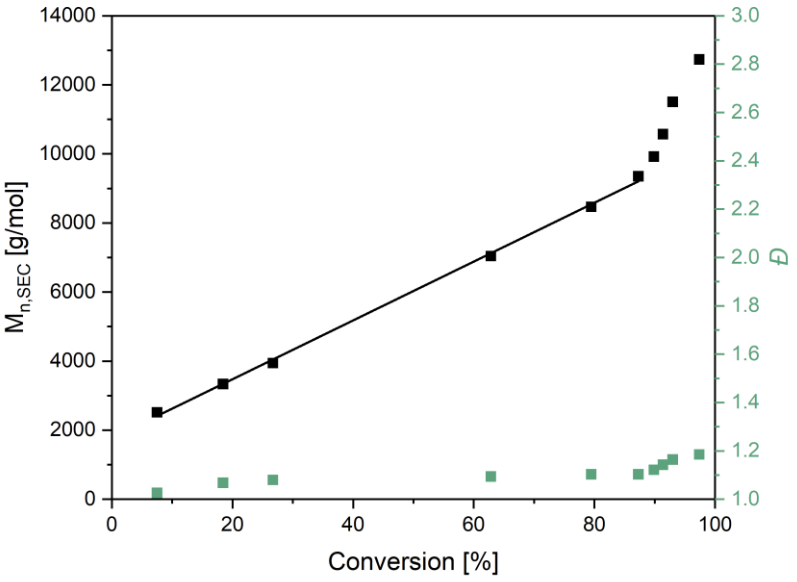

Figure S45: Molar mass and polydispersity plotted over the conversion for the ROP of 25 equivalents of (+)-carvomenthide at 60 °C (Table 1, Entry 10).

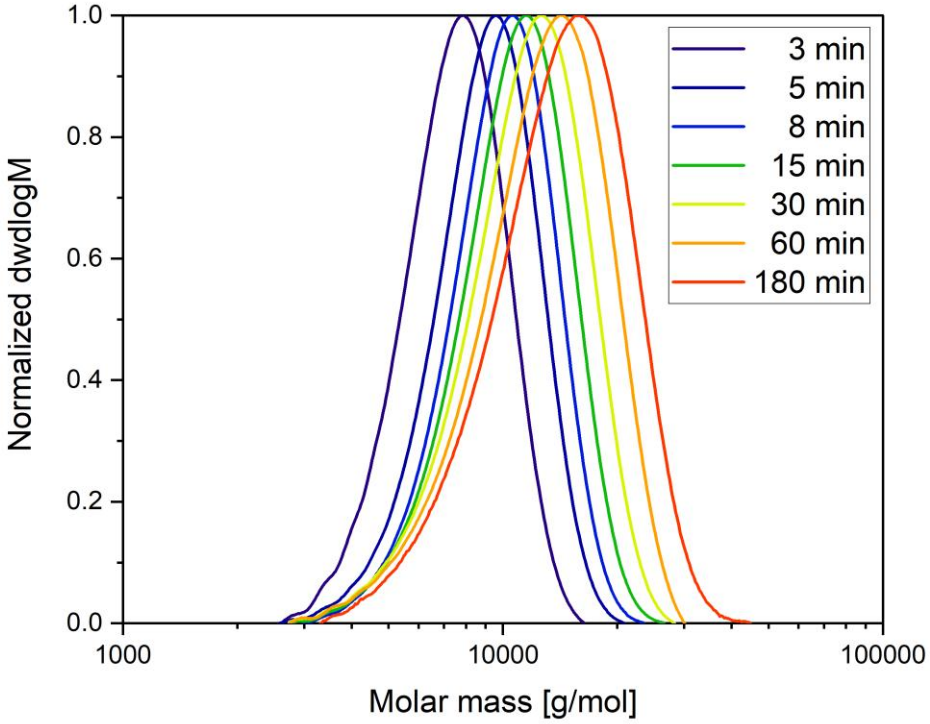

Figure S46: SEC traces of the ROP of (+)-carvomenthide (25 eq.) at 60 °C in toluene. Molar masses are measured relative to polystyrene standards.

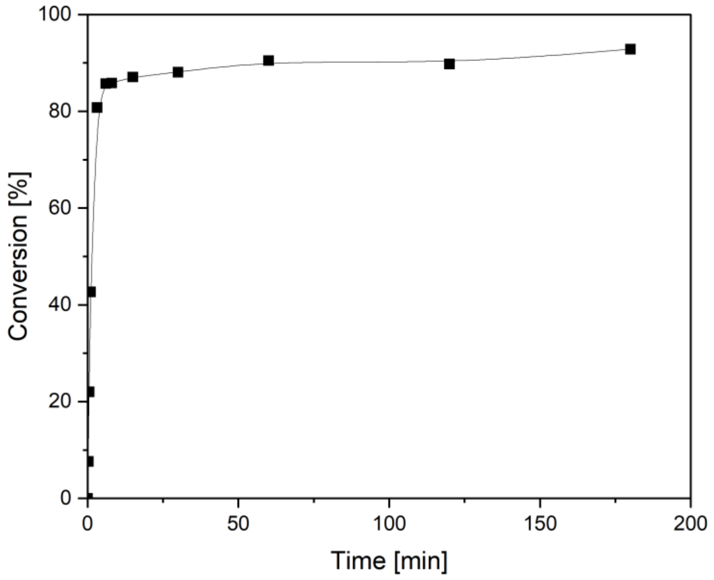

Figure S47: Conversion over reaction time plot for the ROP of (+)-carvomenthide with 25 equivalents of monomer at 80 °C in toluene (Table 1, Entry 9).

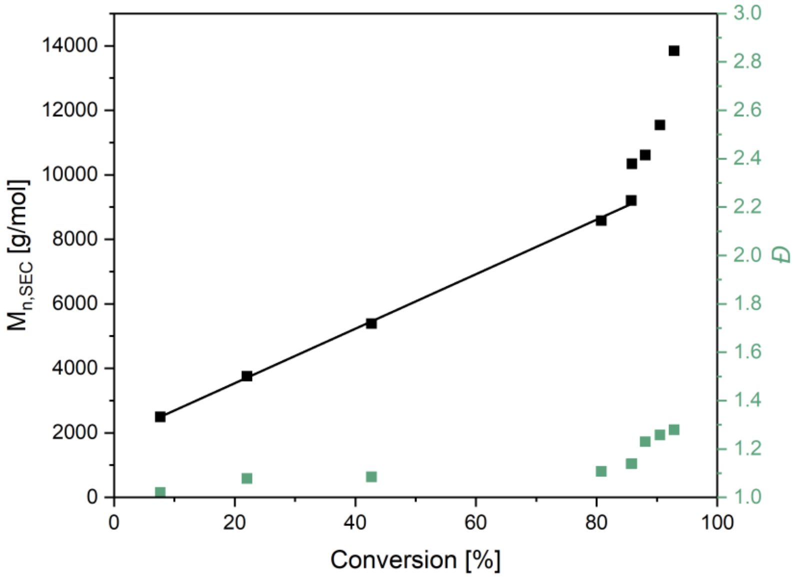

Figure S48: Molar mass and polydispersity plotted over the conversion for the ROP of 25 equivalents of (+)-carvomenthide at 80 °C (Table 1, Entry 9).

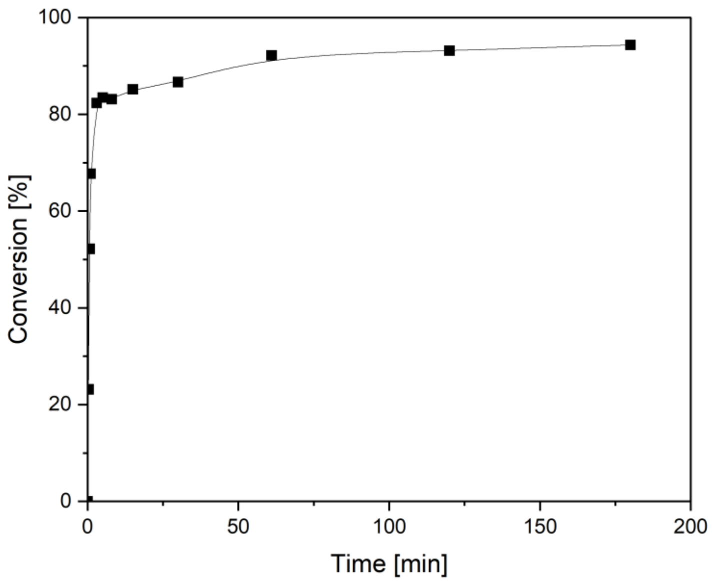

Figure S49: Conversion over reaction time plot for the ROP of (+)-carvomenthide with 25 equivalents of monomer at 100 °C in toluene (Table 1, Entry 8).

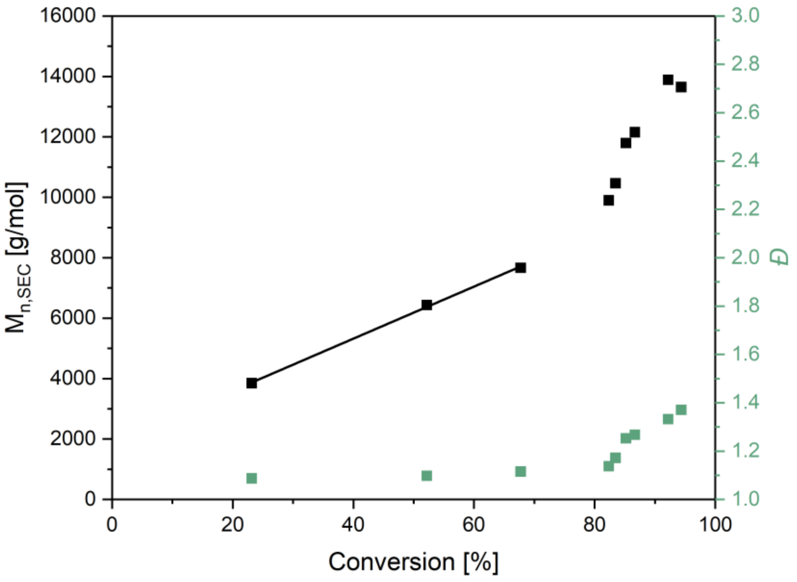

Figure S50: Molar mass and polydispersity plotted over the conversion for 25 equivalents of (+)-carvomenthide at 100 °C (Table 1, Entry 8).

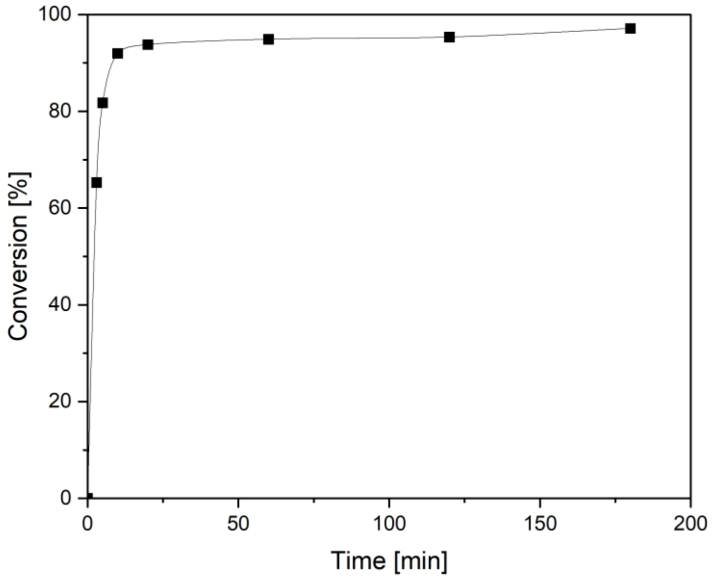

Figure S51: Conversion over reaction time plot for the ROP of (+)-carvomenthide with 50 equivalents of monomer at 100 °C in toluene (Table 1, Entry 11).

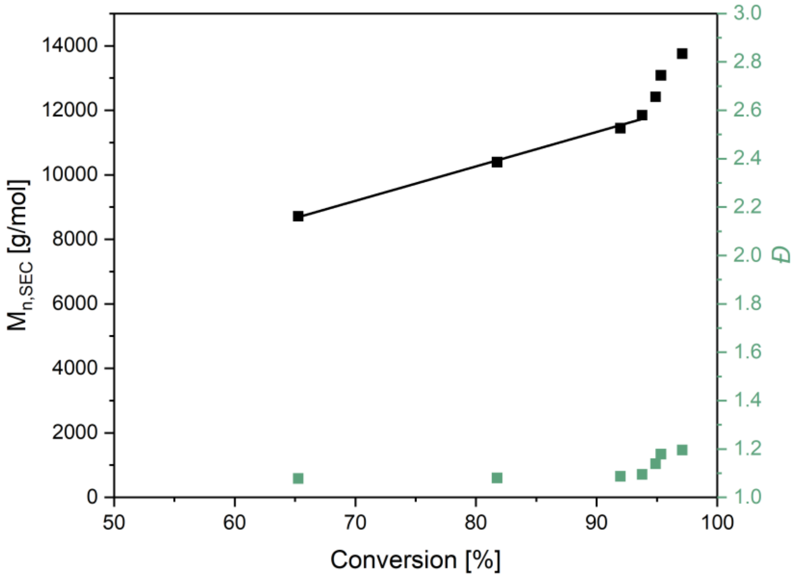

Figure S52: Molar mass and polydispersity plotted over the conversion for 50 equivalents of (+)-carvomenthide at 60 °C (Table 1, Entry 11).

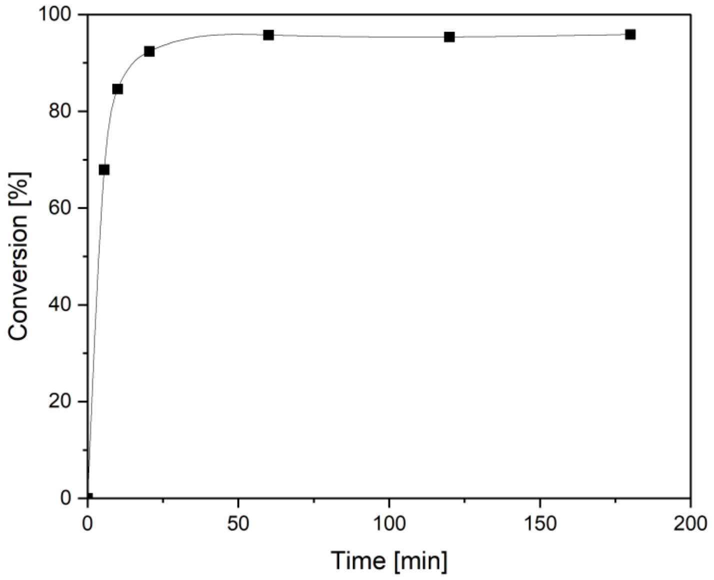

Figure S53: Conversion over reaction time plot for the ROP of (+)-carvomenthide with 100 equivalents of monomer at 60 °C in toluene (Table 1, Entry 12).

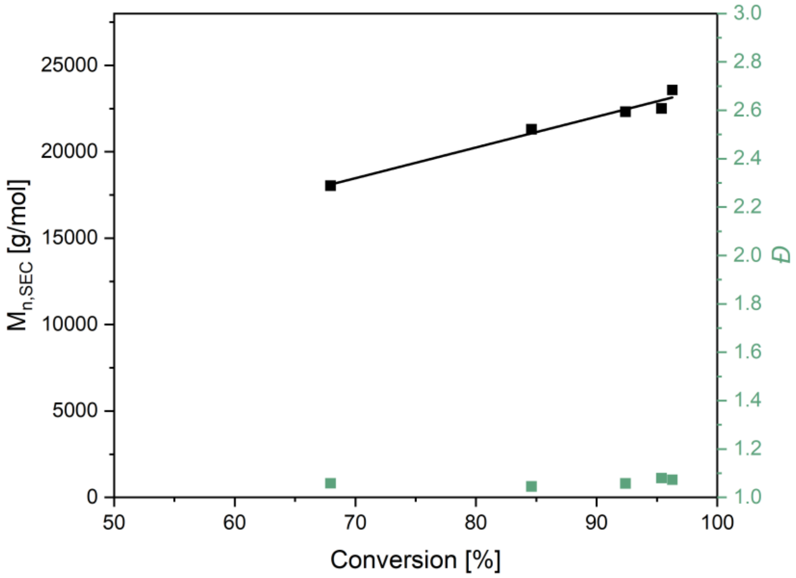

Figure S54: Molar mass and polydispersity plotted over the conversion for 100 equivalents of (+)-carvomenthide at 60 °C (Table 1, Entry 12).

Kinetic Investigation

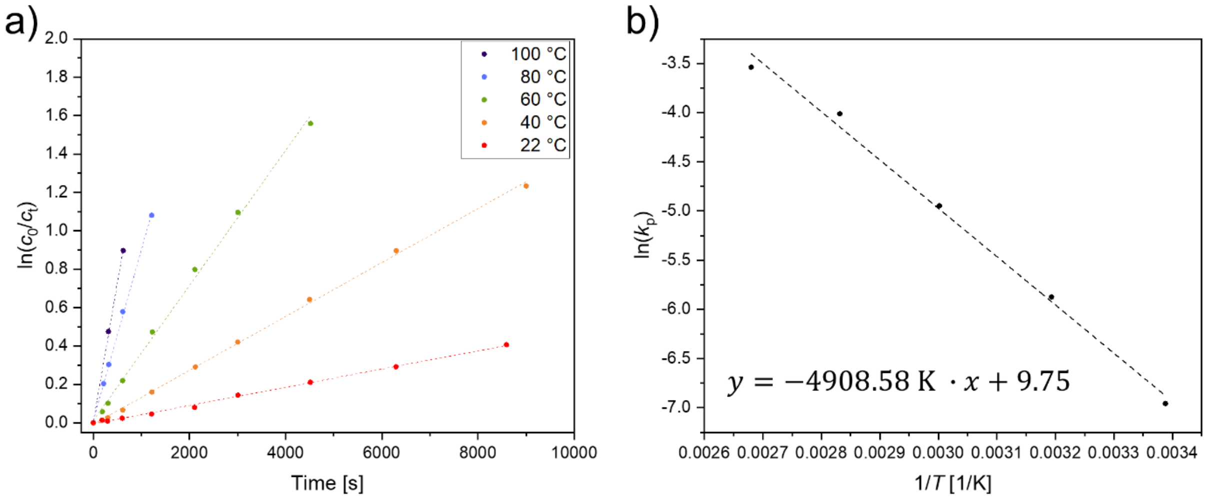

Figure S55: a) Linear first-order kinetic plot for the polymerization of (-)-menthide, and b) Arrhenius-plot for the polymerization of (-)-menthide. The slope corresponds to the activation energy.

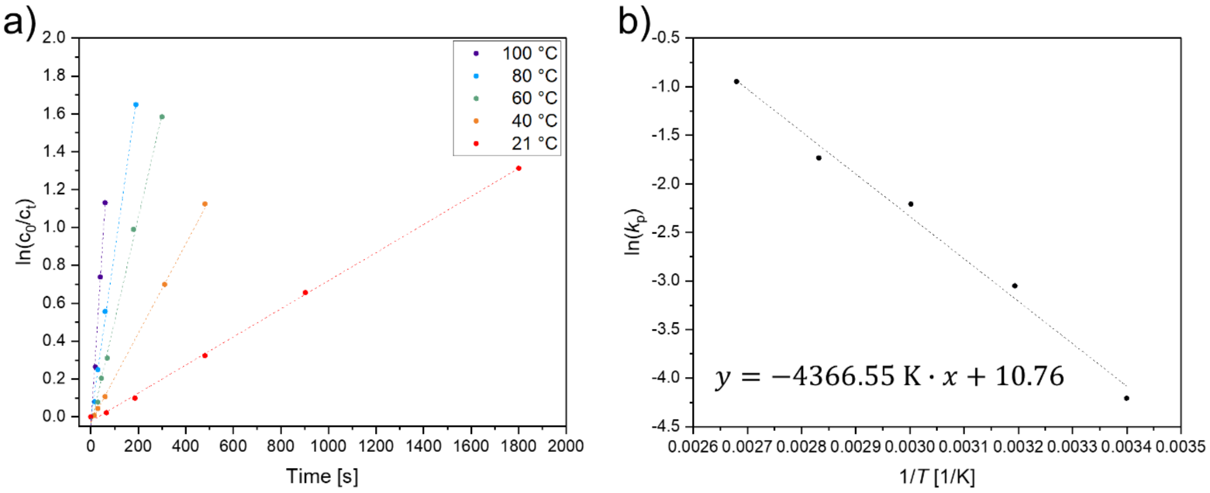

Figure S56: a) Linear first-order kinetic plot for the polymerization of (+)-carvomenthide, and b) Arrhenius-plot for the polymerization of (+)-carvomenthide. The slope corresponds to the activation energy.

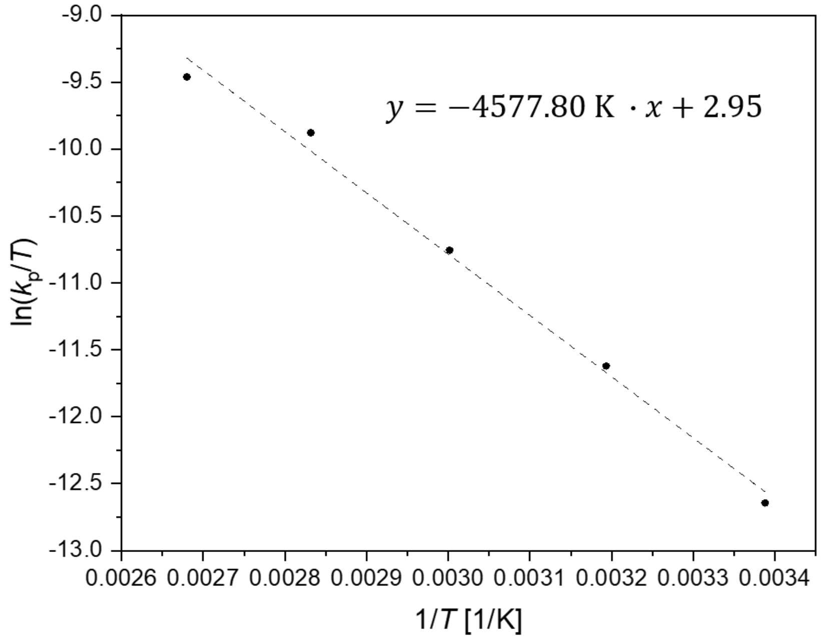

Figure S57: Eyring-Polanyi plot for the polymerization of (-)-menthide.

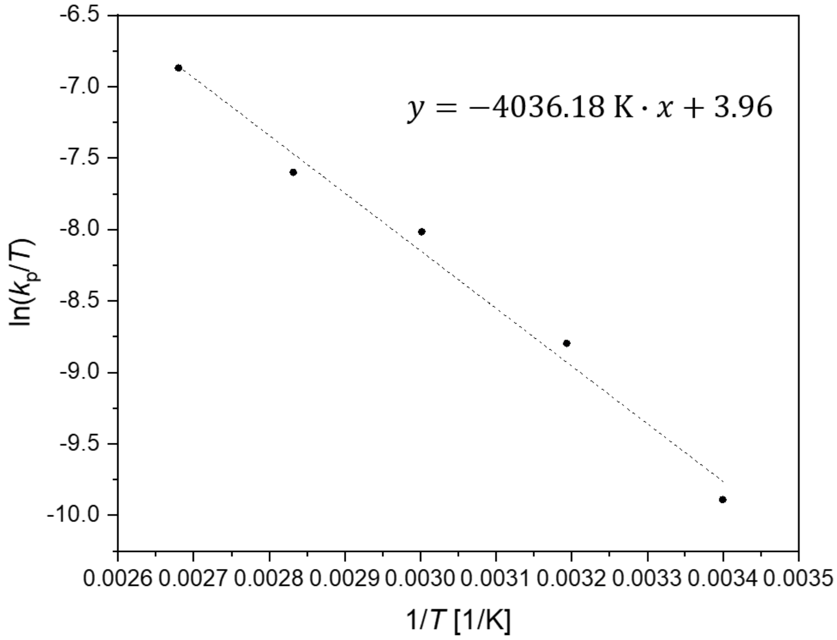

Figure S58: Eyring-Polanyi plot for the polymerization of (+)-carvomenthide.

3.4 Selected Data

Table S2: Polymerizations of (-)-menthide.[a]

**Entry**	**[M]:[Y]: [*i*PrOH]**	***T* [°C]**	**Conversion [%][b]**	**$M_{n, calc}$ [g mol^{-1}][c]**	**$M_{n, SEC}$ [g mol^{-1}][d]**	**Đ[d]**	***I*[e]**	**TOF* [h^{-1}][f]**
**1**	[25]:[1]:[1]	100	98	4200	13900	1.22	0.30	365
**2**	[25]:[1]:[0]	100	>99	4300	10900	1.24	0.39	180
**3**	[25]:[1]:[1]	80	99	4200	10200	1.16	0.42	184
**4**	[25]:[1]:[0]	80	96	4100	11400	1.18	0.36	167

[a] All reactions were performed in 0.5 mL of toluene for 24 h with [(ONOO)^{tBu}Y(bdsa)(THF)] as catalyst and 0-1 equivalents of *i*PrOH. [b] Conversions determined via ^{1}H NMR spectroscopy. [c] $M_{n, calc}$ calculated from $M_{n, calc}$ = ($M_{monomer}$ x ([$M_{monomer}$]/[Y]) x conversion). [d] Number-average molecular weight and polydispersity determined via SEC in chloroform at 40 °C relative to polystyrene standards. [e] Initiator efficiency $I = M_{n, calc}/M_{n, SEC}$ at begin of the plateau region with the relative obtained molar masses. [e] TOF* adjusted using initiator efficiency: TOF* = TOF/*I*.

4. Computational Results

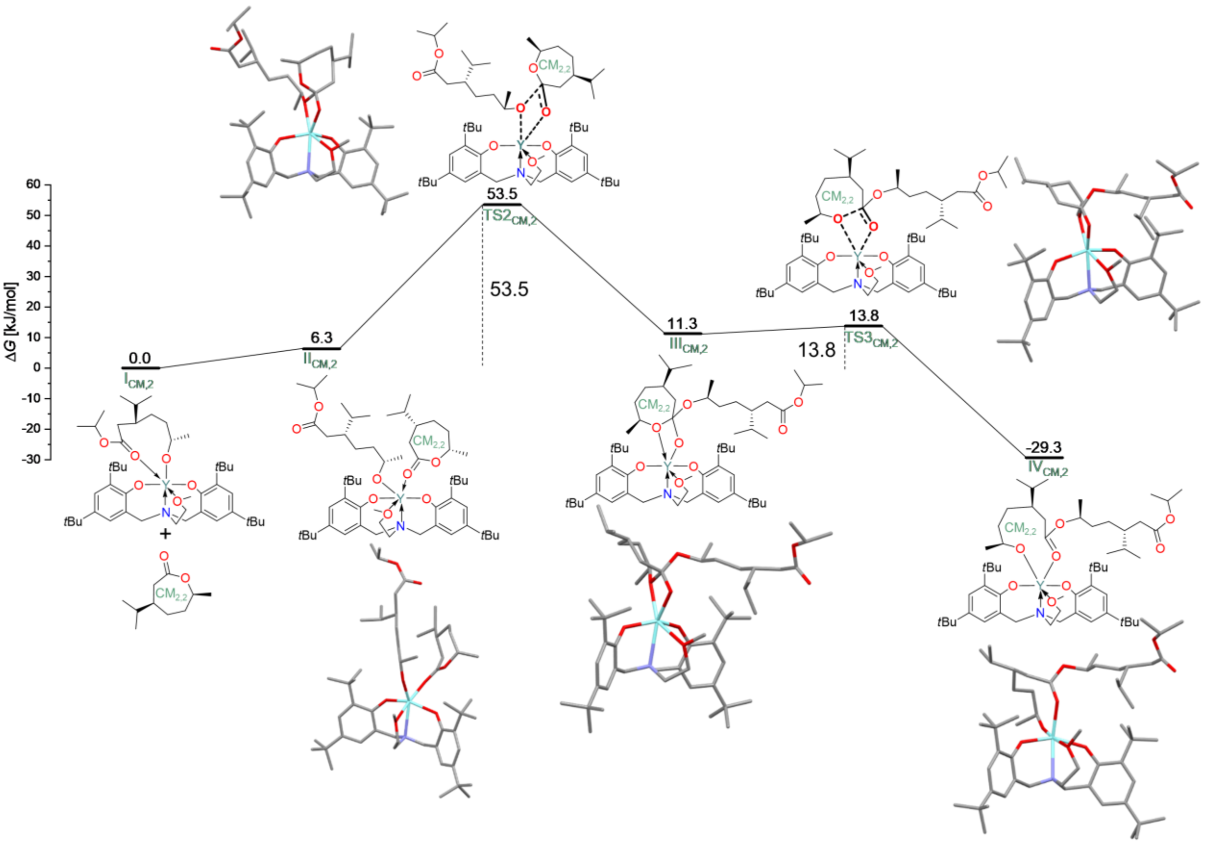

Figure S59: The Gibbs free energy profile (kJ mol^{-1}) for ring-opening polymerization of the *cis* carvomenthide ($CM_{2,2}$) in implicit solvent (toluene). Hydrogen atoms are omitted from the optimized geometries for clarity. Atoms are color-coded as follows: C (grey), Y (cyan), O (red), and N (blue).

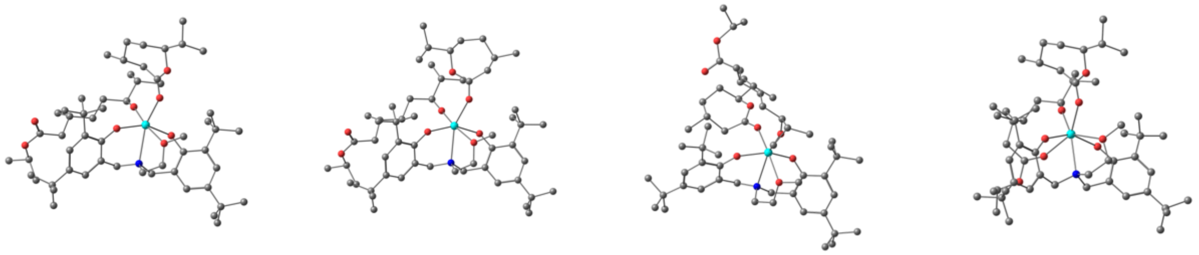

$\mathbf{II}_{\mathbf{M}}$	$\mathbf{II}_{\mathbf{M,si}}$	$\mathbf{II}_{\mathbf{M,axial}}$	$\mathbf{II}_{\mathbf{M,\kappa\text{-}3}}$
0.0 kJ mol^{-1}	11.1 kJ mol^{-1}	8.6 kJ mol^{-1}	7.5 kJ mol^{-1}

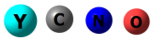

Figure S60: The free energy values for possible isomers of intermediate $\mathbf{II}_{\mathbf{M}}$ arising from insertion of the (-)-menthide monomer (**M2**) into $\mathbf{I}_{\mathbf{M}}$, varying by re/si facial selectivity and equatorial or axial orientation. Hydrogen atoms are omitted from the optimized geometries for clarity.

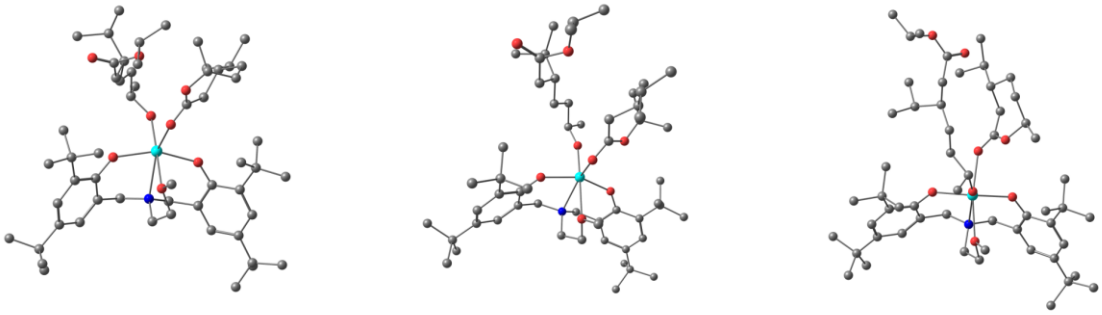

$\mathbf{II}_{\mathbf{CM}}$	$\mathbf{II}_{\mathbf{CM,si}}$	$\mathbf{II}_{\mathbf{CM,axial}}$
0.0 kJ mol^{-1}	4.9 kJ mol^{-1}	11.9 kJ mol^{-1}

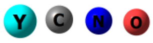

Figure S61: The free energy values for possible ísomers of intermediate $\mathbf{II}_{\mathbf{CM}}$ arising from insertion of the (+)-carvomenthide monomer (**CM**) into $\mathbf{I}_{\mathbf{CM}}$, varying by re/si facial selectivity and equatorial or axial orientation. Hydrogen atoms are omitted from the optimized geometries for clarity.

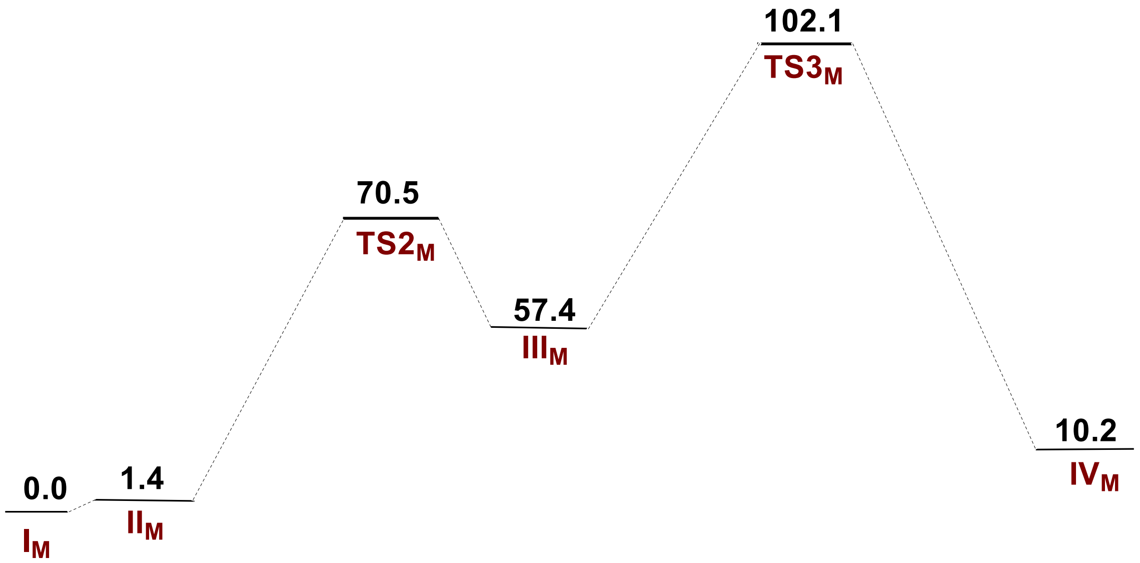

Figure S62: The free energy profile (kJ mol^{-1}) at B3LYP-D3BJ/def2-TZVP//BP86-D3/def2-TZVP (in implicit toluene) level of theory for ring-opening polymerization of (-)-menthide by investigating the second monomer insertion (**M**$_2$).

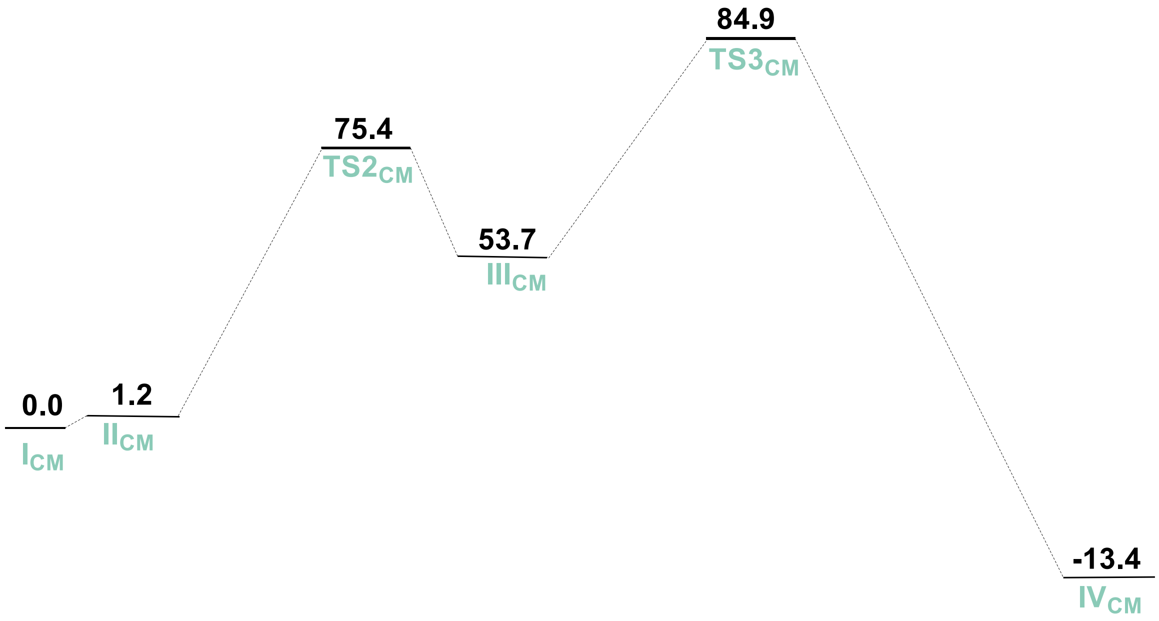

Figure S63: The free energy profile (kJ mol^{-1}) at B3LYP-D3BJ/def2-TZVP//BP86-D3/def2-TZVP (in implicit toluene) level of theory for ring-opening polymerization of trans (+)-carvomenthide by investigating the second monomer insertion (**CM**$_2$).

Structures of Figure 4

BP86-D3/def2-TZVP optimized geometries

M_2 (in implicit toluene)

C	1.147376	-0.494115	-2.715503
C	0.006093	-1.075450	-1.905996
C	0.469150	-1.741087	-0.585636
C	0.747425	-0.712343	0.520669
C	1.438808	1.259229	-0.991846
C	1.794119	0.358121	0.193750
H	-0.747465	-0.302540	-1.683212
H	1.404680	-2.289020	-0.797188
H	-0.204659	-0.211600	0.774953
H	-0.464940	-1.821689	-2.557003
H	1.061833	-1.251394	1.428819
H	1.935413	0.984191	1.085680
H	2.771484	-0.103827	-0.022820
O	1.528728	-0.979137	-3.766217
O	1.807540	0.594206	-2.244009
C	2.176729	2.610211	-1.004094
H	3.254669	2.393629	-0.900922
C	1.950797	3.359014	-2.325419
H	2.306319	2.780462	-3.186039
H	0.878525	3.566704	-2.473346
H	2.480120	4.322223	-2.311968
C	1.730115	3.487019	0.175852
H	2.250553	4.454163	0.144156
H	0.648474	3.689706	0.121887
H	1.940361	3.031548	1.152828
H	0.352976	1.464080	-1.014806
C	-0.583851	-2.753264	-0.117431
H	-1.549257	-2.254838	0.064531
H	-0.745503	-3.539416	-0.868536
H	-0.272995	-3.236735	0.820425

I_M (in implicit toluene)

C	0.267073	-5.441588	4.395057
C	-0.445374	-4.488935	3.656288
C	-1.862640	-4.531419	3.592614
C	-2.550486	-5.543826	4.333207
C	-1.783381	-6.469466	5.046447
C	-0.377664	-6.462291	5.095809
C	0.300707	-3.426442	2.897575
N	-0.000196	-2.014757	3.309693
C	0.899980	-1.124314	2.508999
C	0.880275	0.331663	2.882635
C	-0.333427	1.060562	2.758157
C	-0.345641	2.439870	3.113611
C	0.855566	3.015621	3.556725
C	2.062796	2.313934	3.681939
C	2.039917	0.954939	3.340881
O	-1.425926	0.415412	2.359774
Y	-2.444744	-1.477845	2.528742
C	-1.655372	3.246046	3.083393
C	-2.299446	3.198817	1.678448
C	3.367554	2.967146	4.163170
C	3.179772	4.447081	4.538787
O	-2.508786	-3.643072	2.849548
C	-4.087822	-5.607928	4.331955
C	-4.682921	-4.266046	4.823429
C	0.366596	-7.534412	5.905292
C	-0.052909	-7.446525	7.390195
O	-2.200184	-1.302551	4.925765
C	-3.266157	-0.823726	5.764370
O	-4.446556	-0.976280	2.306185
C	-5.498749	-0.316144	1.653988
C	-6.546722	0.178738	2.679913
C	-7.772718	0.827644	2.022638
C	0.146836	-1.830328	4.772056
C	-0.882211	-0.874853	5.362802
C	-2.626757	2.636923	4.119353
C	-1.444746	4.726598	3.450194
C	3.894211	2.219563	5.409455
C	4.425260	2.886398	3.038741
C	-4.583716	-5.896313	2.897828
C	-4.630946	-6.717440	5.250667
C	0.009625	-8.935161	5.358365

C	1.893864	-7.367135	5.827822
C	-6.132813	-1.214062	0.570174
C	-5.107229	-1.925176	-0.334689
C	-5.899789	1.154053	3.665611
H	1.157075	-1.472888	5.040163
H	-0.000873	-2.812292	5.241147
H	-0.837682	-0.931235	6.463000
H	-0.725888	0.168626	5.050890
H	-3.169919	-1.254329	6.773174
H	-4.203615	-1.158257	5.305174
H	-3.254003	0.275588	5.822188
H	0.594608	-1.252604	1.456118
H	1.939362	-1.498801	2.594421
H	0.843797	4.068209	3.828308
H	2.947796	0.352120	3.428133
H	1.388813	-3.610785	2.998664
H	0.061634	-3.481699	1.823704
H	-2.305037	-7.246093	5.604941
H	1.355204	-5.361550	4.407535
H	-3.573482	3.196826	4.139914
H	-2.184379	2.668848	5.126807
H	-2.855470	1.595587	3.864560
H	-0.754789	5.226534	2.753938
H	-1.054835	4.849191	4.471564
H	-2.409304	5.252201	3.399212
H	-1.621917	3.637146	0.930427
H	-3.234526	3.780298	1.675326
H	-2.526355	2.168213	1.383974
H	3.158078	2.261041	6.225952
H	4.831580	2.674795	5.765032
H	4.097017	1.161550	5.191547
H	4.632445	1.844737	2.755738
H	5.372812	3.343693	3.364364
H	4.076274	3.416756	2.140565
H	2.841314	5.046026	3.680512
H	4.138049	4.865699	4.879736
H	2.451671	4.571100	5.354049
H	-5.683988	-5.878334	2.857680
H	-4.242901	-6.887637	2.563117

H	-4.201517	-5.137197	2.207045
H	-4.337457	-6.561661	6.299683
H	-4.289231	-7.716128	4.941056
H	-5.730054	-6.716622	5.207731
H	-4.279587	-4.004299	5.813019
H	-5.777349	-4.348806	4.909950
H	-4.457354	-3.451311	4.127158
H	-1.069203	-9.132261	5.426151
H	0.532451	-9.718159	5.929507
H	0.302283	-9.024499	4.301771
H	2.258856	-7.445236	4.792927
H	2.384264	-8.157392	6.415109
H	2.219479	-6.398868	6.235647
H	0.196741	-6.458630	7.804421
H	0.467041	-8.213100	7.985477
H	-1.134188	-7.599690	7.514903
H	-5.095635	0.588911	1.140668
H	-6.775897	-1.959064	1.069529
H	-6.799764	-0.595062	-0.047245
H	-4.193742	-1.317031	-0.386708
C	-4.763668	-3.348357	0.147696
H	-5.496007	-1.999624	-1.365376
H	-6.870370	-0.721453	3.234187
H	-5.597640	2.078964	3.148542
H	-6.593067	1.429574	4.474608
H	-4.997915	0.717899	4.108231
H	-8.437215	1.259746	2.786002
H	-7.464787	1.645884	1.351295
H	-8.364256	0.112646	1.434844
C	-3.499226	-3.948148	-0.498214
H	-4.595436	-3.303684	1.235108
C	-5.928356	-4.312214	-0.124125
C	-2.173877	-3.361042	-0.083855
H	-3.549655	-3.844660	-1.599231
H	-3.482254	-5.028094	-0.303256
O	-1.052815	-4.066055	-0.213568
O	-2.038613	-2.199623	0.330946
C	-1.017561	-5.507879	-0.555748
C	-1.266989	-6.317392	0.706777

H	-1.789399	-5.698300	-1.314996
C	0.359917	-5.732295	-1.153805
H	-1.260369	-7.388414	0.462377
H	-0.488045	-6.118751	1.454837
H	-2.230550	-6.070862	1.169989
H	0.467849	-6.787325	-1.439912
H	0.509338	-5.110505	-2.045904
H	1.138918	-5.490542	-0.417742
H	-5.732576	-5.310643	0.292987
H	-6.860851	-3.944245	0.323735
H	-6.097702	-4.419821	-1.207901

II$_{M}$ (in implicit toluene)

C	0.185205	-5.539499	3.605164
C	-0.684793	-4.441145	3.594171
C	-1.944903	-4.515274	4.244217
C	-2.338902	-5.747194	4.852432
C	-1.430225	-6.806199	4.828073
C	-0.159283	-6.741685	4.228096
C	-0.316596	-3.208653	2.807239
N	-0.413404	-1.888806	3.526555
C	0.247110	-0.861907	2.658862
C	0.179574	0.564864	3.135841
C	-1.087361	1.209770	3.189424
C	-1.141398	2.593021	3.536730
C	0.054710	3.218105	3.919868
C	1.305312	2.580360	3.932368
C	1.339299	1.247789	3.501162
O	-2.176628	0.505568	2.912532
Y	-2.915200	-1.377567	3.708869
O	-4.595642	-0.709983	5.191689
C	-5.820970	-0.850003	5.339592
O	-6.314493	-0.433502	6.497084
C	-7.739907	-0.615134	6.851657
C	-7.954085	0.254558	8.099127
C	-9.436035	0.265337	8.502715
C	-2.467397	3.372935	3.447053
C	-3.034477	3.242544	2.012987
C	2.597321	3.278072	4.384081
C	2.358076	4.734624	4.817496
O	-2.734730	-3.455124	4.243977
C	-3.754279	-5.908508	5.429494
C	-4.014197	-4.870950	6.545720
C	0.774973	-7.957929	4.307495
C	1.127739	-8.225198	5.789205
O	-1.618429	-0.690045	5.800551
C	-1.997054	0.435761	6.611286
O	-4.156409	-1.732214	2.096497
C	-4.585183	-1.875257	0.774398
C	-5.865833	-1.038389	0.529884
C	-6.347991	-1.113415	-0.924519
C	0.196399	-1.991401	4.870312
C	-0.186275	-0.866489	5.816494
C	-3.502912	2.827757	4.456549
C	-2.282590	4.873294	3.740767
C	3.195794	2.511959	5.586597
C	3.619052	3.282920	3.224707
C	-4.772587	-5.723078	4.280010
C	-3.984250	-7.309222	6.024288
C	0.068237	-9.202632	3.725572
C	2.089418	-7.737005	3.539472
C	-4.774532	-3.368036	0.435070
C	-3.552305	-4.208761	0.802009
C	-5.633968	0.418749	0.945824
C	-6.687489	-1.488468	4.299057
C	-7.151634	-2.908674	4.718706
C	-7.564738	-3.683508	3.465508
C	-8.295608	-2.875077	5.740759
C	-8.018495	-2.106849	7.038303
C	-7.464921	1.691529	7.865005
H	1.302024	-2.031704	4.805667
H	-0.140929	-2.944461	5.297083
H	0.132281	-1.139212	6.838931
H	0.290504	0.086306	5.543267
H	-1.663787	0.283554	7.651114
H	-3.087867	0.504338	6.574544
H	-1.548423	1.357031	6.204659

H	-0.253991	-0.939532	1.679222
H	1.307749	-1.148100	2.513658
H	0.009716	4.263470	4.215610
H	2.288538	0.705877	3.464093
H	0.715057	-3.332682	2.425674
H	-0.971648	-3.120085	1.921396
H	-1.726853	-7.746980	5.288108
H	1.147575	-5.433920	3.101413
H	-4.458145	3.364318	4.342934
H	-3.154714	2.973878	5.489044
H	-3.688817	1.759693	4.298865
H	-1.576435	5.343507	3.040298
H	-1.924868	5.053677	4.765441
H	-3.250302	5.384809	3.633254
H	-2.323359	3.653476	1.280835
H	-3.977781	3.804294	1.927917
H	-3.229587	2.193740	1.762530
H	2.485915	2.497772	6.427044
H	4.127055	2.990055	5.928514
H	3.428226	1.470102	5.324162
H	3.866903	2.262514	2.900362
H	4.554485	3.774155	3.535399
H	3.215863	3.824271	2.356341
H	1.959268	5.344838	3.993675
H	3.308958	5.185751	5.137001
H	1.657859	4.798228	5.663542
H	-5.800424	-5.811603	4.666118
H	-4.622318	-6.504996	3.521573
H	-4.660869	-4.740826	3.803669
H	-3.297993	-7.519716	6.858372
H	-3.866495	-8.095742	5.264945
H	-5.011545	-7.372514	6.413480
H	-3.306192	-5.010188	7.376854
H	-5.034012	-4.996026	6.944379
H	-3.904293	-3.847627	6.168062
H	-0.838258	-9.457334	4.291203
H	0.739227	-10.075013	3.757259
H	-0.235025	-9.036077	2.683930
H	1.914681	-7.547512	2.471114

H	2.719281	-8.634740	3.619686
H	2.662152	-6.891054	3.948397
H	1.639432	-7.356362	6.229184
H	1.791612	-9.099477	5.877663
H	0.225807	-8.423502	6.385002
H	-3.801407	-1.470206	0.090730
H	-5.651740	-3.742528	0.991895
H	-5.006548	-3.473225	-0.635803
H	-3.291324	-3.989524	1.847295
C	-3.738745	-5.726497	0.661016
H	-2.689786	-3.890793	0.186408
H	-6.650234	-1.469276	1.180269
H	-4.866543	0.883529	0.306531
H	-6.556028	1.011572	0.851978
H	-5.274485	0.485231	1.980927
H	-7.201622	-0.438800	-1.087730
H	-5.545167	-0.805306	-1.614132
H	-6.665890	-2.125912	-1.208616
H	-7.564326	-0.855299	4.087569
H	-6.285328	-3.422195	5.169558
H	-9.185221	-2.436925	5.253332
H	-6.076727	-1.552048	3.387318
H	-8.564667	-3.912364	5.994727
H	-8.890535	-2.217009	7.696607
H	-7.162963	-2.542948	7.579545
H	-7.363567	-0.194898	8.916646
H	-6.396663	1.726720	7.619792
H	-8.021347	2.157876	7.036498
H	-7.631010	2.300116	8.764517
H	-9.580305	0.918931	9.373571
H	-10.059834	0.658299	7.684298
H	-9.814281	-0.729800	8.770807
H	-8.344040	-0.198841	6.026347
H	-8.386672	-3.169976	2.942341
H	-6.720779	-3.777029	2.769623
H	-7.903384	-4.696423	3.724779
C	-2.494013	-6.431310	1.244980
H	-4.598727	-6.023751	1.284467
C	-4.012963	-6.162846	-0.782795

C	-2.671536	-7.927941	1.353878
H	-1.604688	-6.137586	0.673696
H	-2.340359	-6.086897	2.276876
O	-1.959850	-8.776040	0.555231
O	-3.455292	-8.427548	2.142382
C	-1.057570	-8.304250	-0.498810
C	-1.051128	-9.404555	-1.548983
H	-1.478890	-7.388836	-0.937791
C	0.321059	-8.026974	0.084156
H	-0.395521	-9.122123	-2.384572
H	-0.680363	-10.344102	-1.115966
H	-2.063515	-9.574104	-1.938592
H	0.994602	-7.650812	-0.698999
H	0.268210	-7.277651	0.886281
H	0.751336	-8.947261	0.502058
H	-4.094538	-7.257345	-0.866448
H	-4.952083	-5.734122	-1.158052
H	-3.203690	-5.827554	-1.451904

$TS2_M$ (in implicit toluene)

C	0.678524	-2.361927	2.131778
C	-0.674987	-2.418981	2.458259
C	-1.280471	-1.397255	3.242067
C	-0.490394	-0.274256	3.626783
C	0.866559	-0.271850	3.265114
C	1.490402	-1.294443	2.536231
C	-1.511837	-3.579786	1.997363
N	-2.075784	-4.450850	3.072202
C	-1.023043	-4.996369	3.958018
C	-0.654133	-4.083162	5.114649
O	-1.859283	-3.712351	5.821849
C	-1.568905	-3.178076	7.123523
O	-2.547527	-1.542217	3.610946
Y	-3.834085	-3.087282	4.465135
O	-4.806405	-3.085536	2.298501
C	-4.989485	-2.392565	1.289072
O	-5.588962	-3.007474	0.267882
C	-5.863106	-2.335034	-1.019310
C	-4.673532	-2.560370	-1.938723
C	-1.095245	0.877219	4.450235
C	-0.115136	2.054232	4.614159
C	2.987368	-1.286754	2.190736
C	3.169585	-1.358857	0.657745
C	-2.818263	-5.560987	2.381214
C	-3.293341	-6.688873	3.258897
C	-3.009706	-8.007601	2.879949
C	-3.451469	-9.101618	3.626205
C	-4.151407	-8.813379	4.811441
C	-4.443431	-7.520757	5.253046
C	-4.041452	-6.417798	4.433815
O	-4.333114	-5.164378	4.753597
C	-5.124030	-7.272879	6.605653
C	-5.507175	-8.576006	7.329237
C	-3.203767	-10.562532	3.220238
C	-2.403356	-10.676662	1.911619
O	-4.117061	-1.746523	6.242538
C	-5.376315	-1.795580	6.595174
C	-5.665910	-2.812986	7.702351
C	-5.015409	-2.425646	9.048090
C	-5.937266	-1.500199	9.849908
C	-6.255082	-0.174946	9.160544
C	-6.926908	-0.261198	7.779255
C	-7.629225	1.080873	7.461187
C	-7.866534	1.335296	5.972163
O	-5.934425	-0.524136	6.747016
C	-8.961158	1.159752	8.223962
C	-2.361975	1.422812	3.753656
C	-1.472276	0.362329	5.857812
C	3.700950	-0.020998	2.696955
C	3.665742	-2.514544	2.842211
C	-4.129234	-6.510217	7.511523
C	-6.414138	-6.445645	6.423630
C	-2.413746	-11.287082	4.333743
C	-4.561904	-11.272591	3.016260
C	-4.661300	-3.672573	9.864619
C	-4.621768	-0.940672	1.230611
C	-5.784774	-0.023367	1.714562

C	-5.372213	1.439533	1.516455
C	-6.179093	-0.315097	3.178125
C	-7.217129	-1.433186	3.321370
C	-7.339768	-2.084715	4.713308
C	-8.304214	-3.297764	4.673056
C	-8.639521	-3.788778	6.082147
O	-6.058945	-2.484082	5.218835
C	-9.609884	-2.997672	3.918024
C	-7.157639	-2.946472	-1.526164
H	-0.102899	-5.235848	3.390183
H	-1.410100	-5.935066	4.374816
H	0.007834	-4.638344	5.801906
H	-0.136960	-3.167788	4.789263
H	-1.112580	-3.958604	7.753503
H	-2.522551	-2.846704	7.544462
H	-0.887491	-2.315666	7.044042
H	-2.377039	-3.215470	1.422351
H	-0.905515	-4.205531	1.312801
H	1.473384	0.573994	3.578670
H	1.102047	-3.187560	1.552982
H	-2.167640	-5.986390	1.592127
H	-3.669579	-5.082828	1.873795
H	-4.476857	-9.651687	5.426851
H	-2.429607	-8.158903	1.967929
H	-1.880906	1.184969	6.466245
H	-0.579049	-0.027912	6.369875
H	-2.234435	-0.423706	5.803523
H	0.196181	2.465787	3.642019
H	0.787793	1.768316	5.173539
H	-0.608896	2.859501	5.177697
H	-2.127110	1.759301	2.732165
H	-2.765468	2.279398	4.315440
H	-3.135106	0.649108	3.702312
H	3.556570	-2.479723	3.936291
H	4.740495	-2.537913	2.603300
H	3.221386	-3.455612	2.488229
H	2.713477	-2.265836	0.237083
H	4.239522	-1.366774	0.396922
H	2.702597	-0.489522	0.171955

H	3.275958	0.890757	2.252306
H	4.765690	-0.061976	2.424100
H	3.641315	0.070200	3.791469
H	-6.868346	-6.234282	7.404639
H	-7.149427	-7.000788	5.821673
H	-6.205032	-5.497272	5.919547
H	-4.630214	-9.201618	7.553111
H	-6.219252	-9.175571	6.742709
H	-5.990165	-8.327537	8.286343
H	-3.226627	-7.116457	7.681643
H	-4.589916	-6.297652	8.487188
H	-3.822225	-5.562468	7.051638
H	-5.166359	-11.256137	3.933653
H	-4.411328	-12.325128	2.728417
H	-5.140937	-10.777988	2.222628
H	-2.934697	-10.208456	1.069811
H	-2.250886	-11.737027	1.661667
H	-1.412463	-10.206639	1.997240
H	-1.439789	-10.801819	4.496270
H	-2.235549	-12.338615	4.059303
H	-2.957472	-11.277346	5.288730
H	-7.748521	-1.334428	5.409610
H	-7.026734	-2.239512	2.598882
H	-8.199307	-1.017028	3.050779
H	-5.276406	-0.528188	3.773069
H	-6.591803	0.604141	3.612404
H	-7.762570	-4.101064	4.141829
H	-9.162831	-3.002851	6.652278
H	-9.293551	-4.672324	6.046671
H	-7.736740	-4.069342	6.628477
H	-10.304753	-3.844365	4.017120
H	-10.109269	-2.109522	4.339205
H	-9.454176	-2.823282	2.845474
H	-6.741593	-2.971005	7.848252
H	-4.084740	-1.871807	8.822836
H	-6.877725	-2.041626	10.061857
H	-5.257845	-3.763771	7.335404
H	-5.477751	-1.287584	10.829726
H	-6.900059	0.418454	9.826177

H	-5.330194	0.408909	9.019033
H	-6.964719	1.882917	7.830006
H	-6.924591	1.302606	5.416130
H	-8.546742	0.586494	5.538606
H	-8.325701	2.324697	5.828317
H	-9.424627	2.147828	8.090269
H	-9.665478	0.404525	7.840093
H	-8.844721	0.989463	9.303145
H	-7.688510	-1.063706	7.760365
H	-5.552244	-4.300103	10.028067
H	-3.913115	-4.287218	9.346434
H	-4.256246	-3.399161	10.850861
H	-6.667293	-0.216301	1.077758
H	-4.317906	-0.649785	0.217209
H	-3.762798	-0.816147	1.907044
H	-6.012503	-1.263431	-0.827813
H	-7.433474	-2.482285	-2.482924
H	-7.038156	-4.027274	-1.682738
H	-7.972434	-2.780733	-0.809716
H	-4.863626	-2.093755	-2.915092
H	-3.756923	-2.122046	-1.521263
H	-4.509365	-3.636272	-2.090015
H	-5.077068	1.639592	0.475531
H	-6.206869	2.108939	1.769580
H	-4.524285	1.696726	2.165435

III_M (in implicit toluene)

C	4.540433	-1.220920	0.481424
C	3.275073	-0.777561	0.098957
C	2.832679	0.533932	0.429135
C	3.717150	1.384111	1.155721
C	4.984060	0.887707	1.499282
C	5.433010	-0.401962	1.184159
C	2.354788	-1.710593	-0.637686
N	1.060240	-2.005346	0.045648
C	1.263207	-2.480600	1.436815
C	1.202230	-1.371960	2.477781
O	-0.003501	-0.603927	2.264024
C	-0.425547	0.106316	3.440934
O	1.601311	0.906281	0.093688
Y	-0.442786	0.134297	-0.044432
O	0.085783	-0.063386	-2.352492
C	0.825772	0.406042	-3.227507
O	0.806197	-0.229735	-4.401100
C	1.676099	0.153529	-5.532670
C	2.963113	-0.650509	-5.433504
C	3.266736	2.783971	1.600585
C	4.371696	3.552598	2.348226
C	6.817571	-0.932665	1.584653
C	7.606441	-1.324174	0.314749
C	0.340367	-3.032763	-0.793020
C	-0.863726	-3.636178	-0.128499
C	-0.895871	-5.010869	0.138029
C	-1.962886	-5.601445	0.816668
C	-2.986356	-4.739442	1.250549
C	-3.006445	-3.361365	1.019984
C	-1.924744	-2.786512	0.278483
O	-1.865980	-1.493021	-0.015053
C	-4.137949	-2.477819	1.564743
C	-5.192333	-3.274683	2.353242
C	-2.048242	-7.104760	1.119359
C	-0.836483	-7.878810	0.573265
O	-1.386603	1.799898	1.059875
C	-2.350906	2.310398	0.297116
C	-3.771937	1.963173	0.753190
C	-4.098096	2.306718	2.208439
C	-3.855072	3.780646	2.556152
C	-2.387249	4.217218	2.474812
C	-1.919234	4.636067	1.079362
C	-0.417927	4.992539	0.996132
C	-0.150407	5.814343	-0.270027
O	-2.243351	3.706706	0.004368
C	0.102516	5.760012	2.217497
C	2.867730	3.625484	0.370250
C	2.062475	2.644403	2.558836
C	7.643085	0.108437	2.360037
C	6.650769	-2.179432	2.482916

C	-3.542940	-1.420492	2.523413
C	-4.852992	-1.788335	0.383871
C	-2.109665	-7.324276	2.648315
C	-3.324355	-7.688822	0.470990
C	-5.549682	1.912652	2.511166
C	1.671137	1.629303	-3.010574
C	0.986312	2.955881	-3.456802
C	1.978613	4.107363	-3.261654
C	-0.326094	3.212054	-2.692828
C	-1.523963	2.457463	-3.271881
C	-2.677427	2.128118	-2.305799
C	-3.618627	1.077106	-2.947988
C	-4.913812	0.864178	-2.163932
O	-2.135232	1.616368	-1.058881
C	-3.976999	1.431445	-4.403092
C	0.864492	-0.136780	-6.782691
H	2.223708	-3.017000	1.543199
H	0.455711	-3.188956	1.658806
H	1.161704	-1.834021	3.478898
H	2.068165	-0.694373	2.436226
H	-0.781334	-0.610443	4.197909
H	-1.233894	0.775666	3.129843
H	0.403909	0.703144	3.849501
H	2.094799	-1.303096	-1.626073
H	2.888817	-2.665032	-0.815672
H	5.650893	1.541745	2.054849
H	4.823716	-2.244499	0.221927
H	1.047348	-3.842239	-1.056833
H	0.065661	-2.518342	-1.728588
H	-3.813712	-5.175974	1.809552
H	-0.045478	-5.611276	-0.190680
H	1.756266	3.630735	2.935088
H	2.342399	2.023149	3.423266
H	1.201921	2.187031	2.057086
H	5.264789	3.700856	1.723072
H	4.677960	3.042588	3.273511
H	3.991290	4.545724	2.628790
H	3.710820	3.701737	-0.333044
H	2.585478	4.643458	0.677292

H	2.015538	3.172210	-0.147825
H	6.091279	-1.926169	3.395488
H	7.633808	-2.577499	2.779429
H	6.105597	-2.981054	1.965193
H	7.082982	-2.100506	-0.260733
H	8.601177	-1.714215	0.581565
H	7.743097	-0.451907	-0.341441
H	7.820419	1.013991	1.761675
H	8.623260	-0.316631	2.621638
H	7.148284	0.405999	3.296135
H	-5.617818	-1.084365	0.747311
H	-5.351398	-2.535470	-0.252105
H	-4.131844	-1.239076	-0.230818
H	-4.760860	-3.767983	3.237187
H	-5.681245	-4.040275	1.732280
H	-5.973971	-2.585361	2.705586
H	-3.010135	-1.911235	3.351303
H	-4.342948	-0.798874	2.952277
H	-2.836836	-0.765600	2.003940
H	-4.231464	-7.201016	0.854543
H	-3.406364	-8.766865	0.680956
H	-3.302537	-7.549526	-0.619861
H	-0.751241	-7.781028	-0.519034
H	-0.944075	-8.948372	0.806492
H	0.104786	-7.531700	1.024506
H	-1.209496	-6.919279	3.133701
H	-2.175645	-8.398374	2.882035
H	-2.983967	-6.828958	3.093116
H	-3.237597	3.047260	-2.067628
H	-1.197480	1.507416	-3.718269
H	-1.934955	3.043937	-4.106358
H	-0.187432	2.970425	-1.627750
H	-0.537941	4.289163	-2.721545
H	-3.048314	0.130504	-2.945851
H	-5.500573	1.795447	-2.120934
H	-5.534120	0.096949	-2.650171
H	-4.723959	0.533062	-1.141771
H	-4.718012	0.716513	-4.788341
H	-4.426853	2.435959	-4.461104

H	-3.113637	1.405445	-5.079799
H	-4.485567	2.469247	0.085853
H	-3.430950	1.698827	2.844326
H	-4.472721	4.417425	1.896188
H	-3.890476	0.879879	0.620463
H	-4.221718	3.954940	3.581478
H	-2.247433	5.097285	3.119195
H	-1.735634	3.422256	2.865923
H	0.107121	4.028537	0.929962
H	-0.664034	5.382106	-1.134429
H	-0.516256	6.845950	-0.138838
H	0.923616	5.864032	-0.500696
H	1.153066	6.048638	2.061428
H	-0.471499	6.687991	2.375338
H	0.055182	5.168823	3.141001
H	-2.501081	5.537206	0.804695
H	-6.249023	2.530552	1.925525
H	-5.742716	0.861035	2.253299
H	-5.787238	2.051208	3.576482
H	0.750137	2.884009	-4.534002
H	2.633642	1.523817	-3.528532
H	1.866380	1.672099	-1.929051
H	1.881297	1.230714	-5.467632
H	1.451359	0.128405	-7.672522
H	0.609035	-1.203826	-6.837364
H	-0.063503	0.449178	-6.788226
H	3.625399	-0.397735	-6.272618
H	3.498176	-0.437765	-4.497624
H	2.742056	-1.725801	-5.471924
H	2.927317	3.922926	-3.787401
H	1.552472	5.046411	-3.641725
H	2.202729	4.247485	-2.195380

TS3$_M$ (in implicit toluene)

C	0.977084	-5.432816	5.771858
C	-0.252115	-4.924233	5.334772
C	-1.422699	-5.051010	6.127058
C	-1.332867	-5.817433	7.335579
C	-0.079746	-6.306237	7.716571
C	1.102340	-6.120588	6.978824
C	-0.342151	-4.313484	3.967149
N	-0.797234	-2.889425	3.901878
C	-0.598903	-2.468195	2.489996
C	-1.056304	-1.101359	2.060125
C	-2.455097	-0.796330	2.050480
C	-2.874039	0.321099	1.267667
C	-1.878071	1.175914	0.754639
C	-0.505309	0.968665	0.886299
C	-0.123342	-0.225360	1.516366
O	-3.272201	-1.571638	2.753871
Y	-3.406852	-2.911390	4.478271
O	-5.490384	-3.802914	4.472704
C	-6.178725	-3.312551	5.436693
O	-4.957088	-1.901596	5.915890
C	-5.576204	-0.711257	6.400338
C	-4.649739	0.523257	6.241194
C	-5.205466	1.779253	6.933480
C	-4.323803	0.636763	0.834538
C	-4.385503	0.614670	-0.715029
C	0.555003	1.935404	0.341949
C	-0.068026	3.186596	-0.300523
O	-2.551249	-4.475223	5.723758
C	-2.581781	-6.111155	8.178236
C	-3.160538	-4.785792	8.709943
C	2.433790	-6.675691	7.504876
C	2.747624	-6.041694	8.879274
O	-2.117779	-1.347317	5.880131
C	-1.980203	-1.725762	7.263163
O	-7.361142	-2.681417	5.159375
C	-7.596279	-2.267978	3.776408
C	-8.863700	-1.396777	3.772984
C	-9.063200	-0.783548	2.378245
C	-0.038896	-2.024264	4.828522
C	-0.903043	-0.856231	5.273392
C	-5.357450	-0.386099	1.320266
C	-4.747564	2.039624	1.322635
C	1.470049	2.393654	1.500139

C	1.409251	1.218445	-0.727751
C	-3.629612	-6.836144	7.302021
C	-2.288647	-7.011149	9.391860
C	2.331374	-8.209851	7.664326
C	3.606711	-6.369565	6.557580
C	-7.735975	-3.509091	2.858299
C	-6.629902	-3.649169	1.799453
C	-8.859654	-0.283511	4.825242
C	-6.277003	-4.107248	6.729083
C	-6.309257	-3.410847	8.098836
C	-6.908211	-4.382043	9.125069
C	-7.006356	-2.042002	8.137837
C	-6.067911	-0.864777	7.845946
C	-4.416699	0.839696	4.765217
H	0.894922	-1.651256	4.365767
H	0.244920	-2.624923	5.704028
H	-0.367349	-0.207573	5.985740
H	-1.227298	-0.250533	4.417543
H	-1.337709	-2.611174	7.376311
H	-2.991290	-1.965135	7.609772
H	-1.574989	-0.879564	7.840232
H	-1.137257	-3.206887	1.883519
H	0.479138	-2.563795	2.246631
H	-2.215164	2.052533	0.202592
H	0.932586	-0.504652	1.570278
H	0.648893	-4.398740	3.478286
H	-1.058401	-4.879332	3.349025
H	-0.013443	-6.870938	8.645846
H	1.843735	-5.281084	5.125982
H	-6.342048	-0.142769	0.891656
H	-5.443616	-0.400629	2.410415
H	-5.085809	-1.398913	1.002697
H	-4.108697	2.828358	0.900909
H	-4.689174	2.106137	2.415571
H	-5.784980	2.250495	1.018890
H	-3.701859	1.343256	-1.171049
H	-5.406247	0.850058	-1.054824
H	-4.118312	-0.381151	-1.098609
H	0.884685	2.911840	2.273812

H	2.244867	3.084720	1.132599
H	1.974873	1.540542	1.974376
H	1.919216	0.337714	-0.312985
H	2.179517	1.896481	-1.127848
H	0.777459	0.881117	-1.562464
H	-0.698097	2.929897	-1.164982
H	0.728914	3.855845	-0.656640
H	-0.681856	3.748853	0.418310
H	-4.560652	-6.991428	7.869769
H	-3.249922	-7.820927	6.991188
H	-3.860381	-6.254262	6.401729
H	-1.588837	-6.539494	10.098215
H	-1.875327	-7.985719	9.092356
H	-3.227486	-7.199939	9.933655
H	-2.416261	-4.257359	9.324564
H	-4.046123	-4.977193	9.333634
H	-3.448271	-4.136040	7.877875
H	1.534660	-8.490513	8.367122
H	3.278499	-8.622740	8.045259
H	2.111168	-8.685296	6.697142
H	3.458928	-6.824346	5.566691
H	4.537930	-6.778499	6.976236
H	3.747513	-5.286856	6.422607
H	2.830884	-4.948321	8.790852
H	3.698708	-6.429924	9.276153
H	1.960664	-6.261965	9.613940
H	-6.730210	-1.658980	3.467994
H	-7.727847	-4.392622	3.511173
H	-8.726024	-3.501541	2.379470
H	-5.774191	-3.037346	2.112855
C	-6.136004	-5.088927	1.607442
H	-6.966816	-3.241783	0.829761
H	-9.711669	-2.071349	3.989676
H	-8.035946	0.423539	4.641290
H	-9.801147	0.283722	4.778756
H	-8.747526	-0.680521	5.841169
H	-10.022572	-0.249809	2.327868
H	-8.263168	-0.059284	2.164739
H	-9.054971	-1.534624	1.575889

H	-7.192539	-4.712319	6.605360
H	-5.263402	-3.227377	8.382273
H	-7.867976	-2.026495	7.453392
H	-5.432252	-4.807487	6.709998
H	-7.414731	-1.894132	9.151438
H	-6.597156	0.060130	8.118993
H	-5.189682	-0.932655	8.514716
H	-3.684849	0.268810	6.705000
H	-4.042491	-0.016628	4.194715
H	-5.363860	1.158262	4.300235
H	-3.693682	1.659494	4.640919
H	-4.593840	2.654591	6.667670
H	-6.236565	1.986792	6.602288
H	-5.209597	1.698643	8.028160
H	-6.465306	-0.515153	5.774127
H	-7.984832	-4.528244	8.940165
H	-6.424067	-5.369112	9.072863
H	-6.789207	-3.999663	10.149696
C	-4.888492	-5.100584	0.712620
H	-5.839410	-5.460145	2.600834
C	-7.215665	-6.002668	1.017913
C	-3.679786	-4.443020	1.340572
H	-4.578207	-6.140208	0.506174
H	-5.118841	-4.650784	-0.264106
O	-2.760166	-3.841770	0.579785
O	-3.466116	-4.484383	2.554280
C	-2.884743	-3.576503	-0.867176
C	-2.562502	-4.834649	-1.659105
H	-3.915465	-3.237076	-1.056726
C	-1.910618	-2.441433	-1.135986
H	-2.586370	-4.606979	-2.733899
H	-1.554128	-5.188643	-1.403063
H	-3.278015	-5.643328	-1.466187
H	-1.981628	-2.139534	-2.189897
H	-2.126415	-1.573417	-0.501053
H	-0.880293	-2.765732	-0.934036
H	-6.861556	-7.040527	0.928847
H	-8.113431	-6.009556	1.651502
H	-7.517369	-5.658557	0.015421

IV_M (in implicit toluene)

C	0.804724	-5.187469	6.025211
C	-0.453197	-4.851408	5.507470
C	-1.609681	-4.872734	6.333274
C	-1.473177	-5.350444	7.677999
C	-0.190166	-5.657152	8.139022
C	0.975634	-5.571561	7.355945
C	-0.588456	-4.476805	4.057486
N	-0.889548	-3.034374	3.802525
C	-0.920755	-2.836836	2.333773
C	-1.269312	-1.464729	1.806242
C	-2.433255	-0.770740	2.244389
C	-2.761378	0.479776	1.641363
C	-1.950716	0.950691	0.600003
C	-0.819367	0.268047	0.130313
C	-0.493666	-0.932244	0.775900
O	-3.185425	-1.275737	3.201646
Y	-3.490121	-2.684365	4.779438
O	-5.828477	-2.983148	3.991775
C	-6.886883	-3.575267	4.193926
O	-4.513534	-1.754034	6.345386
C	-5.331327	-0.977920	7.170559
C	-4.481640	-0.091969	8.120921
C	-5.320210	0.680973	9.149815
C	-3.986394	1.268705	2.133612
C	-4.178408	2.590080	1.368356
C	0.029304	0.758918	-1.051499
C	-0.470178	2.100002	-1.615329
O	-2.769265	-4.448167	5.848577
C	-2.708200	-5.552575	8.570824
C	-3.413332	-4.207505	8.849829
C	2.340471	-5.909945	7.973324
C	2.616712	-4.957634	9.159016
O	-1.674884	-1.351177	5.880872
C	-1.271329	-1.688161	7.217991
O	-7.985063	-3.398394	3.445164
C	-7.959989	-2.464053	2.301268

C	-9.047601	-1.409323	2.559361
C	-9.057361	-0.353780	1.443004
C	0.099433	-2.138917	4.438714
C	-0.597111	-0.922278	5.029011
C	-5.274022	0.430531	1.945944
C	-3.812979	1.613132	3.631930
C	1.497312	0.941618	-0.605320
C	-0.031785	-0.291504	-2.185214
C	-3.681497	-6.518633	7.856421
C	-2.354509	-6.180375	9.931973
C	2.335807	-7.369091	8.484489
C	3.489580	-5.760888	6.961810
C	-8.144796	-3.304656	1.036686
C	-6.853541	-3.973933	0.539679
C	-8.831331	-0.735315	3.921789
C	-7.109944	-4.589339	5.285079
C	-6.626338	-4.086947	6.657829
C	-6.982137	-5.111433	7.737029
C	-7.211336	-2.699161	6.983622
C	-6.349266	-1.852458	7.931976
C	-3.627795	0.883889	7.304344
H	0.877857	-1.819269	3.721917
H	0.599667	-2.688402	5.246876
H	0.108353	-0.298278	5.602902
H	-1.061825	-0.306702	4.249768
H	-0.568197	-2.535459	7.227790
H	-2.187045	-1.976468	7.740869
H	-0.822513	-0.808064	7.705287
H	-1.652813	-3.557485	1.951560
H	0.063637	-3.126773	1.908221
H	-2.219371	1.891621	0.126008
H	0.386368	-1.498654	0.457087
H	0.340577	-4.763632	3.521787
H	-1.420966	-5.038163	3.603799
H	-0.082933	-5.990693	9.170760
H	1.659842	-5.135288	5.348624
H	-6.137603	0.978378	2.354879
H	-5.194714	-0.533472	2.461504
H	-5.462960	0.247221	0.877069

H	-2.903073	2.212161	3.786472
H	-3.740719	0.707093	4.241870
H	-4.672951	2.201506	3.988808
H	-3.316098	3.262269	1.488983
H	-5.064050	3.111558	1.761166
H	-4.340647	2.424587	0.292317
H	1.565862	1.685538	0.202311
H	2.118617	1.287161	-1.446728
H	1.927034	0.000971	-0.233878
H	0.355041	-1.264972	-1.851776
H	0.566971	0.033892	-3.050672
H	-1.070315	-0.438982	-2.516756
H	-1.502591	2.025465	-1.987558
H	0.167045	2.408978	-2.457296
H	-0.434047	2.897255	-0.858278
H	-4.554234	-6.715325	8.496644
H	-3.183794	-7.478635	7.651462
H	-4.025119	-6.092914	6.906353
H	-1.691677	-5.533769	10.526235
H	-1.871497	-7.162735	9.819764
H	-3.278082	-6.326955	10.511802
H	-2.751534	-3.538991	9.421895
H	-4.319738	-4.374764	9.453399
H	-3.707157	-3.696701	7.925931
H	1.559809	-7.527741	9.246254
H	3.308029	-7.624369	8.934999
H	2.143664	-8.068355	7.657385
H	3.368801	-6.440958	6.105377
H	4.445888	-6.003799	7.448149
H	3.561093	-4.732186	6.578322
H	2.632440	-3.911774	8.818216
H	3.589545	-5.186329	9.622422
H	1.844393	-5.047148	9.936081
H	-6.976217	-1.969858	2.295094
H	-8.948189	-4.036935	1.212583
H	-8.504813	-2.638488	0.240736
H	-6.065984	-3.207938	0.593451
C	-6.402967	-5.246151	1.286306
H	-6.962040	-4.227920	-0.528278

H	-10.022704	-1.927657	2.568426
H	-7.823861	-0.294798	3.981221
H	-9.563967	0.069202	4.074232
H	-8.939477	-1.447572	4.748989
H	-9.787390	0.432874	1.678110
H	-8.070053	0.122362	1.348639
H	-9.328840	-0.772512	0.464884
H	-8.176446	-4.852599	5.313144
H	-5.532383	-3.987378	6.617798
H	-7.334053	-2.117421	6.056011
H	-6.549705	-5.496881	5.001275
H	-8.226896	-2.834719	7.393436
H	-7.004741	-1.198341	8.524676
H	-5.807170	-2.495643	8.645704
H	-3.812998	-0.784990	8.663357
H	-3.065163	0.355773	6.527258
H	-4.270702	1.630237	6.809873
H	-2.918568	1.427854	7.947748
H	-4.689606	1.393702	9.702889
H	-6.111119	1.262735	8.647747
H	-5.800185	0.022821	9.887095
H	-5.931570	-0.276211	6.539351
H	-8.075160	-5.225639	7.818726
H	-6.548865	-6.096680	7.517864
H	-6.601606	-4.790976	8.717016
C	-4.894419	-5.536867	1.056504
H	-6.520673	-5.092634	2.367070
C	-7.241053	-6.463945	0.886340
C	-4.067843	-4.428421	1.664659
H	-4.619886	-6.461747	1.580279
H	-4.695933	-5.679542	-0.013707
O	-3.659009	-3.385456	0.934178
O	-3.826078	-4.414191	2.873464
C	-3.612796	-3.352170	-0.540369
C	-2.263583	-3.902308	-0.975159
H	-4.431926	-3.967952	-0.936985
C	-3.823055	-1.899843	-0.925399
H	-2.191588	-3.890194	-2.071423
H	-1.459352	-3.275799	-0.565765
H	-2.125349	-4.936261	-0.628256
H	-3.806195	-1.806061	-2.020329
H	-4.790034	-1.534696	-0.556448
H	-3.031409	-1.268839	-0.501761
H	-6.958862	-7.352021	1.469419
H	-8.310724	-6.277110	1.056553
H	-7.105055	-6.696813	-0.181338

Structures of Figure 5

CM_2 (in implicit toluene)

C	1.068664	-0.541444	-2.755861
C	-0.073124	-1.088037	-1.925875
C	0.394614	-1.792533	-0.623747
C	0.680756	-0.757151	0.475892
C	1.385224	1.222888	-1.057887
C	1.720052	0.315771	0.128016
H	-0.784990	-0.288374	-1.666580
H	1.334011	-2.333868	-0.843685
H	-0.269945	-0.262580	0.747601
H	-0.593215	-1.791399	-2.583863
H	1.029638	-1.273197	1.381093
H	1.857083	0.960047	1.010729
H	2.700545	-0.147003	-0.075113
O	1.448428	-1.064272	-3.788396
O	1.719482	0.572536	-2.329108
C	-0.655941	-2.855984	-0.193465
H	-1.645175	-2.361505	-0.215280
C	-0.683021	-4.042854	-1.170300
H	-0.842448	-3.738329	-2.213502
H	0.270700	-4.592030	-1.127409
H	-1.486834	-4.744464	-0.905499
C	-0.425543	-3.388405	1.229453
H	-1.129707	-4.204540	1.446948
H	0.593846	-3.793769	1.334664
H	-0.567650	-2.618800	1.999292
H	0.308982	1.466348	-1.066421
C	2.196243	2.509957	-1.059249
H	3.271540	2.284745	-1.021699
H	1.992640	3.093560	-1.966472
H	1.934580	3.119495	-0.183564

I_{CM} (in implicit toluene)

C	-0.560459	-4.658869	5.664786
C	-0.924312	-3.777234	4.642995
C	-2.074730	-4.019087	3.846790
C	-2.796152	-5.237885	4.037820
C	-2.386145	-6.078712	5.077877
C	-1.291577	-5.819541	5.923021
C	-0.049983	-2.603083	4.297047
N	-0.609272	-1.242084	4.592410
C	0.294511	-0.246257	3.934953
C	-0.172965	1.182538	3.817771
C	-1.300872	1.476440	3.000253
C	-1.619571	2.844036	2.743212
C	-0.862837	3.830254	3.395528
C	0.207649	3.555966	4.258904
C	0.548184	2.207940	4.428365
O	-2.027151	0.467628	2.520932
Y	-3.001066	-1.106724	3.664959
O	-5.007056	-0.832192	3.223986
C	-6.351282	-0.634876	2.883491
C	-6.513961	-0.620616	7.646654
C	-7.882048	-1.142616	8.111138
C	-2.771425	3.224903	1.795659
C	-2.575077	2.534686	0.425707
C	1.001501	4.649607	4.988841
C	0.459838	6.059937	4.700215
O	-2.451505	-3.093180	2.957300
C	-3.960059	-5.621533	3.106607
C	-5.125074	-4.617302	3.242726
C	-0.923223	-6.816547	7.031589
C	-2.133387	-7.021868	7.968386
O	-2.646800	0.373474	5.605105
C	-3.212217	1.690602	5.706284
C	-0.733125	-1.055380	6.052221
C	-1.473649	0.220673	6.434324
C	-4.122026	2.789665	2.405951
C	-2.843342	4.742840	1.541651
C	0.919371	4.407639	6.513623
C	2.481232	4.601087	4.545274
C	-3.459891	-5.634183	1.643047
C	-4.522469	-7.020301	3.421014
C	-0.535102	-8.172921	6.400246
C	0.258503	-6.326276	7.886136
C	-7.031541	0.329968	3.874642

C	-7.419393	-0.238410	5.250686
C	-6.322269	0.838373	8.079155
H	0.262476	-1.030722	6.540730
H	-1.275475	-1.929484	6.436677
H	-1.787045	0.163971	7.491022
H	-0.840567	1.109360	6.298528
H	-3.448853	1.922952	6.756792
H	-4.132886	1.688781	5.112708
H	-2.513686	2.438503	5.298740
H	0.441226	-0.631862	2.914042
H	1.279095	-0.268139	4.441830
H	-1.127803	4.869987	3.222008
H	1.399847	1.931755	5.056390
H	0.931098	-2.707588	4.800099
H	0.127946	-2.613157	3.211370
H	-2.944054	-7.000560	5.241598
H	0.327065	-4.417192	6.252447
H	-4.945925	3.040226	1.719383
H	-4.304255	3.317022	3.354595
H	-4.150463	1.708868	2.589783
H	-1.910289	5.129392	1.105372
H	-3.056843	5.309483	2.460355
H	-3.656561	4.951006	0.830765
H	-1.623730	2.849891	-0.029228
H	-3.390854	2.816695	-0.258084
H	-2.566681	1.444725	0.533497
H	-0.126475	4.439107	6.853502
H	1.483698	5.179614	7.059936
H	1.335055	3.428450	6.790371
H	2.938592	3.627075	4.770081
H	3.066203	5.376427	5.064073
H	2.566406	4.770766	3.461936
H	0.524803	6.310587	3.631291
H	1.051722	6.803609	5.253782
H	-0.589055	6.165618	5.015475
H	-4.281975	-5.909997	0.963792
H	-2.652320	-6.371398	1.520113
H	-3.080398	-4.647527	1.352620
H	-4.945753	-7.076984	4.435442

H	-3.755791	-7.802913	3.322369
H	-5.330230	-7.253223	2.711803
H	-5.513304	-4.627034	4.270589
H	-5.951028	-4.898420	2.570669
H	-4.814926	-3.595715	2.994207
H	-1.361754	-8.590105	5.808084
H	-0.273920	-8.904604	7.180784
H	0.330990	-8.054143	5.733112
H	1.172874	-6.206063	7.287198
H	0.473699	-7.059373	8.677247
H	0.034821	-5.363753	8.370482
H	-2.442219	-6.061731	8.405825
H	-1.880108	-7.713902	8.786328
H	-2.994726	-7.442372	7.430601
H	-6.890968	-1.609300	2.934324
H	-6.348076	1.187450	4.001410
H	-7.950619	0.726089	3.411047
H	-8.184958	-1.022094	5.117997
C	-6.273088	-0.796085	6.122798
H	-7.920254	0.570847	5.805161
H	-5.734666	-1.211412	8.162605
H	-7.045421	1.502536	7.581968
H	-6.463494	0.950604	9.164282
H	-5.313187	1.194231	7.828819
H	-7.944213	-1.139451	9.208827
H	-8.697082	-0.509502	7.731330
H	-8.081173	-2.169383	7.769977
C	-5.996064	-2.281722	5.768178
H	-5.355478	-0.240945	5.870312
C	-6.458950	-0.130478	1.440495
C	-4.626934	-2.700472	6.235549
H	-6.799257	-2.926179	6.143337
H	-5.964633	-2.373144	4.677684
O	-4.427533	-3.502589	7.284032
O	-3.611857	-2.260815	5.688686
C	-5.517803	-4.219831	7.967630
C	-5.028726	-4.442086	9.388114
H	-6.405413	-3.572516	7.987934
C	-5.795758	-5.503755	7.202824

H	-5.808484	-4.951657	9.970245
H	-4.125531	-5.066231	9.393752
H	-4.800160	-3.484246	9.873905
H	-6.524886	-6.119135	7.747214
H	-6.198942	-5.293005	6.203343
H	-4.864213	-6.073057	7.082827
H	-6.000598	0.864448	1.352777
H	-5.917733	-0.814165	0.772021
H	-7.507119	-0.068890	1.109808

II$_{CM}$ (in implicit toluene)

C	2.412752	-1.506727	4.626542
C	1.816381	-1.528030	3.359636
C	0.482126	-1.084442	3.173380
C	-0.243320	-0.602124	4.307665
C	0.405772	-0.602477	5.545443
C	1.724548	-1.048271	5.750775
C	2.604331	-1.985898	2.165792
N	2.820388	-0.918734	1.119486
C	3.765550	-1.469805	0.092711
C	4.233168	-0.471591	-0.928377
C	3.279876	0.117378	-1.804092
C	3.735204	1.077794	-2.755166
C	5.099616	1.405229	-2.766928
C	6.048282	0.842552	-1.902382
C	5.576437	-0.104835	-0.984919
O	1.999116	-0.218459	-1.673653
Y	0.580496	-0.652372	-0.060202
C	2.749489	1.740920	-3.729316
C	2.103303	0.647009	-4.609143
C	7.536606	1.220950	-1.925612
C	7.854059	2.287881	-2.987414
O	-0.041453	-1.123795	1.954735
C	-1.697825	-0.125615	4.164881
C	-1.794692	1.023430	3.132952
C	2.336611	-1.005147	7.158616
C	2.343056	0.451766	7.675466
O	1.343212	1.504426	0.987118
C	0.706939	2.776931	0.799726
O	-0.369149	-2.312649	-0.910183
C	-1.325978	-3.305881	-0.618802
C	-5.748914	-1.837086	-0.360363
C	-6.926388	-1.174458	-1.093146
C	3.316794	0.342153	1.729286
C	2.785157	1.601441	1.058434
C	1.671851	2.515182	-2.933587
C	3.426756	2.750417	-4.673455
C	7.946590	1.780995	-0.544588
C	8.381284	-0.035390	-2.235889
C	-2.562612	-1.321091	3.706971
C	-2.280240	0.398188	5.490363
C	1.494882	-1.880520	8.114874
C	3.783507	-1.526709	7.179913
C	-2.747633	-2.803842	-0.959924
C	-3.214782	-1.743500	0.036438
C	-6.183374	-2.343279	1.022626
H	4.420096	0.378202	1.731513
H	2.975789	0.353526	2.771796
H	3.059003	2.471781	1.679056
H	3.187635	1.750987	0.044469
H	0.916409	3.433293	1.659418
H	-0.368929	2.587839	0.728122
H	1.057358	3.257482	-0.128082
H	3.241974	-2.310421	-0.396738
H	4.646389	-1.894081	0.610759
H	5.436943	2.144881	-3.488760
H	6.267125	-0.576177	-0.280175
H	3.587513	-2.366480	2.500659
H	2.092733	-2.818443	1.651000
H	-0.143597	-0.233997	6.411443
H	3.445441	-1.850585	4.707917
H	0.923723	2.946182	-3.618512
H	2.136114	3.343765	-2.378356
H	1.160540	1.867057	-2.213141
H	4.196174	2.274839	-5.298833
H	3.891601	3.582073	-4.123617
H	2.670221	3.180061	-5.347551

H	2.860355	0.187218	-5.261408
H	1.320539	1.080833	-5.252498
H	1.661611	-0.146260	-3.994952
H	7.357876	2.677583	-0.300004
H	9.012877	2.055535	-0.539584
H	7.785473	1.043571	0.254341
H	8.228061	-0.820393	-1.481813
H	9.454014	0.212830	-2.251442
H	8.108906	-0.453000	-3.216211
H	7.613012	1.937327	-4.001735
H	8.927594	2.525469	-2.963318
H	7.301655	3.221706	-2.804942
H	-3.613178	-1.013986	3.583308
H	-2.532403	-2.125977	4.456844
H	-2.195691	-1.716742	2.753593
H	-1.719905	1.264908	5.872467
H	-2.293614	-0.377202	6.270612
H	-3.319525	0.718123	5.325696
H	-1.138989	1.857794	3.423859
H	-2.827917	1.399111	3.082459
H	-1.501554	0.686574	2.133545
H	0.455469	-1.528774	8.173916
H	1.916967	-1.858426	9.131600
H	1.478425	-2.924475	7.769657
H	3.842484	-2.572952	6.845655
H	4.180313	-1.480706	8.204968
H	4.443232	-0.922257	6.539689
H	2.940445	1.094784	7.012335
H	2.775798	0.500292	8.686858
H	1.327343	0.869260	7.723410
H	-1.308490	-3.510978	0.474790
H	-2.729634	-2.397921	-1.985789
H	-3.442853	-3.659279	-0.966293
H	-2.396193	-1.026704	0.193279
C	-4.484513	-0.941753	-0.313736
H	-3.357904	-2.224256	1.016666
H	-5.462657	-2.716870	-0.962874
H	-6.530338	-1.514503	1.656614
H	-7.011917	-3.059902	0.925295
H	-5.363920	-2.852876	1.548986
H	-7.732953	-1.903721	-1.259987
H	-7.358108	-0.344610	-0.516171
H	-6.616956	-0.780746	-2.072793
C	-4.535869	0.236526	0.697506
H	-4.352651	-0.513253	-1.321885
C	-0.986010	-4.610019	-1.344571
C	-5.772995	1.096497	0.743971
H	-4.407373	-0.159852	1.712151
H	-3.677106	0.889388	0.482431
O	-5.872773	1.920952	-0.334533
O	-6.588732	1.085922	1.653055
C	-7.026616	2.832736	-0.353716
C	-7.311603	3.142033	-1.812990
H	-7.874432	2.296796	0.098286
C	-6.694552	4.066106	0.474380
H	-8.173719	3.819714	-1.882930
H	-6.448696	3.632798	-2.285792
H	-7.545851	2.226551	-2.371953
H	-7.544113	4.763769	0.467597
H	-6.485227	3.787088	1.514654
H	-5.816676	4.581196	0.057751
H	-1.664737	-5.422000	-1.040780
H	-1.061544	-4.482185	-2.433136
H	0.044311	-4.908674	-1.105784
C	-1.692198	0.848758	-1.933950
C	-1.135072	0.046107	-3.078092
C	-1.928238	-0.216004	-4.358642
C	-2.180409	1.071650	-5.164983
C	-3.430582	2.156917	-3.174164
C	-2.442196	2.323858	-4.322043
H	-0.154699	0.493356	-3.303102
H	-2.896835	-0.654308	-4.061692
H	-1.304813	1.270285	-5.804716
H	-0.887858	-0.913464	-2.590056
H	-3.026751	0.910332	-5.847581
H	-1.502260	2.719057	-3.904280
H	-2.834741	3.121622	-4.971784
O	-1.113017	0.798759	-0.828978

O	-2.791076	1.586018	-1.959342
C	-1.211333	-1.286602	-5.227768
H	-0.217817	-0.878235	-5.486431
C	-1.005202	-2.611591	-4.483072
H	-0.402677	-2.511086	-3.570443
H	-1.975984	-3.042451	-4.191198
H	-0.500852	-3.340567	-5.133745
C	-1.976057	-1.549371	-6.533732
H	-1.483005	-2.344614	-7.110651
H	-3.004380	-1.883557	-6.319214
H	-2.034877	-0.662905	-7.178573
H	-3.697571	3.154112	-2.794923
C	-4.710647	1.401403	-3.481779
H	-5.315585	1.319573	-2.573145
H	-5.282917	1.961444	-4.234868
H	-4.521150	0.395924	-3.873012

TS2$_{CM}$ (in implicit toluene)

C	1.345753	-3.784224	6.438838
C	0.299154	-3.771519	5.509523
C	-0.985454	-3.294711	5.869008
C	-1.220918	-2.881254	7.215435
C	-0.135712	-2.910551	8.097377
C	1.158554	-3.342672	7.750423
C	0.487812	-4.305177	4.118902
N	0.395877	-3.277339	3.023703
C	0.493818	-4.023800	1.724526
C	0.518893	-3.178462	0.483801
C	-0.631237	-2.407041	0.168233
C	-0.622371	-1.600987	-1.004323
C	0.540084	-1.610726	-1.792859
C	1.687263	-2.359644	-1.494836
C	1.646716	-3.142703	-0.333010
O	-1.663803	-2.435583	1.010290
Y	-1.935278	-2.090276	3.112235
C	-1.813381	-0.688567	-1.350196
C	-3.139062	-1.483328	-1.371218
C	2.949901	-2.343367	-2.369191
C	2.802653	-1.427249	-3.596081
O	-1.938469	-3.251586	4.935572
C	-2.627939	-2.459487	7.675187
C	-3.123213	-1.225070	6.885161
C	2.278251	-3.319935	8.801714
C	2.490523	-1.871173	9.296682
O	-0.107184	-0.464039	3.222681
C	-0.261495	0.968650	3.134686
O	-4.100550	-2.344619	2.979556
C	-4.994691	-3.426316	2.744688
C	-8.911831	-3.106294	5.092994
C	-10.150123	-2.252685	5.409661
C	1.479981	-2.280226	3.183147
C	1.140534	-0.911428	2.621466
C	-1.898107	0.432255	-0.288153
C	-1.654937	-0.016968	-2.727316
C	4.146981	-1.837716	-1.532035
C	3.248725	-3.774771	-2.870077
C	-3.604179	-3.638140	7.451535
C	-2.664547	-2.095218	9.170932
C	1.885044	-4.213403	9.999936
C	3.614182	-3.834200	8.238955
C	-6.373102	-3.150042	3.350880
C	-6.340057	-3.082361	4.877664
C	-9.082441	-4.528176	5.643155
H	2.421321	-2.631980	2.721219
H	1.656112	-2.165226	4.261851
H	1.939128	-0.204088	2.898767
H	1.026635	-0.910556	1.527224
H	0.554924	1.459855	3.685864
H	-1.228758	1.214854	3.580564
H	-0.251297	1.288414	2.081666
H	-0.380472	-4.696086	1.699314
H	1.400014	-4.658953	1.750265
H	0.551311	-0.992306	-2.686419
H	2.512436	-3.743616	-0.042812
H	1.462696	-4.824155	4.044063
H	-0.300570	-5.042228	3.899510
H	-0.299145	-2.581316	9.123001

H	2.317197	-4.155629	6.108027
H	-2.728803	1.117305	-0.520178
H	-0.966925	1.018775	-0.277291
H	-2.066990	0.023826	0.715346
H	-1.564247	-0.759291	-3.534396
H	-0.779940	0.648311	-2.769627
H	-2.544171	0.596482	-2.934249
H	-3.100536	-2.280552	-2.128984
H	-3.973124	-0.810809	-1.625442
H	-3.340241	-1.941714	-0.398087
H	3.958967	-0.817887	-1.164745
H	5.065456	-1.822311	-2.139299
H	4.330932	-2.482381	-0.661122
H	3.411046	-4.469853	-2.034364
H	4.154128	-3.783859	-3.496468
H	2.409949	-4.158103	-3.469429
H	1.980270	-1.751848	-4.250532
H	3.729477	-1.451285	-4.187414
H	2.620039	-0.382055	-3.305466
H	-4.615171	-3.363862	7.787794
H	-3.280821	-4.518370	8.026918
H	-3.655047	-3.906947	6.390193
H	-2.024158	-1.230052	9.400277
H	-2.352382	-2.936146	9.807596
H	-3.694271	-1.830871	9.453389
H	-2.391245	-0.404698	6.938087
H	-4.069678	-0.861274	7.313952
H	-3.314916	-1.461067	5.833373
H	0.957008	-3.866667	10.475481
H	2.677922	-4.206283	10.764672
H	1.729509	-5.252290	9.674205
H	3.536727	-4.876664	7.896739
H	4.385299	-3.796936	9.022574
H	3.964687	-3.219063	7.396835
H	2.776427	-1.214291	8.461839
H	3.289567	-1.834439	10.054022
H	1.577299	-1.462037	9.750376
H	-4.568337	-4.304455	3.274805
H	-6.741257	-2.196422	2.937347

H	-7.067767	-3.933132	3.006667
H	-5.457958	-2.496448	5.170849
C	-7.579703	-2.438165	5.524845
H	-6.185441	-4.096860	5.285241
H	-8.862373	-3.179210	3.993911
H	-9.216718	-4.517186	6.735429
H	-9.971709	-5.007058	5.207768
H	-8.214850	-5.163948	5.413806
H	-11.037481	-2.666771	4.907962
H	-10.371357	-2.229242	6.486000
H	-10.017506	-1.215385	5.069397
C	-7.350428	-2.405698	7.053429
H	-7.614753	-1.392995	5.174004
C	-5.068308	-3.761126	1.251869
C	-8.380788	-1.664960	7.870297
H	-7.277684	-3.422350	7.461033
H	-6.380063	-1.914736	7.234374
O	-8.489991	-0.379509	7.453994
O	-9.029315	-2.141138	8.788966
C	-9.481966	0.468604	8.120187
C	-9.861584	1.531844	7.102600
H	-10.347967	-0.166104	8.362150
C	-8.879369	1.035006	9.398603
H	-10.630876	2.194802	7.522593
H	-8.986257	2.140619	6.835297
H	-10.259720	1.070841	6.189155
H	-9.605830	1.689844	9.900620
H	-8.607536	0.225402	10.087391
H	-7.981383	1.625440	9.165364
H	-5.606643	-4.706823	1.088835
H	-5.593732	-2.972516	0.695566
H	-4.054011	-3.852086	0.838172
C	-4.305474	-0.353968	3.052067
C	-5.032531	-0.031557	1.749104
C	-6.443803	0.535135	1.968706
C	-6.408750	1.903574	2.705614
C	-5.079967	1.060983	4.775323
C	-5.243125	2.128505	3.685414
H	-4.397282	0.685398	1.207389

H	-6.979879	-0.180766	2.614113
H	-6.353209	2.705704	1.949448
H	-5.048471	-0.938745	1.141097
H	-7.363297	2.050303	3.231158
H	-4.299296	2.204133	3.124330
H	-5.372524	3.101153	4.185857
O	-3.061324	-0.110160	3.166085
O	-5.088448	-0.283194	4.177852
C	-7.244695	0.634224	0.645793
H	-6.725482	1.364067	-0.003901
C	-7.313509	-0.706059	-0.101662
H	-6.333870	-1.031824	-0.475886
H	-7.705606	-1.498338	0.555847
H	-7.983963	-0.631156	-0.970224
C	-8.670688	1.149662	0.899138
H	-9.241125	1.191002	-0.039913
H	-9.205638	0.473480	1.585787
H	-8.683720	2.155506	1.339246
H	-4.101548	1.192234	5.268581
C	-6.188744	1.055643	5.805831
H	-6.040605	0.250628	6.534051
H	-6.203367	2.012281	6.346739
H	-7.170139	0.902979	5.339614

III_{CM} (in implicit toluene)

C	5.282433	-1.491187	-2.442311
C	4.341808	-0.629577	-1.865644
C	3.227024	-0.175322	-2.608837
C	3.068192	-0.605681	-3.960227
C	4.045230	-1.456665	-4.485396
C	5.162379	-1.920012	-3.765737
C	4.522009	-0.142302	-0.455552
N	3.407418	-0.464812	0.506083
C	3.876666	-0.024534	1.858567
C	2.863853	-0.058747	2.968809
C	1.772932	0.851973	2.928767
C	0.871901	0.882303	4.035369
C	1.055778	-0.063228	5.056182
C	2.096271	-1.002711	5.084098
C	3.013045	-0.953033	4.028306
O	1.629786	1.634878	1.851767
Y	1.301421	0.872169	-0.167778
C	-0.244730	1.937198	4.127311
C	0.392193	3.344862	4.087037
C	2.253267	-2.049958	6.195964
C	1.149922	-1.944228	7.262117
O	2.344188	0.624581	-2.013326
C	1.838285	-0.181520	-4.779905
C	0.570928	-0.780318	-4.124681
C	6.172722	-2.856557	-4.445049
C	5.453096	-4.141017	-4.916138
O	0.731616	-1.311063	0.643346
C	-0.531725	-1.501298	1.320896
O	-2.014279	2.748348	-0.397467
C	-3.401031	2.334615	-0.395184
C	-4.202210	-1.127059	2.371649
C	-4.135650	-2.607198	2.775286
C	3.066423	-1.901974	0.445002
C	1.741296	-2.240275	1.108523
C	-1.243886	1.776610	2.964235
C	-1.051767	1.827660	5.434531
C	2.185930	-3.463427	5.572435
C	3.619412	-1.866063	6.894699
C	1.733356	1.360593	-4.837829
C	1.895652	-0.693325	-6.230693
C	6.794276	-2.143335	-5.667783
C	7.313647	-3.267576	-3.498802
C	-3.568990	0.997781	0.335402
C	-5.011635	0.573050	0.608359
C	-4.514745	-0.245894	3.587375
H	3.857429	-2.528537	0.903316
H	3.015313	-2.169333	-0.619934
H	1.438617	-3.261310	0.821553
H	1.794235	-2.175985	2.204198
H	-0.882405	-2.534218	1.175855
H	-1.233059	-0.793570	0.868567
H	-0.408437	-1.286027	2.394386

H	4.218855	1.013768	1.722949
H	4.757309	-0.632283	2.144405
H	0.348222	-0.060945	5.881100
H	3.863616	-1.640123	4.003695
H	5.469110	-0.547053	-0.052118
H	4.608232	0.958224	-0.445302
H	3.933365	-1.790157	-5.516776
H	6.121036	-1.817928	-1.825058
H	-2.056225	2.515032	3.055789
H	-1.698903	0.776063	2.993331
H	-0.779585	1.920406	1.983916
H	-0.417104	1.947917	6.324745
H	-1.582277	0.866766	5.512353
H	-1.810098	2.624505	5.456621
H	1.037199	3.499356	4.964728
H	-0.391720	4.118539	4.094466
H	0.997056	3.474171	3.183272
H	1.221140	-3.616606	5.066560
H	2.295582	-4.236112	6.349383
H	2.983900	-3.615838	4.831962
H	4.453958	-1.977344	6.188183
H	3.750479	-2.615918	7.690197
H	3.690691	-0.866204	7.346903
H	1.163420	-0.967161	7.766852
H	1.301768	-2.717764	8.029032
H	0.149548	-2.095461	6.829726
H	0.841304	1.655523	-5.413020
H	2.616153	1.787190	-5.336952
H	1.660355	1.793654	-3.833654
H	1.910035	-1.792217	-6.282138
H	2.776699	-0.309098	-6.766343
H	1.000614	-0.349930	-6.770128
H	0.625323	-1.879135	-4.125381
H	-0.326579	-0.482819	-4.689791
H	0.448081	-0.440121	-3.089877
H	6.028239	-1.863391	-6.403994
H	7.521108	-2.800464	-6.170130
H	7.315303	-1.226026	-5.356604
H	7.887856	-2.396043	-3.151451
H	8.009624	-3.936136	-4.026508
H	6.936415	-3.807042	-2.617380
H	5.001436	-4.667645	-4.062472
H	6.164808	-4.823385	-5.406284
H	4.653417	-3.916566	-5.635350
H	-3.759678	2.233363	-1.437052
H	-3.056054	0.219955	-0.247521
H	-3.016151	1.092333	1.277508
H	-5.601890	0.636705	-0.322082
C	-5.133301	-0.866950	1.155448
H	-5.486897	1.270864	1.317198
H	-3.191221	-0.856478	2.028349
H	-5.497968	-0.487547	4.017955
H	-3.764113	-0.402869	4.375730
H	-4.504220	0.823942	3.335643
H	-3.325251	-2.767192	3.501943
H	-5.067029	-2.946458	3.250776
H	-3.946392	-3.251398	1.904415
C	-6.625894	-1.158733	1.430005
H	-4.804478	-1.551918	0.355343
C	-4.139081	3.487641	0.272660
C	-6.999180	-2.603609	1.673116
H	-6.993488	-0.579071	2.286480
H	-7.206217	-0.835140	0.549376
O	-6.521931	-3.401728	0.687296
O	-7.660353	-3.006568	2.616996
C	-6.773338	-4.843816	0.806293
C	-5.651139	-5.520625	0.037546
H	-6.720486	-5.095635	1.876192
C	-8.161193	-5.159149	0.266111
H	-5.761015	-6.612226	0.100412
H	-5.677005	-5.229814	-1.022354
H	-4.672331	-5.245361	0.452525
H	-8.354937	-6.238792	0.337290
H	-8.928726	-4.629945	0.844548
H	-8.236934	-4.861438	-0.789809
H	-5.225379	3.330864	0.248398
H	-3.820303	3.581854	1.321316
H	-3.911983	4.428968	-0.245460

C	-1.091206	2.020668	-1.199646
C	-1.451414	1.991464	-2.689931
C	-1.858040	3.296522	-3.382312
C	-0.773148	4.383379	-3.243567
C	0.328148	4.220424	-0.916728
C	-0.636108	4.970548	-1.831250
H	-0.576611	1.561031	-3.194271
H	-2.775837	3.676992	-2.895487
H	0.193679	3.965241	-3.574149
H	-2.250121	1.242437	-2.773385
H	-1.003157	5.205677	-3.935082
H	-0.254067	6.002138	-1.890779
H	-1.624442	5.023780	-1.352570
O	-0.803675	0.784748	-0.744953
O	0.204066	2.768023	-1.009881
C	-2.203915	3.057021	-4.881582
H	-1.244613	2.936535	-5.418255
C	-3.036860	1.788959	-5.125334
H	-2.484121	0.869979	-4.889988
H	-3.955040	1.800414	-4.515776
H	-3.341224	1.729157	-6.180663
C	-2.946886	4.259917	-5.484605
H	-3.126208	4.110332	-6.559311
H	-3.927153	4.384904	-4.996472
H	-2.395257	5.203111	-5.372419
H	1.352692	4.388427	-1.291708
C	0.239951	4.653440	0.540329
H	-0.763349	4.446277	0.933263
H	0.965294	4.095128	1.146720
H	0.454041	5.728377	0.634811

TS3$_{CM}$ (in implicit toluene)

C	2.698278	-2.946475	1.657769
C	1.430146	-2.359283	1.570039
C	0.343767	-3.062683	0.995359
C	0.561571	-4.380304	0.487804
C	1.848556	-4.912788	0.601497
C	2.939601	-4.234962	1.177633
C	1.199208	-0.965586	2.079962
N	0.211353	-0.850036	3.213220
C	0.213569	0.587331	3.645757
C	-0.611123	0.904264	4.860250
C	-2.017366	0.708166	4.801925
C	-2.800513	1.003158	5.954346
C	-2.132860	1.470566	7.097753
C	-0.746203	1.663227	7.175735
C	-0.002828	1.365345	6.026184
O	-2.539621	0.208426	3.683539
Y	-2.139744	-1.397404	2.296072
C	-4.320717	0.766958	5.961020
C	-4.991696	1.550707	4.811521
C	-0.038936	2.170711	8.441518
C	-1.018772	2.425633	9.599901
O	-0.849133	-2.476820	0.954992
C	-0.593446	-5.178924	-0.140560
C	-1.685792	-5.435049	0.925416
C	4.309367	-4.924848	1.262091
C	4.185121	-6.213637	2.107058
O	-1.601510	-2.835204	4.197502
C	-2.521218	-3.727887	4.855960
O	-5.808259	-1.709942	1.542355
C	-6.896277	-2.367165	2.283733
C	-6.050911	-4.099968	6.418391
C	-5.104636	-5.102760	7.095361
C	0.566768	-1.776002	4.315818
C	-0.627167	-2.274068	5.115750
C	-4.600458	-0.741710	5.784264
C	-4.981869	1.207703	7.279746
C	0.994171	1.120109	8.909007
C	0.691005	3.496318	8.126795
C	-1.184154	-4.394085	-1.336166
C	-0.137808	-6.550405	-0.671514
C	4.793020	-5.293312	-0.158937
C	5.374998	-4.026267	1.912589
C	-6.410894	-3.187818	3.479696
C	-7.537017	-3.755335	4.349661
C	-6.760969	-3.232857	7.466385

H	1.290313	-1.313410	5.012252
H	1.050213	-2.648489	3.857197
H	-0.284753	-3.069059	5.798906
H	-1.110919	-1.482291	5.706762
H	-1.975297	-4.598944	5.249749
H	-3.253498	-4.042083	4.107086
H	-3.035816	-3.210036	5.679227
H	-0.150450	1.167026	2.778987
H	1.259307	0.898852	3.829747
H	-2.730289	1.688884	7.979049
H	1.083529	1.489020	6.026698
H	2.164605	-0.529651	2.399587
H	0.804679	-0.324010	1.272963
H	2.021077	-5.920563	0.224691
H	3.498293	-2.364654	2.118878
H	-5.684565	-0.932042	5.794588
H	-4.152130	-1.316632	6.608571
H	-4.195239	-1.109745	4.834421
H	-4.833085	2.280077	7.473959
H	-4.602325	0.642910	8.144185
H	-6.065083	1.026768	7.217576
H	-4.819658	2.631164	4.929015
H	-6.079412	1.376008	4.820070
H	-4.595764	1.236392	3.841289
H	0.497897	0.165921	9.140030
H	1.514927	1.466784	9.815291
H	1.753281	0.928379	8.137883
H	1.440768	3.364600	7.333975
H	1.208849	3.874288	9.021924
H	-0.023473	4.262274	7.792066
H	-1.764301	3.191358	9.341205
H	-0.464718	2.783536	10.480099
H	-1.551848	1.508585	9.892289
H	-2.047002	-4.934934	-1.757091
H	-0.430779	-4.284303	-2.130639
H	-1.511709	-3.394007	-1.031192
H	0.252718	-7.195432	0.129925
H	0.636354	-6.452732	-1.447071
H	-0.997769	-7.067431	-1.122216

H	-1.276180	-6.032702	1.753570
H	-2.524611	-5.994643	0.481426
H	-2.082925	-4.501374	1.337683
H	4.091133	-5.973314	-0.661730
H	5.773151	-5.793307	-0.115871
H	4.892069	-4.391162	-0.780069
H	5.529401	-3.099968	1.339846
H	6.336441	-4.559211	1.950999
H	5.103576	-3.753326	2.943144
H	3.845466	-5.977478	3.126540
H	5.157536	-6.725494	2.176102
H	3.464430	-6.917442	1.667199
H	-7.414330	-3.033150	1.567540
H	-5.793020	-4.022776	3.118889
H	-5.747131	-2.546850	4.070728
H	-8.276964	-4.264210	3.707637
C	-7.023376	-4.762470	5.401894
H	-8.079716	-2.941048	4.855891
H	-5.409738	-3.431603	5.823537
H	-7.331126	-3.850829	8.175616
H	-6.026886	-2.651965	8.042833
H	-7.454726	-2.514614	7.004606
H	-4.327676	-4.567973	7.662324
H	-5.630358	-5.754455	7.807346
H	-4.604359	-5.743614	6.354348
C	-8.236522	-5.467917	6.052566
H	-6.450553	-5.536470	4.862964
C	-7.806574	-1.211358	2.669628
C	-7.877463	-6.576898	7.014000
H	-8.865995	-4.750062	6.593544
H	-8.846709	-5.912316	5.250412
O	-7.194187	-7.558720	6.375685
O	-8.147572	-6.594376	8.204236
C	-6.687016	-8.670797	7.191529
C	-5.448054	-9.176240	6.472010
H	-6.423542	-8.257720	8.176759
C	-7.780246	-9.718758	7.344476
H	-4.995374	-9.999354	7.041978
H	-5.705510	-9.548194	5.469987

H	-4.705137	-8.374189	6.368306
H	-7.409400	-10.563032	7.942878
H	-8.654518	-9.291433	7.851432
H	-8.086158	-10.098892	6.358998
H	-8.750849	-1.579813	3.090859
H	-7.306356	-0.575614	3.415010
H	-8.035379	-0.600054	1.786917
C	-4.632364	-2.370598	1.287943
C	-4.570311	-3.211560	0.033925
C	-5.316899	-2.764265	-1.225425
C	-4.780495	-1.432003	-1.794437
C	-4.095639	0.272451	0.005804
C	-5.162022	-0.168646	-1.004026
H	-3.507926	-3.388717	-0.179781
H	-6.379849	-2.620562	-0.954526
H	-3.682236	-1.506045	-1.870112
H	-4.975800	-4.177477	0.383299
H	-5.151478	-1.323805	-2.823214
H	-5.326447	0.673899	-1.694429
H	-6.112367	-0.334127	-0.474473
O	-3.915712	-2.755735	2.278185
O	-3.557172	-0.839444	0.715621
C	-5.262460	-3.866885	-2.328495
H	-4.271648	-3.784621	-2.812364
C	-5.396227	-5.302797	-1.798674
H	-4.546092	-5.602163	-1.170279
H	-6.318629	-5.422723	-1.207488
H	-5.447950	-6.012900	-2.637358
C	-6.344519	-3.630568	-3.394422
H	-6.238812	-4.342231	-4.226389
H	-7.345354	-3.773450	-2.956076
H	-6.308636	-2.618623	-3.819770
H	-3.249196	0.696341	-0.573676
C	-4.619711	1.334430	0.971320
H	-5.436515	0.909883	1.570923
H	-3.823454	1.653201	1.658664
H	-4.994316	2.215721	0.428138

IV$_{CM}$ (in implicit toluene)

C	4.813411	-1.536186	-2.262034
C	3.921711	-0.546819	-1.828260
C	2.773037	-0.216083	-2.592872
C	2.542933	-0.918769	-3.817881
C	3.469089	-1.892335	-4.200116
C	4.614706	-2.230278	-3.456888
C	4.168894	0.167772	-0.529366
N	3.098953	-0.034115	0.514544
C	3.578731	0.611242	1.781904
C	2.742067	0.374517	3.010426
C	1.385358	0.801572	3.024463
C	0.632398	0.649220	4.224385
C	1.258796	0.053457	5.328995
C	2.586157	-0.399122	5.324325
C	3.308369	-0.220968	4.136504
O	0.862467	1.304072	1.914600
Y	1.100111	1.332166	-0.241310
C	-0.825169	1.129061	4.284750
C	-0.877650	2.647863	4.000262
C	3.253877	-1.059051	6.540282
C	2.308874	-1.150483	7.750717
O	1.949974	0.723940	-2.147543
C	1.303930	-0.606744	-4.672018
C	0.026967	-0.908115	-3.850733
C	5.563961	-3.322626	-3.970653
C	4.791285	-4.653789	-4.114008
O	0.515391	-1.126850	-0.040604
C	-0.819782	-1.645153	-0.073836
O	-3.047539	2.537740	-1.461663
C	-3.680150	2.253867	-0.141158
C	-4.212490	-1.795177	1.980136
C	-3.908802	-3.256297	1.618623
C	2.785124	-1.474225	0.692819
C	1.318851	-1.752194	0.989989
C	-1.660735	0.364701	3.235114
C	-1.476204	0.884597	5.657258
C	3.699046	-2.491469	6.167491

C	4.492851	-0.235663	6.960242
C	1.341043	0.877140	-5.105933
C	1.234186	-1.457565	-5.953307
C	6.127011	-2.911420	-5.350164
C	6.749625	-3.557400	-3.019719
C	-3.850371	0.745506	0.051405
C	-5.052119	0.386515	0.934679
C	-4.556930	-1.661972	3.468903
H	3.400586	-1.920357	1.493850
H	3.035525	-1.979818	-0.248791
H	1.155357	-2.843316	0.961261
H	1.001760	-1.371994	1.972728
H	-0.807445	-2.718607	-0.321785
H	-1.360034	-1.093446	-0.849980
H	-1.321251	-1.500755	0.897344
H	3.641030	1.694134	1.571325
H	4.609683	0.263689	1.984711
H	0.677153	-0.065073	6.239993
H	4.350800	-0.545694	4.076999
H	5.141850	-0.157657	-0.115279
H	4.234346	1.258190	-0.689517
H	3.293853	-2.430178	-5.131834
H	5.673895	-1.756289	-1.627982
H	-2.709239	0.696448	3.260212
H	-1.642695	-0.714296	3.448078
H	-1.265086	0.528814	2.226522
H	-0.949791	1.418666	6.461864
H	-1.507752	-0.184630	5.913897
H	-2.513068	1.251361	5.636457
H	-0.329958	3.199830	4.778232
H	-1.920350	3.002157	3.999436
H	-0.425465	2.881291	3.029457
H	2.832843	-3.101607	5.871510
H	4.190074	-2.980191	7.023555
H	4.409879	-2.486685	5.328881
H	5.233591	-0.175179	6.150044
H	4.984013	-0.695162	7.832191
H	4.203058	0.791119	7.227398
H	1.979457	-0.156043	8.086023
H	2.830355	-1.630703	8.591700
H	1.415436	-1.751680	7.525852
H	0.432475	1.134224	-5.672909
H	2.209036	1.060103	-5.756080
H	1.420617	1.542661	-4.238538
H	1.172862	-2.533179	-5.730694
H	2.102387	-1.287087	-6.606405
H	0.332415	-1.183548	-6.521042
H	-0.025649	-1.980841	-3.612379
H	-0.873227	-0.640146	-4.426768
H	0.021317	-0.351236	-2.907472
H	5.324939	-2.769183	-6.087951
H	6.807387	-3.686593	-5.736209
H	6.685877	-1.967194	-5.274907
H	7.361508	-2.650812	-2.902127
H	7.398692	-4.348531	-3.423465
H	6.413485	-3.878643	-2.022716
H	4.383355	-4.971440	-3.143112
H	5.456088	-5.448590	-4.486579
H	3.951445	-4.560848	-4.817058
H	-4.667787	2.709660	-0.300445
H	-3.999942	0.288742	-0.940545
H	-2.924614	0.326887	0.463478
H	-5.956280	0.852264	0.508392
C	-5.265052	-1.139288	1.041829
H	-4.930832	0.815696	1.943364
H	-3.279081	-1.234361	1.814050
H	-5.415036	-2.294388	3.738349
H	-3.702831	-1.973231	4.086708
H	-4.795952	-0.624601	3.745364
H	-3.053639	-3.625051	2.204587
H	-4.757876	-3.919824	1.833723
H	-3.659923	-3.359194	0.551896
C	-6.732873	-1.432012	1.431693
H	-5.116703	-1.562176	0.033149
C	-2.958825	2.977172	0.983165
C	-7.075200	-2.904100	1.464358
H	-6.973076	-1.009784	2.415540
H	-7.389975	-0.955114	0.688177

O	-6.942643	-3.450346	0.230529
O	-7.407509	-3.530545	2.457173
C	-7.141189	-4.902294	0.110836
C	-6.277888	-5.340725	-1.059516
H	-6.786431	-5.356819	1.048107
C	-8.624064	-5.190760	-0.073970
H	-6.356443	-6.428535	-1.193691
H	-6.605252	-4.852417	-1.988535
H	-5.224154	-5.087609	-0.881207
H	-8.785884	-6.273573	-0.171517
H	-9.196356	-4.831524	0.790786
H	-8.999645	-4.699213	-0.983014
H	-3.532265	2.850627	1.911549
H	-1.955523	2.570534	1.150440
H	-2.882548	4.052029	0.774984
C	-1.833933	2.089483	-1.746177
C	-1.426980	2.360733	-3.164231
C	-1.192078	3.846677	-3.543672
C	0.023919	4.395263	-2.768713
C	0.980622	4.708041	-0.421619
C	-0.256523	4.842942	-1.329502
H	-0.514609	1.784795	-3.357210
H	-2.099659	4.414380	-3.267177
H	0.795324	3.607081	-2.745554
H	-2.235324	1.943639	-3.781130
H	0.469392	5.237390	-3.316908
H	-0.635928	5.878222	-1.317644
H	-1.045794	4.224723	-0.870851
O	-1.142085	1.452277	-0.932336
O	1.392266	3.370126	-0.399140
C	-0.990317	4.003058	-5.077886
H	0.032235	3.652628	-5.305701
C	-1.970585	3.181780	-5.927066
H	-1.811277	2.098588	-5.831144
H	-3.014049	3.401240	-5.647951
H	-1.852801	3.431313	-6.991481
C	-1.099575	5.482551	-5.480874
H	-0.855732	5.615255	-6.544773
H	-2.128083	5.845416	-5.323922
H	-0.425305	6.129392	-4.903708
H	1.792941	5.336307	-0.850590
C	0.681034	5.201252	0.997154
H	-0.147657	4.615341	1.423807
H	1.558864	5.055992	1.641019
H	0.403937	6.266872	1.007968

Structures of Figure S59

$CM_{2,2}$ (in implicit toluene)

C	0.912845	-0.556973	-2.790619
C	-0.155062	-1.196443	-1.923187
C	0.353363	-1.843798	-0.611555
C	0.597301	-0.771009	0.459817
C	1.379724	1.217552	-1.078186
C	1.658121	0.272445	0.092794
H	-0.932880	-0.458283	-1.679316
H	1.318433	-2.343297	-0.821632
H	-0.360634	-0.276913	0.701213
H	-0.616568	-1.948911	-2.570397
H	0.932744	-1.252335	1.388423
H	1.834930	0.915517	0.970242
H	2.614244	-0.236200	-0.114413
O	1.230978	-1.022048	-3.871775
O	1.553611	0.570133	-2.385830
C	-0.639006	-2.950366	-0.151633
H	-1.652836	-2.508437	-0.169487
C	-0.616060	-4.155499	-1.106345
H	-0.822705	-3.883107	-2.150121
H	0.371446	-4.642822	-1.079148
H	-1.366490	-4.900984	-0.806735
C	-0.363523	-3.445409	1.276929
H	-1.011784	-4.301733	1.513178
H	0.680689	-3.783165	1.378231
H	-0.547710	-2.675060	2.036979
H	2.210762	1.937953	-1.105566
C	0.083566	2.018604	-1.003559
H	0.111357	2.663815	-0.113845
H	-0.013055	2.657476	-1.891195
H	-0.811274	1.389562	-0.929509

$I_{CM,2}$ (in implicit toluene)

C	-0.537519	-4.670508	5.671573
C	-0.914039	-3.795673	4.648256
C	-2.072110	-4.045514	3.865326
C	-2.783991	-5.268265	4.067815
C	-2.361921	-6.101211	5.109384
C	-1.262037	-5.831812	5.944234
C	-0.045206	-2.620488	4.290910
N	-0.600197	-1.259563	4.595048
C	0.301090	-0.260287	3.939188
C	-0.180132	1.163186	3.813095
C	-1.307688	1.438275	2.988819
C	-1.646718	2.800172	2.727127
C	-0.903899	3.799479	3.376030
C	0.169926	3.543264	4.241381
C	0.526175	2.200459	4.420456
O	-2.013539	0.415451	2.507739
Y	-2.996163	-1.129865	3.683822
O	-4.995395	-0.748766	3.269171
C	-6.270396	-0.301475	2.893844
C	-6.505119	-0.691708	7.700631
C	-7.870387	-1.231661	8.151928
C	-2.796763	3.158722	1.768107
C	-2.557351	2.484800	0.396733
C	0.952349	4.649712	4.964290
C	0.402758	6.053111	4.658276
O	-2.465504	-3.123824	2.978412
C	-3.951203	-5.664700	3.146196
C	-5.119880	-4.665253	3.279980
C	-0.883144	-6.816969	7.059712
C	-2.085359	-7.011517	8.009074
O	-2.632240	0.350081	5.612913
C	-3.199933	1.666506	5.713925
C	-0.717717	-1.077012	6.055932
C	-1.458965	0.197582	6.441864
C	-4.146630	2.683117	2.350871
C	-2.904323	4.676057	1.524920
C	0.864410	4.421677	6.490886
C	2.434517	4.606698	4.528196
C	-3.458087	-5.684778	1.680226
C	-4.504233	-7.064102	3.473067
C	-0.500574	-8.180146	6.440079
C	0.306093	-6.317206	7.898010
C	-6.956576	0.485954	4.029086

C	-7.392514	-0.262319	5.301794
C	-6.326837	0.763664	8.150868
H	0.279934	-1.051407	6.540128
H	-1.256948	-1.952339	6.441521
H	-1.771821	0.139198	7.498611
H	-0.827277	1.087349	6.306944
H	-3.456464	1.890610	6.761715
H	-4.108652	1.667815	5.102640
H	-2.494364	2.417168	5.323877
H	0.457423	-0.648021	2.920615
H	1.282184	-0.271890	4.453017
H	-1.181470	4.835123	3.196832
H	1.379905	1.938867	5.052109
H	0.942105	-2.725689	4.781490
H	0.118619	-2.629395	3.203016
H	-2.913975	-7.025034	5.282530
H	0.354957	-4.422609	6.249187
H	-4.963304	2.906394	1.646609
H	-4.364966	3.206666	3.293932
H	-4.145331	1.603497	2.539753
H	-1.978379	5.089694	1.098468
H	-3.138305	5.229181	2.446990
H	-3.717173	4.870082	0.809692
H	-1.609336	2.832843	-0.040595
H	-3.370388	2.745805	-0.298875
H	-2.515490	1.394661	0.497745
H	-0.183212	4.450327	6.825486
H	1.421927	5.201643	7.032744
H	1.284274	3.447353	6.778387
H	2.897814	3.638491	4.765320
H	3.011733	5.391249	5.041608
H	2.523890	4.765712	3.443580
H	0.469620	6.291638	3.586689
H	0.988242	6.806847	5.204939
H	-0.647685	6.155695	4.969195
H	-4.281783	-5.970165	1.006835
H	-2.646823	-6.418056	1.558276
H	-3.085589	-4.698016	1.381034
H	-4.921114	-7.116094	4.490268

H	-3.734357	-7.843594	3.374885
H	-5.314932	-7.305848	2.770243
H	-5.505067	-4.666909	4.308932
H	-5.947537	-4.952305	2.612711
H	-4.815366	-3.645191	3.021747
H	-1.331976	-8.603853	5.859345
H	-0.233072	-8.903333	7.226330
H	0.360160	-8.068973	5.764776
H	1.214660	-6.202466	7.289280
H	0.529580	-7.042214	8.694229
H	0.086249	-5.350195	8.375108
H	-2.391306	-6.046176	8.436974
H	-1.825190	-7.693562	8.833260
H	-2.950566	-7.438927	7.483174
H	-6.159183	0.424271	2.059485
H	-6.268852	1.299998	4.315001
H	-7.849807	0.974109	3.601901
H	-8.108130	-1.061531	5.049387
C	-6.255728	-0.841563	6.176495
H	-7.967254	0.457246	5.905256
H	-5.722480	-1.282356	8.211690
H	-7.051995	1.427402	7.655944
H	-6.474936	0.863491	9.236268
H	-5.318914	1.129892	7.909587
H	-7.934965	-1.250550	9.249256
H	-8.689344	-0.597198	7.783075
H	-8.061883	-2.252672	7.789534
C	-5.970782	-2.316506	5.790050
H	-5.336135	-0.282030	5.942403
C	-7.121721	-1.462633	2.362317
C	-4.604101	-2.740488	6.253923
H	-6.775622	-2.976265	6.134220
H	-5.923820	-2.366702	4.696422
O	-4.401517	-3.552400	7.293641
O	-3.591179	-2.294016	5.707193
C	-5.493057	-4.261077	7.984423
C	-5.005996	-4.468357	9.407756
H	-6.380020	-3.613118	7.996052
C	-5.772578	-5.552639	7.233833

H	-5.787959	-4.968426	9.994981
H	-4.104894	-5.095249	9.421469
H	-4.774461	-3.505869	9.882731
H	-6.503225	-6.160034	7.785068
H	-6.174855	-5.352405	6.231973
H	-4.842351	-6.125139	7.120620
H	-6.591252	-1.955884	1.536481
H	-7.303617	-2.220959	3.137909
H	-8.096798	-1.107726	1.994703

$II_{CM,2}$ (in implicit toluene)

C	1.777216	-4.786837	5.200393
C	0.845348	-4.043157	4.466276
C	-0.515760	-4.435535	4.420506
C	-0.924052	-5.616724	5.112255
C	0.051994	-6.318900	5.825032
C	1.405987	-5.938754	5.897923
C	1.269715	-2.827911	3.688292
N	0.670487	-1.523913	4.144097
C	1.251858	-0.447618	3.277417
C	0.814605	0.957543	3.587429
C	-0.547126	1.309825	3.370942
C	-0.963257	2.647314	3.633769
C	-0.006636	3.545019	4.132933
C	1.334132	3.209316	4.375891
C	1.720212	1.894148	4.081767
O	-1.389678	0.371871	2.959192
Y	-1.931719	-1.609664	3.665677
C	-2.425781	3.071876	3.403955
C	-2.851291	2.756304	1.950426
C	2.360233	4.206885	4.935636
C	1.750583	5.596144	5.190414
O	-1.373996	-3.685813	3.743326
C	-2.394918	-6.067952	5.080619
C	-3.280343	-5.012969	5.786322
C	2.391157	-6.781553	6.722228
C	1.932501	-6.817240	8.197793
O	-1.394537	-0.914356	5.965132
C	-2.396079	-0.352143	6.834669
O	-3.868070	-1.611119	4.399826
C	-5.244754	-1.846972	4.428299
C	-6.559653	-5.598412	2.203545
C	-6.857642	-6.558317	1.044525
C	0.947271	-1.303508	5.579353
C	-0.072655	-0.407322	6.262010
C	-3.350643	2.310523	4.381138
C	-2.639571	4.578983	3.636447
C	2.915825	3.673732	6.276209
C	3.524803	4.370173	3.932487
C	-2.867263	-6.248313	3.618152
C	-2.607126	-7.411236	5.802258
C	2.430591	-8.223927	6.167882
C	3.819899	-6.213720	6.679771
C	-5.581155	-2.918755	3.375638
C	-7.057277	-3.283561	3.237536
C	-6.747662	-6.311995	3.548768
H	1.958903	-0.881428	5.738328
H	0.915506	-2.288142	6.065224
H	0.090716	-0.447954	7.352742
H	-0.010819	0.640403	5.931528
H	-2.216773	-0.682415	7.870475
H	-3.359810	-0.717633	6.465308
H	-2.369071	0.747624	6.783597
H	0.961371	-0.704406	2.244591
H	2.357106	-0.504096	3.334283
H	-0.326728	4.561872	4.345719
H	2.753380	1.574846	4.245632
H	2.373954	-2.744807	3.712468
H	0.972065	-2.937334	2.632040
H	-0.251012	-7.216693	6.363564
H	2.810949	-4.437216	5.213567
H	-4.399442	2.610571	4.227634
H	-3.081263	2.543525	5.422457
H	-3.289835	1.225546	4.235347
H	-2.015468	5.189324	2.966627
H	-2.422746	4.873556	4.674084
H	-3.691508	4.830304	3.435261

H	-2.217042	3.301797	1.235284
H	-3.895526	3.070528	1.788594
H	-2.771112	1.682446	1.749741
H	2.105864	3.555950	7.011189
H	3.661577	4.370015	6.691196
H	3.401747	2.695687	6.151073
H	4.032901	3.413755	3.744575
H	4.273114	5.079247	4.320158
H	3.154550	4.749863	2.969171
H	1.361947	6.046157	4.265205
H	2.522075	6.270037	5.591443
H	0.930629	5.553909	5.922891
H	-3.912514	-6.592311	3.604875
H	-2.253278	-7.003908	3.105233
H	-2.798021	-5.306984	3.061663
H	-2.349935	-7.352013	6.870047
H	-2.014184	-8.218903	5.347090
H	-3.667828	-7.695307	5.733394
H	-2.975118	-4.896037	6.837146
H	-4.333812	-5.335855	5.771823
H	-3.219523	-4.033661	5.296655
H	1.442560	-8.702736	6.214576
H	3.132420	-8.842269	6.749194
H	2.755956	-8.225359	5.117496
H	4.217306	-6.187417	5.654273
H	4.489191	-6.846801	7.280657
H	3.863874	-5.195336	7.093382
H	1.900398	-5.801385	8.618491
H	2.625593	-7.420940	8.804576
H	0.929453	-7.254707	8.297270
H	-5.536581	-2.250724	5.424410
H	-5.198775	-2.549677	2.409777
H	-4.975986	-3.804545	3.621225
H	-7.640564	-2.368454	3.041117
C	-7.356096	-4.271102	2.094115
H	-7.439501	-3.681344	4.193292
H	-5.494827	-5.319938	2.125237
H	-7.805614	-6.561044	3.730337
H	-6.186180	-7.256674	3.563309

H	-6.393959	-5.705264	4.393501
H	-6.135354	-7.387330	1.033219
H	-7.858909	-7.006530	1.134115
H	-6.808740	-6.051081	0.071301
C	-8.881870	-4.486922	1.975987
H	-7.043305	-3.786551	1.153832
C	-5.992124	-0.526766	4.210263
C	-9.330411	-4.790054	0.556598
H	-9.227829	-5.224722	2.708050
H	-9.391336	-3.541838	2.225973
O	-10.198052	-5.803801	0.299866
O	-8.951567	-4.121956	-0.390555
C	-10.739486	-6.690859	1.333541
C	-11.100254	-7.977533	0.608106
H	-9.948247	-6.902995	2.069319
C	-11.930690	-6.024457	2.008081
H	-11.501833	-8.710185	1.321614
H	-11.860847	-7.782986	-0.160368
H	-10.214874	-8.409205	0.123414
H	-12.363006	-6.696855	2.762529
H	-11.638337	-5.091019	2.507353
H	-12.703047	-5.792919	1.261769
H	-7.083441	-0.635596	4.296797
H	-5.655366	0.210184	4.951965
H	-5.746130	-0.130724	3.213646
C	-3.361771	-1.563213	0.641991
C	-3.611629	-2.188404	-0.700659
C	-4.982995	-2.903494	-0.822861
C	-6.121597	-1.896390	-1.036895
C	-5.176568	0.123868	0.362751
C	-6.304312	-0.869420	0.087165
H	-3.533159	-1.427473	-1.489936
H	-5.176556	-3.441550	0.123975
H	-5.972792	-1.382239	-2.002266
H	-2.792679	-2.900133	-0.845550
H	-7.069377	-2.446039	-1.116196
H	-7.196018	-0.262182	-0.136566
H	-6.524934	-1.390876	1.031905
O	-2.511074	-2.041082	1.405947

O	-4.043001	-0.510901	1.076113
C	-4.877111	-3.976242	-1.946066
H	-4.334194	-3.508820	-2.788703
C	-4.064719	-5.186813	-1.459744
H	-3.078543	-4.908924	-1.062684
H	-4.606635	-5.710588	-0.658029
H	-3.902309	-5.902984	-2.277664
C	-6.239740	-4.445272	-2.474883
H	-6.101725	-5.285696	-3.170219
H	-6.898392	-4.782587	-1.661751
H	-6.773984	-3.652458	-3.014791
H	-5.521855	0.809021	1.149584
C	-4.700621	0.966661	-0.811841
H	-5.528534	1.613000	-1.135045
H	-3.865448	1.606788	-0.501417
H	-4.389130	0.369821	-1.676389

TS2$_{CM,2}$ (in implicit toluene)

C	1.410676	-3.314421	6.672176
C	0.402099	-3.440343	5.709646
C	-0.934656	-3.073400	5.996458
C	-1.266103	-2.699049	7.334216
C	-0.218061	-2.581052	8.252423
C	1.131321	-2.852712	7.959239
C	0.688612	-4.044785	4.369464
N	0.556394	-3.116373	3.197502
C	0.806233	-3.954422	1.982044
C	0.734885	-3.260386	0.654675
C	-0.519967	-2.767674	0.205553
C	-0.597205	-2.222733	-1.110019
C	0.593423	-2.096302	-1.843333
C	1.849514	-2.515798	-1.384923
C	1.881686	-3.130151	-0.127608
O	-1.571071	-2.832126	1.031597
Y	-1.873896	-2.149802	3.072843
C	-1.944189	-1.797540	-1.715845
C	-2.939278	-2.977956	-1.626866
C	3.142824	-2.337381	-2.192987
C	2.901570	-1.630972	-3.537865
O	-1.839480	-3.114977	5.012622
C	-2.730090	-2.515596	7.758793
C	-3.395035	-1.363323	6.977945
C	2.203249	-2.662473	9.042148
C	2.206750	-1.188335	9.507143
O	-0.180079	-0.436016	2.646949
C	-0.495481	0.735961	1.860172
O	-4.163250	-2.301449	2.987257
C	-5.054509	-3.306842	2.499186
C	-8.576621	-4.343650	5.209857
C	-9.931360	-4.069258	5.878508
C	1.520155	-2.004509	3.344359
C	1.210185	-0.805936	2.469458
C	-2.489016	-0.573986	-0.950962
C	-1.831078	-1.406463	-3.201330
C	4.139531	-1.485126	-1.374024
C	3.771357	-3.719891	-2.478647
C	-3.476494	-3.842270	7.482238
C	-2.870162	-2.204439	9.260155
C	1.891687	-3.575601	10.249590
C	3.612890	-3.006600	8.531496
C	-6.352721	-3.406626	3.314488
C	-6.169187	-3.479455	4.830556
C	-8.152272	-5.801189	5.433057
H	2.555869	-2.337809	3.133071
H	1.482424	-1.692823	4.398139
H	1.843332	0.042574	2.780768
H	1.385538	-1.008215	1.402820
H	0.128185	1.580359	2.191717
H	-1.554773	0.952027	2.024168
H	-0.308505	0.524931	0.795841
H	0.044190	-4.751252	2.014866
H	1.796358	-4.439330	2.085554
H	0.531762	-1.649714	-2.832151
H	2.823068	-3.512914	0.275645
H	1.705536	-4.482334	4.365925
H	-0.025998	-4.862051	4.180987
H	-0.461305	-2.267159	9.266913

H	2.425468	-3.597109	6.386819
H	-3.496565	-0.307606	-1.306860
H	-1.834733	0.296968	-1.103705
H	-2.543581	-0.775263	0.124352
H	-1.451451	-2.236512	-3.815490
H	-1.178161	-0.534618	-3.354300
H	-2.829103	-1.138904	-3.578770
H	-2.523553	-3.863788	-2.129486
H	-3.883506	-2.716286	-2.126862
H	-3.149861	-3.241240	-0.583662
H	3.713889	-0.494287	-1.156735
H	5.077831	-1.343973	-1.932496
H	4.387041	-1.963544	-0.415784
H	4.010327	-4.254662	-1.548558
H	4.704733	-3.609376	-3.052384
H	3.080304	-4.346901	-3.060779
H	2.224788	-2.207356	-4.185393
H	3.856529	-1.517407	-4.071444
H	2.474823	-0.626314	-3.400033
H	-4.512887	-3.778625	7.839936
H	-2.987333	-4.673835	8.011107
H	-3.491358	-4.066438	6.409096
H	-2.386930	-1.253425	9.530768
H	-2.446438	-3.001351	9.889460
H	-3.937307	-2.116611	9.511530
H	-2.840548	-0.423443	7.117785
H	-4.418966	-1.200986	7.349039
H	-3.457207	-1.569397	5.905604
H	0.911628	-3.344242	10.690025
H	2.652017	-3.448282	11.036154
H	1.883094	-4.632163	9.944316
H	3.687079	-4.058512	8.218223
H	4.347586	-2.845443	9.334082
H	3.902503	-2.371746	7.680984
H	2.431320	-0.516553	8.665501
H	2.968007	-1.031287	10.287151
H	1.232785	-0.893782	9.922295
H	-5.329436	-3.067119	1.455587
H	-6.984069	-2.534543	3.080391
H	-6.906889	-4.281206	2.937751
H	-5.475011	-2.680299	5.129204
C	-7.487550	-3.314344	5.616214
H	-5.676781	-4.423262	5.115126
H	-8.727735	-4.207051	4.125799
H	-8.004584	-6.017730	6.501613
H	-8.930198	-6.486795	5.066412
H	-7.218359	-6.045219	4.907200
H	-10.714418	-4.701892	5.434729
H	-9.906609	-4.293361	6.955108
H	-10.231648	-3.018218	5.759809
C	-7.175418	-3.306265	7.126894
H	-7.891963	-2.317950	5.367538
C	-4.261560	-4.616925	2.494608
C	-8.228919	-2.742427	8.053323
H	-6.920794	-4.308462	7.493807
H	-6.280928	-2.681071	7.283910
O	-8.711541	-1.565330	7.586252
O	-8.574972	-3.250012	9.108204
C	-9.722425	-0.878269	8.397625
C	-10.533949	-0.040488	7.423390
H	-10.351319	-1.652138	8.863086
C	-9.023088	-0.058383	9.472728
H	-11.340766	0.477712	7.959679
H	-9.900308	0.716726	6.939489
H	-10.982630	-0.671568	6.644867
H	-9.764663	0.484142	10.076302
H	-8.443834	-0.710555	10.138349
H	-8.345699	0.674893	9.010966
H	-3.885003	-4.836671	3.505183
H	-4.889734	-5.457493	2.167526
H	-3.408577	-4.544413	1.802121
C	-4.331321	-0.555524	3.017541
C	-4.939106	-0.170674	1.661187
C	-6.452546	-0.211700	1.390522
C	-7.158141	0.998015	2.030239
C	-6.380893	0.089317	4.307462
C	-6.688101	1.323583	3.455073
H	-4.571045	0.851726	1.471954

H	-6.871541	-1.129891	1.831269
H	-7.005919	1.883949	1.390602
H	-4.406714	-0.808504	0.949117
H	-8.242018	0.810678	2.047565
H	-5.776951	1.942110	3.444821
H	-7.458513	1.925328	3.960559
O	-3.052519	-0.273682	3.121479
O	-4.980795	-0.290444	4.194193
C	-6.729967	-0.296853	-0.136924
H	-6.126051	0.494913	-0.619362
C	-6.305413	-1.651213	-0.717892
H	-5.261679	-1.900745	-0.494244
H	-6.937900	-2.457312	-0.312212
H	-6.414901	-1.660853	-1.812229
C	-8.205542	-0.049279	-0.488754
H	-8.370074	-0.200278	-1.565694
H	-8.855872	-0.758509	0.048376
H	-8.536782	0.967570	-0.243020
H	-7.009480	-0.760950	3.995724
C	-6.586615	0.335050	5.792564
H	-5.977658	1.186474	6.128557
H	-7.644302	0.543506	5.997296
H	-6.302811	-0.548677	6.373947

III$_{CM,2}$ (in implicit toluene)

C	5.375652	-0.738029	2.056131
C	4.467292	-0.592588	1.000122
C	3.628428	-1.663326	0.606245
C	3.748313	-2.916321	1.283295
C	4.677171	-3.003095	2.324904
C	5.502155	-1.943917	2.746567
C	4.405518	0.692053	0.228063
N	3.100785	1.440063	0.297370
C	3.291064	2.671304	-0.536925
C	2.171023	3.669961	-0.561019
C	0.915080	3.282851	-1.098682
C	-0.110594	4.261990	-1.217926
C	0.162386	5.558610	-0.753108
C	1.382161	5.948569	-0.183791
C	2.381695	4.970104	-0.105915
O	0.737340	2.005845	-1.439334
Y	1.229092	0.009938	-0.776942
C	-1.493160	3.892134	-1.777131
C	-1.364188	3.212525	-3.160348
C	1.651009	7.367785	0.339365
C	0.434105	8.294459	0.175191
O	2.750792	-1.460633	-0.379809
C	2.875822	-4.118418	0.887187
C	1.390060	-3.787389	1.158002
C	6.480882	-2.153005	3.911688
C	5.693369	-2.547712	5.181731
O	0.600887	0.654189	1.439962
C	-0.761743	0.530433	1.902023
O	-1.933711	-1.607065	-2.488141
C	-2.754206	-0.500595	-2.020228
C	-4.406570	-0.803362	2.234807
C	-5.027079	-1.868923	3.150876
C	2.742308	1.741217	1.702726
C	1.244328	1.862101	1.932663
C	-2.177223	2.926834	-0.786019
C	-2.411304	5.116093	-1.947609
C	2.005039	7.300006	1.842583
C	2.836500	7.988089	-0.434935
C	3.096927	-4.451843	-0.606593
C	3.217237	-5.385351	1.693420
C	7.473803	-3.283268	3.556319
C	7.291365	-0.883514	4.225526
C	-3.636624	-1.011634	-0.877286
C	-4.454675	0.072627	-0.172123
C	-4.001714	0.435360	3.047300
H	3.230349	2.668488	2.056372
H	3.115990	0.908301	2.314708
H	1.051418	1.947237	3.014500
H	0.803332	2.730703	1.420749
H	-0.771465	0.397282	2.993604
H	-1.190036	-0.344615	1.402196
H	-1.335753	1.429452	1.632241

H	3.478863	2.308340	-1.563338	H	7.975131	-1.078644	5.064259
H	4.214965	3.181392	-0.203241	H	6.638950	-0.046028	4.514815
H	-0.626630	6.301726	-0.834924	H	4.980956	-1.756241	5.456709
H	3.360665	5.216229	0.313940	H	6.379842	-2.705228	6.028073
H	5.219492	1.362019	0.564545	H	5.125029	-3.476339	5.032682
H	4.571756	0.489642	-0.843275	H	-2.088813	0.292542	-1.648775
H	4.767869	-3.953686	2.848920	H	-4.312040	-1.784740	-1.282317
H	5.989677	0.123780	2.322612	H	-2.969189	-1.511813	-0.162200
H	-3.159060	2.613584	-1.169552	H	-5.126013	0.558009	-0.898785
H	-2.329023	3.414198	0.189079	C	-5.296476	-0.462556	1.005259
H	-1.564552	2.030107	-0.635331	H	-3.781404	0.865854	0.192108
H	-1.977839	5.858729	-2.634156	H	-3.484312	-1.245650	1.821988
H	-2.625786	5.612722	-0.989687	H	-4.859428	0.847765	3.598098
H	-3.372970	4.790264	-2.370592	H	-3.228432	0.174984	3.783846
H	-0.892845	3.896298	-3.881799	H	-3.598365	1.234114	2.407440
H	-2.361370	2.945325	-3.541596	H	-4.301022	-2.191190	3.912006
H	-0.757650	2.302732	-3.098683	H	-5.907367	-1.486451	3.686504
H	1.175067	6.861964	2.416270	H	-5.336961	-2.755701	2.578778
H	2.204626	8.307976	2.238425	C	-6.434245	0.542663	1.306812
H	2.899279	6.686050	2.019169	H	-5.775533	-1.399842	0.675036
H	3.752424	7.392169	-0.317211	C	-3.533713	0.003585	-3.223779
H	3.046810	9.005307	-0.069213	C	-7.384141	0.104880	2.396385
H	2.608709	8.047764	-1.509297	H	-6.027151	1.520865	1.592271
H	0.149014	8.410008	-0.880884	H	-7.023580	0.677503	0.386632
H	0.675581	9.293871	0.566149	O	-8.056577	-1.011331	2.022960
H	-0.439890	7.921764	0.729779	O	-7.526185	0.658457	3.475292
H	2.445235	-5.284648	-0.911844	C	-8.952543	-1.622171	3.014594
H	4.140816	-4.754736	-0.777992	C	-9.000227	-3.100425	2.669158
H	2.880253	-3.587421	-1.243820	H	-8.495454	-1.468510	4.004004
H	3.038327	-5.251591	2.770636	C	-10.307998	-0.931932	2.957286
H	4.263309	-5.695103	1.550795	H	-9.633537	-3.632451	3.392525
H	2.575625	-6.211362	1.352661	H	-9.419867	-3.250800	1.664125
H	1.235305	-3.589596	2.229433	H	-7.993650	-3.538431	2.698714
H	0.753283	-4.640171	0.874224	H	-10.993080	-1.388924	3.685464
H	1.048079	-2.913432	0.593871	H	-10.207434	0.134058	3.196911
H	6.953708	-4.232341	3.365655	H	-10.747226	-1.035104	1.954369
H	8.183053	-3.447453	4.382165	H	-4.189489	-0.786618	-3.617152
H	8.047154	-3.025747	2.653772	H	-4.154856	0.866153	-2.947123
H	7.900393	-0.566737	3.365940	H	-2.850855	0.321602	-4.022247

C	-0.695751	-1.771813	-1.879745
C	-0.220649	-3.207920	-2.056750
C	-0.274582	-3.843415	-3.451903
C	0.507058	-3.022082	-4.502064
C	0.160482	-0.496438	-4.073158
C	-0.182818	-1.718514	-4.941022
H	0.810080	-3.235453	-1.676211
H	-1.333118	-3.880926	-3.769121
H	1.513995	-2.806644	-4.106001
H	-0.834796	-3.780405	-1.347918
H	0.656799	-3.642630	-5.395799
H	0.094552	-1.475469	-5.978530
H	-1.270707	-1.871973	-4.930931
O	-0.558395	-1.292105	-0.647914
O	0.389573	-0.871239	-2.703638
C	0.247249	-5.310221	-3.408218
H	1.348435	-5.257914	-3.320047
C	-0.277785	-6.113498	-2.208038
H	0.086660	-5.726008	-1.248045
H	-1.379639	-6.102991	-2.181314
H	0.042475	-7.163161	-2.282115
C	-0.101365	-6.072100	-4.697217
H	0.324708	-7.085755	-4.678147
H	-1.194330	-6.170328	-4.797324
H	0.273099	-5.576116	-5.602655
H	-0.684161	0.213721	-4.098707
C	1.423800	0.230497	-4.522785
H	1.621665	1.088962	-3.862948
H	2.288593	-0.449197	-4.497816
H	1.304816	0.610081	-5.547274

TS3$_{CM,2}$ (in implicit toluene)

C	2.603577	-3.270609	1.706707
C	1.387625	-2.594391	1.549438
C	0.241533	-3.261958	1.054163
C	0.355089	-4.634257	0.671765
C	1.595500	-5.254534	0.847135
C	2.737814	-4.617015	1.365569
C	1.292561	-1.128673	1.852892
N	0.352876	-0.745943	2.965348
C	0.449942	0.745628	3.088744
C	-0.306635	1.395785	4.210729
C	-1.723307	1.299186	4.231594
C	-2.442766	2.004569	5.236951
C	-1.705191	2.725781	6.189633
C	-0.305573	2.798757	6.208593
C	0.373599	2.119773	5.188175
O	-2.321064	0.523622	3.327940
Y	-2.072879	-1.356813	2.293427
C	-3.976021	1.925983	5.309250
C	-4.609908	2.313756	3.953095
C	0.481510	3.571370	7.277751
C	-0.437574	4.246921	8.309886
O	-0.905773	-2.586084	0.965574
C	-0.849415	-5.396897	0.096427
C	-1.961038	-5.490595	1.167334
C	4.047534	-5.403135	1.524520
C	3.817614	-6.603615	2.470519
O	-1.521973	-2.358253	4.411340
C	-2.537631	-2.895589	5.284934
O	-5.917530	-1.888130	1.722338
C	-6.191223	-1.422596	3.078577
C	-5.880761	-4.533159	6.404268
C	-6.274979	-5.999864	6.635583
C	0.709524	-1.453328	4.216773
C	-0.471133	-1.672522	5.148133
C	-4.375199	0.480167	5.673780
C	-4.568198	2.859149	6.381135
C	1.413990	2.594847	8.030684
C	1.334235	4.668840	6.601585
C	-1.367178	-4.669399	-1.166845
C	-0.491850	-6.835622	-0.320355
C	4.508143	-5.924651	0.144149
C	5.176507	-4.539703	2.112540
C	-6.614337	-2.629689	3.919523
C	-6.754822	-2.338369	5.416382
C	-4.892575	-4.065734	7.483303

H	1.503292	-0.918954	4.771000
H	1.103945	-2.437644	3.928480
H	-0.153920	-2.313779	5.987000
H	-0.877908	-0.732387	5.549764
H	-2.104363	-3.687765	5.913123
H	-3.327865	-3.301332	4.645460
H	-2.949108	-2.099141	5.922852
H	0.089908	1.146865	2.124553
H	1.518621	1.021078	3.173771
H	-2.257051	3.253465	6.963401
H	1.465189	2.151775	5.138666
H	2.300904	-0.737161	2.086606
H	0.930619	-0.586943	0.962325
H	1.685778	-6.303341	0.566234
H	3.451416	-2.708364	2.102235
H	-5.470215	0.379114	5.702807
H	-3.978880	0.204810	6.662701
H	-3.983246	-0.226569	4.932183
H	-4.310870	3.912021	6.192440
H	-4.230449	2.596818	7.394554
H	-5.664961	2.774586	6.367693
H	-4.347663	3.348628	3.688679
H	-5.706675	2.243882	4.016752
H	-4.262409	1.652447	3.151906
H	0.829519	1.803483	8.522375
H	1.990323	3.129310	8.801622
H	2.129163	2.112525	7.349531
H	2.046613	4.241964	5.881358
H	1.911678	5.227911	7.354341
H	0.692981	5.379710	6.060484
H	-1.109070	4.980351	7.839148
H	0.172056	4.782312	9.052320
H	-1.053424	3.513358	8.850738
H	-2.255073	-5.183073	-1.566139
H	-0.593265	-4.669210	-1.949555
H	-1.633244	-3.629573	-0.947633
H	-0.158467	-7.443870	0.533951
H	0.293893	-6.858344	-1.090503
H	-1.384894	-7.320305	-0.741817

H	-1.597790	-6.054861	2.039684
H	-2.837410	-6.018875	0.760306
H	-2.292631	-4.504899	1.508617
H	3.756407	-6.586308	-0.308703
H	5.445296	-6.494794	0.239787
H	4.681656	-5.088100	-0.548378
H	5.411949	-3.682169	1.465049
H	6.091002	-5.142576	2.212307
H	4.917832	-4.157668	3.111294
H	3.491181	-6.258268	3.462557
H	4.747093	-7.181094	2.593801
H	3.047668	-7.284728	2.081359
H	-5.265090	-0.991160	3.484144
H	-7.569187	-3.013080	3.521841
H	-5.861275	-3.413688	3.757473
H	-7.531514	-1.572320	5.570446
C	-7.107228	-3.588239	6.249032
H	-5.818349	-1.899970	5.798017
H	-5.354042	-4.501536	5.435408
H	-5.311174	-4.200668	8.491136
H	-3.962749	-4.649706	7.429540
H	-4.628257	-3.003962	7.369571
H	-5.387954	-6.648331	6.582773
H	-6.726571	-6.151164	7.626142
H	-6.994321	-6.343290	5.878155
C	-7.733456	-3.139943	7.591734
H	-7.886505	-4.145582	5.702225
C	-7.254618	-0.344399	2.957236
C	-8.202131	-4.274717	8.472074
H	-7.026425	-2.535668	8.173804
H	-8.608856	-2.511465	7.366511
O	-9.203177	-4.961564	7.869171
O	-7.742515	-4.556221	9.567422
C	-9.707662	-6.161113	8.552979
C	-10.229764	-7.070188	7.453839
H	-8.853254	-6.625103	9.068510
C	-10.765173	-5.751698	9.568471
H	-10.603296	-8.006969	7.890260
H	-11.054778	-6.587506	6.910504

H	-9.432032	-7.311904	6.739116
H	-11.159391	-6.642090	10.078258
H	-10.335113	-5.081494	10.323659
H	-11.599085	-5.239930	9.066760
H	-8.175709	-0.756874	2.521122
H	-7.491800	0.070777	3.946069
H	-6.900754	0.477564	2.321805
C	-4.643428	-2.310687	1.455729
C	-4.593982	-3.177884	0.212710
C	-5.374162	-2.750663	-1.037028
C	-4.932567	-1.367459	-1.568205
C	-4.446369	0.263553	0.377677
C	-5.418620	-0.165416	-0.738503
H	-3.530802	-3.303530	-0.032136
H	-6.444163	-2.687835	-0.765861
H	-3.832566	-1.355880	-1.645587
H	-4.951916	-4.154739	0.569127
H	-5.312589	-1.248784	-2.591830
H	-5.583797	0.703975	-1.393944
H	-6.392075	-0.412660	-0.292204
O	-3.882097	-2.672695	2.454839
O	-3.750134	-0.859390	0.911575
C	-5.244525	-3.827014	-2.157396
H	-4.234080	-3.717702	-2.593989
C	-5.376575	-5.270991	-1.648177
H	-4.553607	-5.562242	-0.982339
H	-6.322861	-5.408971	-1.100399
H	-5.376593	-5.974418	-2.494027
C	-6.281399	-3.605321	-3.270544
H	-6.135929	-4.326308	-4.088384
H	-7.299652	-3.748955	-2.874538
H	-6.232502	-2.599218	-3.707849
H	-5.026716	0.739683	1.189950
C	-3.386807	1.256775	-0.098564
H	-2.721363	1.522654	0.736780
H	-2.785849	0.815219	-0.907756
H	-3.854461	2.179765	-0.470532

IV$_{CM,2}$ (in implicit toluene)

C	5.362349	-0.746210	1.814304
C	4.388039	-0.609140	0.817962
C	3.488841	-1.668291	0.528075
C	3.582523	-2.873081	1.295151
C	4.579143	-2.953098	2.270948
C	5.491608	-1.920447	2.557632
C	4.282681	0.674938	0.043861
N	2.965525	1.398109	0.189014
C	3.103097	2.713955	-0.516566
C	1.967957	3.694587	-0.381976
C	0.661064	3.329858	-0.811753
C	-0.355847	4.327729	-0.828884
C	-0.032504	5.611458	-0.364605
C	1.235432	5.974961	0.110087
C	2.225452	4.983666	0.082381
O	0.431090	2.075309	-1.169968
Y	1.278357	0.075419	-1.138460
C	-1.765909	3.989006	-1.338211
C	-1.696248	3.457151	-2.789566
C	1.567480	7.382230	0.629295
C	0.353671	8.326146	0.583625
O	2.588508	-1.505470	-0.430002
C	2.599761	-4.029229	1.051189
C	1.155351	-3.538676	1.313266
C	6.551945	-2.116572	3.650920
C	5.856611	-2.391754	5.003759
O	0.600458	0.217560	1.300279
C	-0.746728	-0.071742	1.696964
O	-2.299357	-2.114911	-2.492274
C	-3.110048	-0.902288	-2.212342
C	-4.508926	-0.384357	2.065834
C	-4.986685	-1.243851	3.246368
C	2.582233	1.548284	1.615129
C	1.082495	1.464321	1.857928
C	-2.393392	2.912338	-0.426740
C	-2.705326	5.208486	-1.330463
C	2.048623	7.289425	2.095414

C	2.689695	8.000688	-0.235831
C	2.741318	-4.530194	-0.404821
C	2.853392	-5.229607	1.980755
C	7.451358	-3.320394	3.288098
C	7.451282	-0.879803	3.817730
C	-3.915679	-1.226898	-0.956106
C	-4.802016	-0.080145	-0.467829
C	-4.135730	1.026142	2.542106
H	2.955863	2.501144	2.029650
H	3.058266	0.727971	2.167751
H	0.897585	1.467115	2.946382
H	0.531555	2.301120	1.402479
H	-0.814699	-0.157105	2.793497
H	-1.014021	-1.029305	1.237209
H	-1.438511	0.712338	1.348145
H	3.256266	2.471970	-1.584231
H	4.031337	3.204290	-0.165708
H	-0.814179	6.367025	-0.373053
H	3.240335	5.214579	0.417988
H	5.100625	1.352665	0.352867
H	4.406812	0.489714	-1.037572
H	4.655026	-3.872104	2.851692
H	6.023104	0.102549	1.999050
H	-3.425538	2.703792	-0.747927
H	-2.423866	3.259117	0.616912
H	-1.813583	1.981700	-0.469969
H	-2.327623	6.020176	-1.969867
H	-2.856058	5.607603	-0.316243
H	-3.690515	4.910267	-1.719162
H	-1.291152	4.228073	-3.461721
H	-2.705338	3.192093	-3.143517
H	-1.055175	2.569792	-2.852836
H	1.264018	6.855849	2.733060
H	2.297829	8.288944	2.484807
H	2.944302	6.659362	2.186641
H	3.604931	7.393071	-0.204556
H	2.943051	9.010633	0.122865
H	2.371123	8.076421	-1.285864
H	-0.017215	8.463810	-0.442828
H	0.640188	9.315639	0.969397
H	-0.475564	7.953664	1.203454
H	2.009991	-5.328121	-0.608609
H	3.747707	-4.943408	-0.569379
H	2.585987	-3.713629	-1.119182
H	2.741012	-4.960105	3.041750
H	3.856635	-5.657303	1.836361
H	2.120887	-6.020342	1.759506
H	1.044890	-3.221542	2.360918
H	0.434847	-4.350179	1.122438
H	0.904155	-2.686441	0.672775
H	6.868036	-4.247428	3.197449
H	8.216955	-3.478004	4.063553
H	7.960622	-3.147786	2.328519
H	7.998085	-0.646268	2.892002
H	8.194829	-1.066315	4.606148
H	6.870917	0.008264	4.109804
H	5.213196	-1.545571	5.286856
H	6.603271	-2.540843	5.799305
H	5.228717	-3.292440	4.959129
H	-2.416649	-0.070671	-2.021533
H	-4.532215	-2.117602	-1.159729
H	-3.198865	-1.506512	-0.169132
H	-5.565327	0.136124	-1.231137
C	-5.506437	-0.386078	0.871465
H	-4.200917	0.836208	-0.366887
H	-3.586876	-0.859446	1.690747
H	-4.984533	1.514879	3.041681
H	-3.308349	0.978934	3.264717
H	-3.812956	1.671136	1.712506
H	-4.188618	-1.340885	3.997534
H	-5.852955	-0.796307	3.753064
H	-5.269686	-2.254265	2.917746
C	-6.693006	0.592071	1.040387
H	-5.933631	-1.400780	0.799602
C	-3.924319	-0.646097	-3.464212
C	-7.544194	0.336939	2.262871
H	-6.342747	1.630857	1.087183
H	-7.345287	0.495248	0.158875

O	-8.130493	-0.882950	2.186869
O	-7.682298	1.106149	3.200058
C	-8.922737	-1.324468	3.344115
C	-8.855640	-2.841805	3.331287
H	-8.434900	-0.921191	4.244295
C	-10.334367	-0.768895	3.226765
H	-9.406474	-3.246923	4.191470
H	-9.305071	-3.242821	2.411441
H	-7.814057	-3.184312	3.392464
H	-10.941013	-1.104368	4.079939
H	-10.318248	0.328296	3.223172
H	-10.809314	-1.124424	2.300916
H	-4.615117	-1.477599	-3.661427
H	-4.509477	0.274970	-3.342017
H	-3.264787	-0.515236	-4.331963
C	-1.110408	-2.222207	-1.912809
C	-0.477567	-3.568566	-2.091673
C	0.058188	-3.874343	-3.515050
C	1.126053	-2.835009	-3.921399
C	1.531952	-0.367091	-4.504977
C	0.585150	-1.581429	-4.621002
H	0.344202	-3.637638	-1.369794
H	-0.797544	-3.817444	-4.212084
H	1.682108	-2.531200	-3.017907
H	-1.248669	-4.302648	-1.821062
H	1.870637	-3.310435	-4.575160
H	0.374556	-1.804114	-5.680900
H	-0.375297	-1.273387	-4.175311
O	-0.613685	-1.298763	-1.246724
O	1.651287	-0.000478	-3.161262
C	0.630626	-5.317296	-3.592572
H	1.628487	-5.290796	-3.119992
C	-0.208547	-6.370906	-2.856998
H	-0.211061	-6.223580	-1.767964
H	-1.253094	-6.361218	-3.208420
H	0.194112	-7.376549	-3.046165
C	0.797195	-5.736628	-5.061952
H	1.315195	-6.703498	-5.135591
H	-0.189098	-5.844386	-5.541063
H	1.373012	-5.004770	-5.644663
H	1.061919	0.464760	-5.072999
C	2.914326	-0.629283	-5.114178
H	3.520936	0.284984	-5.057446
H	3.436106	-1.415094	-4.549855
H	2.840113	-0.937458	-6.168857

Figure S60 and Figure S61

II$_{M,si}$ (in implicit toluene)

C	0.156009	-5.490407	3.504398
C	-0.743639	-4.425393	3.641777
C	-1.914056	-4.564624	4.434576
C	-2.186009	-5.829603	5.043824
C	-1.254943	-6.853865	4.864820
C	-0.071376	-6.725424	4.115873
C	-0.500986	-3.148211	2.878630
N	-0.542177	-1.868876	3.674135
C	0.021928	-0.786930	2.807070
C	-0.033674	0.616375	3.352568
C	-1.305129	1.229968	3.536603
C	-1.353987	2.604266	3.922352
C	-0.143044	3.243622	4.226593
C	1.115570	2.629739	4.123239
C	1.138650	1.312519	3.645417
O	-2.398722	0.511358	3.338637
Y	-3.019312	-1.443126	4.094883
O	-4.602986	-0.612149	5.611004
C	-5.814094	-0.531503	5.865983
O	-6.588745	-1.517918	5.453722
C	-8.047531	-1.566952	5.713507
C	-8.436973	-3.015332	5.383078
C	-9.932214	-3.257173	5.635079
C	-2.697149	3.358649	3.951197
C	-3.366617	3.252367	2.559280
C	2.426569	3.335146	4.501987
C	2.198975	4.775854	4.990925
O	-2.729724	-3.532132	4.568867
C	-3.505037	-6.065191	5.795998
C	-3.658110	-5.064853	6.964290
C	0.898070	-7.912986	4.028277
C	1.406736	-8.256345	5.447730
O	-1.558004	-0.781085	6.089037
C	-1.866184	0.350121	6.920415
O	-4.290505	-1.755543	2.526616
C	-5.236652	-2.054022	1.551171
C	-6.557482	-1.313076	1.872043
C	-7.669377	-1.588972	0.854483
C	0.182799	-2.026818	4.953176
C	-0.128014	-0.951723	5.980318
C	-3.649932	2.763720	5.011963
C	-2.520910	4.853729	4.274085
C	3.116274	2.546461	5.639286
C	3.366476	3.386444	3.276668
C	-4.671185	-5.901254	4.794096
C	-3.597598	-7.486992	6.378939
C	0.168214	-9.142371	3.442690
C	2.120618	-7.605028	3.147833
C	-5.419186	-3.581150	1.432610
C	-4.077586	-4.306609	1.321760
C	-6.302565	0.193474	2.003616
C	-6.376591	0.664302	6.578952
C	-7.285750	1.531442	5.668661
C	-7.397974	2.939869	6.263762
C	-8.684197	0.921459	5.490154
C	-8.749415	-0.488839	4.894074
C	-7.595492	-4.001935	6.204166
H	1.279303	-2.046842	4.793367
H	-0.104056	-3.003888	5.361615
H	0.273349	-1.267474	6.960191
H	0.320777	0.016124	5.712576
H	-1.449411	0.205808	7.930910
H	-2.956073	0.423630	6.970977
H	-1.454977	1.269569	6.471263
H	-0.558703	-0.830718	1.870514
H	1.072052	-1.040524	2.560177
H	-0.182609	4.279891	4.553695
H	2.091929	0.793098	3.512101
H	0.480877	-3.223916	2.374225
H	-1.258679	-3.033886	2.081248
H	-1.462414	-7.820082	5.321193
H	1.047505	-5.329659	2.895398

H	-4.622076	3.279469	4.966636
H	-3.242941	2.897336	6.025258
H	-3.817177	1.695464	4.839776
H	-1.880946	5.359509	3.536098
H	-2.088059	5.012071	5.273366
H	-3.504048	5.346874	4.257143
H	-2.716681	3.689714	1.786822
H	-4.320323	3.803290	2.556840
H	-3.565342	2.205624	2.299920
H	2.466836	2.502006	6.525989
H	4.064079	3.027160	5.928438
H	3.340840	1.514660	5.333891
H	3.606524	2.379198	2.908607
H	4.314016	3.884184	3.536377
H	2.898081	3.943834	2.452379
H	1.736850	5.401921	4.213496
H	3.163675	5.232149	5.257329
H	1.557820	4.806211	5.884089
H	-5.633444	-6.068024	5.301972
H	-4.574144	-6.646606	3.991666
H	-4.676943	-4.897979	4.350208
H	-2.795996	-7.686900	7.105711
H	-3.556658	-8.252788	5.590588
H	-4.558533	-7.599525	6.902831
H	-2.840186	-5.193255	7.689586
H	-4.610258	-5.243190	7.488523
H	-3.642436	-4.030925	6.602196
H	-0.688403	-9.440370	4.062197
H	0.853904	-10.001597	3.378355
H	-0.217335	-8.932049	2.436698
H	1.831735	-7.363942	2.115221
H	2.782188	-8.483278	3.111646
H	2.706572	-6.762785	3.546085
H	1.932149	-7.397136	5.890548
H	2.103714	-9.108395	5.412204
H	0.578040	-8.526170	6.117084
H	-4.878549	-1.679172	0.562988
H	-5.948759	-3.941349	2.333643
H	-6.056947	-3.809340	0.564178
H	-3.505455	-4.085941	2.233547
C	-4.171327	-5.829836	1.152738
H	-3.496506	-3.885102	0.479990
H	-6.867883	-1.702122	2.858608
H	-5.966053	0.612855	1.041964
H	-7.217614	0.732333	2.296090
H	-5.518375	0.401590	2.743480
H	-8.565571	-0.991091	1.082186
H	-7.343068	-1.317615	-0.162766
H	-7.969237	-2.645527	0.842737
H	-6.943816	0.342904	7.468184
H	-6.797728	1.605429	4.682115
H	-9.182670	0.913165	6.475979
H	-5.515304	1.251839	6.918065
H	-9.276216	1.597363	4.853458
H	-9.807618	-0.768010	4.800624
H	-8.331527	-0.507848	3.877357
H	-8.222812	-3.170710	4.311130
H	-6.521958	-3.879698	6.020891
H	-7.781346	-3.863749	7.281749
H	-7.863602	-5.035829	5.948217
H	-10.167533	-4.318348	5.476671
H	-10.199258	-3.008957	6.674644
H	-10.578546	-2.673286	4.967121
H	-8.195884	-1.398713	6.795297
H	-7.863503	2.904911	7.261184
H	-6.411214	3.412228	6.365954
H	-8.018482	3.582369	5.623171
C	-2.788067	-6.444092	1.461684
H	-4.874394	-6.220309	1.906345
C	-4.673345	-6.241399	-0.236170
C	-2.820192	-7.953945	1.503246
H	-2.044403	-6.040017	0.763842
H	-2.480824	-6.136940	2.470906
O	-2.155761	-8.694191	0.567027

O	-3.440371	-8.560592	2.358849
C	-1.439200	-8.088978	-0.558884
C	-1.450362	-9.140593	-1.657762
H	-2.001375	-7.206474	-0.898705
C	-0.033451	-7.692203	-0.127559
H	-0.936364	-8.757129	-2.550245
H	-0.934030	-10.050549	-1.320834
H	-2.480268	-9.404113	-1.931924
H	0.496955	-7.210890	-0.961635
H	-0.058901	-6.992189	0.719217
H	0.534783	-8.580972	0.180151
H	-4.729444	-7.335755	-0.338692
H	-5.677319	-5.840420	-0.434926
H	-4.001652	-5.857921	-1.021227

$II_{M,axial}$ (in implicit toluene)

C	0.039365	-5.359011	3.127442
C	-0.718594	-4.202374	3.353111
C	-1.938481	-4.275633	4.074967
C	-2.357338	-5.538147	4.600745
C	-1.560168	-6.653466	4.337700
C	-0.367589	-6.609781	3.592755
C	-0.255562	-2.904949	2.733531
N	-0.301800	-1.674059	3.581832
C	0.396733	-0.598390	2.813041
C	0.278076	0.812646	3.324532
C	-0.993861	1.449064	3.288297
C	-1.077010	2.833259	3.628820
C	0.088342	3.465573	4.088704
C	1.336979	2.833756	4.194775
C	1.405551	1.500893	3.771287
O	-2.060200	0.731116	2.950368
Y	-2.831449	-1.054280	3.944056
O	-3.871102	-1.589677	1.892264
C	-3.937178	-2.321694	0.895628
O	-4.702562	-1.884125	-0.099882
C	-4.721577	-2.528695	-1.433644
C	-5.304088	-1.459727	-2.372011
C	-5.286191	-1.944256	-3.829453
C	-2.405817	3.601266	3.496641
C	-2.959910	3.440149	2.061294
C	2.590076	3.532628	4.743339
C	2.320662	4.993059	5.144171
O	-2.666853	-3.180505	4.240484
C	-3.670237	-5.650818	5.391554
C	-3.615718	-4.726663	6.630005
C	0.402007	-7.907410	3.309706
C	0.804282	-8.581300	4.640679
O	-1.391244	-0.478762	5.843922
C	-1.658111	0.737163	6.568756
O	-4.505679	-0.397851	4.978648
C	-5.769251	-0.427552	5.584634
C	-5.680548	-0.229566	7.121644
C	-5.352356	1.231449	7.453232
C	0.292763	-1.934397	4.906506
C	0.007235	-0.833602	5.913118
C	-3.441814	3.070365	4.513643
C	-2.232994	5.109911	3.757232
C	3.084066	2.772977	5.996420
C	3.704362	3.524741	3.672617
C	-4.852296	-5.238981	4.484130
C	-3.938912	-7.083125	5.885945
C	-0.507445	-8.866001	2.506350
C	1.680055	-7.661493	2.490550
C	-6.519886	-1.717768	5.202839
C	-6.912298	-1.724906	3.724340
C	-4.691185	-1.205605	7.766731
C	-3.247582	-3.651992	0.812020
C	-4.203825	-4.860521	0.648306
C	-3.456555	-6.130415	1.073145
C	-4.735708	-5.019794	-0.781874
C	-5.506208	-3.832937	-1.364667
C	-4.537218	-0.134304	-2.250689
H	1.391259	-2.074444	4.842128

H	-0.136628	-2.877459	5.268819
H	0.236940	-1.192314	6.931219
H	0.603007	0.068540	5.712803
H	-1.409514	0.606566	7.633340
H	-2.727684	0.932975	6.456812
H	-1.079307	1.567555	6.133937
H	-0.045894	-0.635503	1.803855
H	1.468619	-0.864990	2.715750
H	0.017246	4.510749	4.379723
H	2.358223	0.963740	3.797454
H	0.779309	-3.052981	2.366009
H	-0.862583	-2.666699	1.841576
H	-1.887405	-7.620362	4.718159
H	0.965369	-5.256283	2.559247
H	-4.390622	3.619852	4.407460
H	-3.081722	3.223242	5.541765
H	-3.653037	2.003401	4.373829
H	-1.505597	5.564380	3.067910
H	-1.910160	5.319015	4.787763
H	-3.198469	5.614688	3.606544
H	-2.243853	3.837779	1.326254
H	-3.902311	4.000568	1.958551
H	-3.149129	2.385770	1.829978
H	2.306316	2.764409	6.774882
H	3.984338	3.251619	6.412359
H	3.335084	1.729055	5.760383
H	3.974801	2.501077	3.377836
H	4.612649	4.015464	4.055755
H	3.376527	4.060163	2.769495
H	1.990963	5.596823	4.285666
H	3.243481	5.445451	5.536011
H	1.554300	5.065587	5.930141
H	-5.792750	-5.249865	5.056627
H	-4.960726	-5.936444	3.641167
H	-4.699903	-4.229709	4.084145
H	-3.145823	-7.440810	6.559587
H	-4.035096	-7.795290	5.052775
H	-4.885285	-7.100015	6.446021
H	-2.795349	-5.027675	7.298663
H	-4.559040	-4.795803	7.193805
H	-3.461988	-3.682774	6.337073
H	-1.420014	-9.117702	3.064725
H	0.022272	-9.804941	2.281475
H	-0.813633	-8.402964	1.556946
H	1.455172	-7.216559	1.509686
H	2.195083	-8.616822	2.312146
H	2.380810	-6.997786	3.018398
H	1.456479	-7.919298	5.228974
H	1.346857	-9.520094	4.448690
H	-0.074214	-8.821533	5.255714
H	-6.371384	0.425796	5.196490
H	-5.867809	-2.578712	5.424714
H	-7.417965	-1.812893	5.834217
H	-6.018819	-1.497681	3.121413
C	-7.532178	-3.033029	3.213065
H	-7.623956	-0.898444	3.540674
H	-6.686937	-0.443381	7.525186
H	-3.689500	-1.090204	7.329515
H	-4.616476	-1.032403	8.850607
H	-4.995519	-2.249934	7.612407
H	-5.189930	1.376757	8.531904
H	-4.447905	1.559812	6.924187
H	-6.168557	1.898931	7.138011
H	-2.519712	-3.639258	-0.018238
H	-5.063790	-4.716042	1.322673
H	-3.881263	-5.247820	-1.445805
H	-2.689388	-3.767148	1.746060
H	-5.394240	-5.900793	-0.802215
H	-5.825183	-4.098719	-2.381954
H	-6.426342	-3.651747	-0.791608
H	-6.351543	-1.293879	-2.063462
H	-4.563767	0.264393	-1.229574
H	-3.482287	-0.272983	-2.536164
H	-4.969993	0.618082	-2.924706

H	-5.651560	-1.147770	-4.492291
H	-4.260774	-2.194232	-4.144522
H	-5.917781	-2.826716	-3.994309
H	-3.671502	-2.714113	-1.721446
H	-2.597102	-6.315563	0.409096
H	-3.071741	-6.055113	2.098611
H	-4.121698	-7.003870	1.018271
C	-7.825658	-2.893840	1.700364
H	-6.789699	-3.838694	3.320772
C	-8.793393	-3.433902	3.986497
C	-8.271330	-4.216555	1.111431
H	-8.542272	-2.079385	1.536766
H	-6.896808	-2.616319	1.180824
O	-9.554106	-4.406488	0.705386
O	-7.506354	-5.162700	1.015246
C	-10.576934	-3.353990	0.743040
C	-11.905049	-4.085676	0.854107
H	-10.424092	-2.744643	1.645734
C	-10.464495	-2.498720	-0.511598
H	-12.729052	-3.360177	0.901655
H	-12.060167	-4.734676	-0.019161
H	-11.931135	-4.704190	1.760645
H	-11.243831	-1.723479	-0.512969
H	-9.486648	-2.001220	-0.570905
H	-10.590804	-3.124660	-1.405951
H	-9.252072	-4.343201	3.570460
H	-8.563902	-3.636342	5.041049
H	-9.544244	-2.626853	3.957889

$\mathbf{II}_{\mathbf{M},\kappa\text{-}3}$ (in implicit toluene)

C	0.120485	-5.633525	4.207191
C	-0.667116	-4.516992	3.897351
C	-2.071300	-4.538322	4.129537
C	-2.643322	-5.732159	4.687286
C	-1.807781	-6.824174	4.930860
C	-0.420770	-6.818659	4.702371
C	0.023174	-3.363912	3.212674
N	-0.278440	-1.984592	3.690005
C	0.508752	-1.072224	2.802909
C	0.487388	0.394707	3.127855
C	-0.730650	1.122258	3.007691
C	-0.690945	2.538016	3.197833
C	0.519444	3.117691	3.609268
C	1.709917	2.401874	3.798455
C	1.665613	1.030949	3.516978
O	-1.851571	0.468440	2.730041
Y	-2.859345	-1.379182	3.294047
O	-4.429134	-1.195816	5.117905
C	-5.621389	-1.214748	5.432545
O	-5.887270	-1.007619	6.726087
C	-7.265577	-1.001386	7.247033
C	-7.160573	-0.399282	8.658143
C	-8.557405	-0.175510	9.256728
C	-1.939479	3.406205	2.960837
C	-2.512856	3.125247	1.551665
C	3.017773	3.051979	4.274126
C	2.857969	4.556096	4.554778
O	-2.821661	-3.485957	3.849557
C	-4.139457	-5.787801	5.040057
C	-4.438975	-4.711200	6.109294
C	0.411961	-8.075366	4.991190
C	0.274148	-8.464035	6.480589
O	-1.801486	-0.553974	5.618437
C	-2.317562	0.557346	6.359271
O	-4.613120	-0.964363	2.173292
C	-5.246856	-0.313975	1.107822
C	-6.361818	0.648773	1.610073
C	-6.942507	1.508732	0.478678
C	0.114223	-1.909017	5.112195
C	-0.390999	-0.688412	5.851263
C	-3.000653	3.090416	4.035231
C	-1.634332	4.914114	3.038739
C	3.483568	2.367639	5.580004

C	4.108085	2.877177	3.192990
C	-4.993072	-5.543865	3.776167
C	-4.560144	-7.149918	5.622034
C	-0.100006	-9.238747	4.111033
C	1.905192	-7.867743	4.685870
C	-5.837405	-1.316813	0.091336
C	-4.851375	-2.399967	-0.363585
C	-5.848427	1.545405	2.735227
C	-6.717590	-1.486401	4.449020
C	-7.404586	-2.853463	4.672709
C	-8.069254	-3.296005	3.367361
C	-8.409443	-2.830336	5.830942
C	-7.837977	-2.418689	7.193449
C	-6.380035	0.923327	8.644092
H	1.218145	-1.955433	5.221256
H	-0.300919	-2.807336	5.591997
H	-0.211943	-0.833976	6.933682
H	0.121543	0.233693	5.537229
H	-2.203908	0.380376	7.442974
H	-3.379181	0.650158	6.116765
H	-1.786540	1.481098	6.078508
H	0.100280	-1.233101	1.791859
H	1.564108	-1.414181	2.793028
H	0.533617	4.190471	3.785571
H	2.567843	0.420976	3.615453
H	1.118068	-3.529177	3.295878
H	-0.200475	-3.372383	2.140429
H	-2.251452	-7.732489	5.336924
H	1.192560	-5.555722	4.017888
H	-3.918212	3.672363	3.857665
H	-2.621246	3.352131	5.033893
H	-3.259301	2.026409	4.022845
H	-0.873812	5.216509	2.303487
H	-1.289799	5.218453	4.037954
H	-2.553699	5.478367	2.822127
H	-1.763223	3.361365	0.781589
H	-3.397155	3.756646	1.371088

H	-2.801995	2.073598	1.445944
H	2.723031	2.479647	6.366965
H	4.422749	2.816390	5.939572
H	3.658791	1.292791	5.431208
H	4.296972	1.815641	2.979819
H	5.056820	3.329875	3.521987
H	3.801666	3.360103	2.253419
H	2.562856	5.110209	3.651431
H	3.815600	4.970878	4.902694
H	2.107146	4.746323	5.335936
H	-6.066592	-5.622741	4.009799
H	-4.761796	-6.302703	3.013245
H	-4.795977	-4.552715	3.353994
H	-4.022744	-7.386111	6.552659
H	-4.395410	-7.971780	4.909045
H	-5.634578	-7.124392	5.857467
H	-3.835395	-4.893064	7.011049
H	-5.500206	-4.749369	6.403142
H	-4.205562	-3.707537	5.735830
H	-1.156494	-9.462957	4.314478
H	0.483432	-10.153919	4.298559
H	-0.009623	-8.983029	3.044801
H	2.071668	-7.620862	3.626791
H	2.461055	-8.791225	4.905126
H	2.337837	-7.063059	5.298504
H	0.636874	-7.651181	7.126749
H	0.860684	-9.369758	6.701056
H	-0.771795	-8.666788	6.750465
H	-4.500418	0.313214	0.564053
H	-6.730284	-1.784083	0.544455
H	-6.194256	-0.759611	-0.787957
H	-3.858193	-1.937708	-0.457748
C	-4.789215	-3.602140	0.599897
H	-5.125444	-2.770627	-1.367331
H	-7.171626	0.010580	2.010029
H	-5.079161	2.229325	2.353040
H	-6.659648	2.152613	3.164911

H	-5.387021	0.955204	3.536869
H	-7.659165	2.244486	0.874780
H	-6.139700	2.068257	-0.028173
H	-7.468085	0.911994	-0.278989
H	-7.466966	-0.677380	4.483437
H	-6.615665	-3.581763	4.912385
H	-9.233919	-2.143353	5.567417
H	-6.223988	-1.454617	3.466415
H	-8.860765	-3.831134	5.925121
H	-8.636765	-2.500880	7.943307
H	-7.040642	-3.111385	7.509125
H	-6.614541	-1.126785	9.284295
H	-5.354936	0.789326	8.278498
H	-6.880312	1.660278	7.995513
H	-6.332296	1.346751	9.657096
H	-8.470742	0.275557	10.254919
H	-9.142631	0.515292	8.629317
H	-9.130454	-1.105742	9.363915
H	-7.859378	-0.313390	6.618767
H	-8.837044	-2.572497	3.051075
H	-7.325321	-3.373845	2.563436
H	-8.550850	-4.278305	3.482188
C	-3.492877	-4.401552	0.448046
H	-4.829585	-3.202106	1.622329
C	-5.978891	-4.545459	0.380086
C	-2.222613	-3.618079	0.656077
H	-3.462103	-4.868724	-0.549592
H	-3.471963	-5.233613	1.173532
O	-1.060521	-4.120599	0.200118
O	-2.161755	-2.520001	1.207519
C	-0.901389	-5.518427	-0.247183
C	-0.690003	-6.409012	0.968223
H	-1.806680	-5.817994	-0.794412
C	0.285913	-5.499960	-1.194066
H	-0.618287	-7.457675	0.647379
H	0.237315	-6.137727	1.488942
H	-1.507163	-6.322527	1.696929
H	0.472987	-6.514154	-1.572696
H	0.097304	-4.836029	-2.047727
H	1.186645	-5.153325	-0.668531
H	-6.019147	-5.334839	1.144629
H	-6.930464	-3.997464	0.415361
H	-5.915016	-5.028949	-0.608525

$II_{CM,si}$ (in implicit toluene)

C	2.232637	-1.573928	4.471609
C	1.753310	-1.445788	3.162276
C	0.468407	-0.899906	2.907795
C	-0.320556	-0.455678	4.015439
C	0.207574	-0.617048	5.298829
C	1.470721	-1.175663	5.571860
C	2.609056	-1.861601	1.999151
N	2.936000	-0.752883	1.026760
C	3.964122	-1.278858	0.071224
C	4.518873	-0.274954	-0.900512
C	3.644122	0.316929	-1.854815
C	4.191753	1.212962	-2.818581
C	5.557545	1.523310	-2.730906
C	6.422959	0.988040	-1.765778
C	5.868054	0.071373	-0.862510
O	2.352380	0.031175	-1.800775
Y	0.808312	-0.398854	-0.323813
C	3.304677	1.792811	-3.933350
C	2.715311	0.624869	-4.758843
C	7.912958	1.352041	-1.677752
C	8.327239	2.389390	-2.735657
O	0.050201	-0.804405	1.658327
C	-1.689844	0.201010	3.779853
C	-1.507209	1.483814	2.933631
C	1.949725	-1.305168	7.025506
C	1.988124	0.092151	7.685752
O	1.603377	1.753412	0.769934
C	1.107618	3.046655	0.399035
O	0.100187	-2.135731	-1.179595

C	-0.438753	-3.416403	-1.296338
C	-5.012565	-3.695722	-1.002364
C	-6.369383	-3.503011	-1.698113
C	3.395574	0.468468	1.732593
C	3.025987	1.763035	1.024607
C	2.157233	2.636080	-3.330315
C	4.090196	2.699457	-4.898133
C	8.217546	1.942926	-0.282233
C	8.768124	0.081772	-1.888767
C	-2.624839	-0.778311	3.035349
C	-2.383539	0.601372	5.094002
C	0.975399	-2.211294	7.812413
C	3.357432	-1.917469	7.122336
C	-1.948704	-3.355696	-1.594258
C	-2.727769	-2.589645	-0.526737
C	-5.182641	-4.489337	0.299814
H	4.488906	0.450410	1.892281
H	2.911024	0.473228	2.717059
H	3.268529	2.609871	1.690590
H	3.563293	1.896762	0.073403
H	1.284753	3.768683	1.212668
H	0.030892	2.938281	0.231274
H	1.593393	3.403480	-0.523823
H	3.481310	-2.108851	-0.474814
H	4.799827	-1.713246	0.653447
H	5.967675	2.220502	-3.457455
H	6.496423	-0.394194	-0.097920
H	3.552640	-2.295033	2.380261
H	2.108668	-2.646603	1.403723
H	-0.391965	-0.284243	6.145747
H	3.231971	-1.990885	4.608932
H	1.533695	3.061898	-4.132891
H	2.565600	3.471849	-2.741782
H	1.519806	2.024083	-2.681994
H	4.905096	2.156714	-5.399545
H	4.519946	3.572666	-4.384921
H	3.410811	3.074723	-5.678110
H	3.523668	0.005306	-5.174809
H	2.124135	1.014447	-5.602875
H	2.075521	-0.010801	-4.133734
H	7.620500	2.850349	-0.108335
H	9.283078	2.208630	-0.197733
H	7.985395	1.227461	0.519145
H	8.545018	-0.684178	-1.132900
H	9.841124	0.320575	-1.821713
H	8.573172	-0.355963	-2.878989
H	8.170933	2.013707	-3.757507
H	9.396818	2.622042	-2.626635
H	7.767363	3.329905	-2.624737
H	-3.606909	-0.308328	2.869960
H	-2.781429	-1.691009	3.629875
H	-2.201193	-1.062689	2.066092
H	-1.795268	1.336182	5.663677
H	-2.571896	-0.268620	5.741032
H	-3.356815	1.060340	4.864589
H	-0.853167	2.198849	3.455013
H	-2.483037	1.968268	2.767817
H	-1.064489	1.253667	1.958967
H	-0.045111	-1.803600	7.809737
H	1.298251	-2.307623	8.860969
H	0.937346	-3.216796	7.368415
H	3.390110	-2.929413	6.692742
H	3.658282	-1.992413	8.177765
H	4.105672	-1.298608	6.605173
H	2.678910	0.756206	7.145590
H	2.326891	0.016091	8.730719
H	0.997028	0.566920	7.686561
H	-0.319142	-3.957609	-0.329910
H	-2.081829	-2.874194	-2.580554
H	-2.326061	-4.386648	-1.689878
H	-2.247833	-1.606091	-0.394272
C	-4.226846	-2.369852	-0.812218
H	-2.607005	-3.096011	0.446256
H	-4.400119	-4.306054	-1.685854

H	-5.857623	-3.970591	0.996581
H	-5.619451	-5.477168	0.092331
H	-4.223065	-4.650412	0.811594
H	-6.789649	-4.477771	-1.986981
H	-7.105427	-3.017813	-1.041710
H	-6.270760	-2.892020	-2.607404
C	-4.790347	-1.455131	0.300057
H	-4.312884	-1.821563	-1.767904
C	0.304658	-4.203593	-2.381082
C	-6.219162	-1.004682	0.131484
H	-4.717141	-1.946929	1.277124
H	-4.163288	-0.551173	0.360723
O	-6.395790	-0.328265	-1.037252
O	-7.115439	-1.212067	0.932646
C	-7.776588	0.065354	-1.358555
C	-7.870688	0.104201	-2.873892
H	-8.434501	-0.719711	-0.956903
C	-8.097147	1.392141	-0.684588
H	-8.906415	0.315968	-3.173672
H	-7.228677	0.890808	-3.292155
H	-7.573499	-0.860246	-3.306243
H	-9.126602	1.693609	-0.923993
H	-8.005752	1.301685	0.404834
H	-7.416560	2.181323	-1.035674
H	-0.072224	-5.234621	-2.468991
H	0.185681	-3.698692	-3.352634
H	1.378002	-4.238419	-2.148236
C	-1.712893	0.895422	-1.789225
C	-3.177763	1.232457	-1.836736
C	-3.702009	1.840510	-3.146902
C	-3.967383	0.720681	-4.172451
C	-1.471560	0.003420	-4.149344
C	-2.934657	-0.413272	-4.150034
H	-3.741519	0.315270	-1.599695
H	-2.922105	2.513679	-3.545218
H	-4.955170	0.277650	-3.963601
H	-3.333914	1.893580	-0.977046
H	-4.023488	1.150531	-5.181954
H	-3.098933	-1.077235	-3.288454
H	-3.075455	-1.051522	-5.035785
O	-1.091772	0.993074	-0.715635
O	-0.996183	0.523644	-2.838675
C	-4.954501	2.711818	-2.880686
H	-5.661920	2.076665	-2.319651
C	-4.632996	3.946260	-2.026506
H	-4.201391	3.693068	-1.048861
H	-3.919863	4.602779	-2.549613
H	-5.544872	4.529989	-1.835523
C	-5.632970	3.161534	-4.182265
H	-6.497297	3.803323	-3.959570
H	-4.934405	3.745786	-4.802462
H	-5.995646	2.319847	-4.787118
H	-0.849758	-0.899666	-4.235183
C	-1.069027	1.003588	-5.218109
H	-0.006608	1.252112	-5.118870
H	-1.231507	0.556610	-6.208927
H	-1.654384	1.929125	-5.155225

$II_{CM,axial}$ (in implicit toluene)

C	1.628364	-4.950716	5.138318
C	0.602263	-4.207035	4.542447
C	-0.757121	-4.540449	4.764598
C	-1.067942	-5.662119	5.593996
C	-0.001270	-6.368731	6.157128
C	1.354949	-6.047971	5.957733
C	0.926305	-3.047965	3.642466
N	0.440147	-1.703818	4.114573
C	0.965531	-0.690610	3.146132
C	0.592901	0.741066	3.411903
C	-0.770874	1.126800	3.280560
C	-1.113395	2.498889	3.461492
C	-0.093513	3.388065	3.834258
C	1.247761	3.015541	4.010895
C	1.565509	1.672015	3.771610

O	-1.676618	0.195355	3.009010
Y	-2.189698	-1.708684	3.949754
C	-2.561065	2.977498	3.252241
C	-3.034638	2.589562	1.830586
C	2.345534	4.003004	4.435596
C	1.802004	5.424389	4.662149
O	-1.700805	-3.791352	4.207766
C	-2.530698	-6.050603	5.866153
C	-3.243379	-4.900709	6.616438
C	2.447044	-6.890960	6.633591
C	2.265626	-6.838757	8.167829
O	-1.376201	-0.819081	6.081578
C	-2.244655	-0.026205	6.916097
O	-4.054909	-1.506906	4.858981
C	-5.412368	-1.842945	4.810768
C	-6.403129	-5.635819	2.429286
C	-6.354385	-6.412114	1.106440
C	0.876724	-1.447193	5.502873
C	0.009533	-0.435468	6.233920
C	-3.491550	2.335741	4.306374
C	-2.695093	4.505942	3.383522
C	2.987657	3.519105	5.756019
C	3.432244	4.072686	3.338412
C	-3.260123	-6.331296	4.532533
C	-2.648726	-7.316728	6.734250
C	2.337844	-8.357823	6.158504
C	3.861390	-6.384891	6.302300
C	-5.611030	-3.118807	3.970236
C	-7.055222	-3.408643	3.557666
C	-6.928317	-6.524822	3.562819
H	1.930734	-1.108419	5.543055
H	0.815168	-2.404827	6.037249
H	0.268646	-0.451182	7.306707
H	0.138177	0.588426	5.852276
H	-2.019667	-0.221296	7.976812
H	-3.267480	-0.329809	6.667498
H	-2.103224	1.043861	6.695412

H	0.575687	-0.992108	2.159302
H	2.069625	-0.775758	3.107028
H	-0.360363	4.429863	3.993739
H	2.597217	1.324393	3.875042
H	2.022326	-3.001623	3.491349
H	0.466665	-3.199467	2.650614
H	-0.231142	-7.220377	6.796521
H	2.657456	-4.643818	4.944298
H	-4.522383	2.703527	4.178377
H	-3.166132	2.612801	5.319940
H	-3.504943	1.241769	4.231586
H	-2.065589	5.037375	2.654333
H	-2.429614	4.859164	4.391085
H	-3.740117	4.794927	3.197129
H	-2.373559	3.034264	1.071875
H	-4.054783	2.965505	1.654152
H	-3.030430	1.500353	1.705819
H	2.233636	3.464018	6.555187
H	3.782319	4.210857	6.076932
H	3.434791	2.521055	5.645208
H	3.891902	3.089672	3.162727
H	4.231603	4.771815	3.629700
H	3.000227	4.418036	2.387802
H	1.359289	5.841935	3.746134
H	2.622609	6.089557	4.969251
H	1.039147	5.447386	5.454800
H	-4.310633	-6.595121	4.727890
H	-2.789390	-7.173983	4.003942
H	-3.235267	-5.451012	3.880895
H	-2.200679	-7.178782	7.729730
H	-2.173040	-8.186784	6.257517
H	-3.712511	-7.556316	6.880668
H	-2.749898	-4.706272	7.580706
H	-4.291082	-5.175524	6.818943
H	-3.242898	-3.972582	6.033522
H	1.358689	-8.791043	6.406832
H	3.112006	-8.977751	6.637156

H	2.468034	-8.422893	5.068126
H	4.064095	-6.424676	5.221899
H	4.608152	-7.015989	6.806092
H	4.014155	-5.350103	6.643985
H	2.343178	-5.803350	8.531281
H	3.039474	-7.440458	8.669942
H	1.285042	-7.229809	8.472888
H	-5.967230	-1.025592	4.299019
H	-5.008548	-2.990148	3.057609
H	-5.167485	-3.967040	4.514119
H	-7.559566	-2.449578	3.338555
C	-7.158487	-4.288328	2.297788
H	-7.621409	-3.860855	4.388598
H	-5.363196	-5.371481	2.679926
H	-7.971710	-6.833145	3.391280
H	-6.328510	-7.442961	3.637043
H	-6.882817	-6.018474	4.537163
H	-5.646403	-7.250412	1.176995
H	-7.333699	-6.838611	0.841748
H	-6.040398	-5.767617	0.272215
C	-8.637562	-4.445650	1.878133
H	-6.662220	-3.733168	1.483248
C	-5.981233	-1.991634	6.225390
C	-8.808360	-4.696265	0.390792
H	-9.142006	-5.186631	2.507467
H	-9.153624	-3.487437	2.053128
O	-9.588189	-5.708721	-0.067527
O	-8.271958	-3.981957	-0.440040
C	-10.271067	-6.666816	0.807818
C	-10.443955	-7.922989	-0.030984
H	-9.615064	-6.893065	1.663268
C	-11.590117	-6.074099	1.283254
H	-10.937894	-8.704138	0.563030
H	-11.061756	-7.711723	-0.915075
H	-9.469467	-8.301566	-0.366726
H	-12.123053	-6.797763	1.915793
H	-11.430722	-5.158150	1.868012
H	-12.224897	-5.830017	0.419712
H	-7.055781	-2.232343	6.209964
H	-5.446719	-2.795399	6.753412
H	-5.845358	-1.057380	6.788773
C	-3.766517	-1.594769	1.032094
C	-3.784676	-1.769767	-0.455481
C	-5.041128	-2.536882	-0.960590
C	-6.222972	-1.571840	-1.115428
C	-5.668248	-0.024791	0.911371
C	-6.678242	-0.908598	0.187392
H	-3.746696	-0.782410	-0.943237
H	-5.323382	-3.292412	-0.204866
H	-5.957124	-0.795792	-1.855133
H	-2.861391	-2.299980	-0.708701
H	-7.085149	-2.119489	-1.511369
H	-7.553206	-0.276005	-0.030144
H	-7.027180	-1.677487	0.896212
O	-2.940580	-2.186563	1.744407
O	-4.664132	-0.832573	1.643704
C	-4.687061	-3.314056	-2.258282
H	-4.151625	-2.614070	-2.926956
C	-3.758212	-4.498439	-1.947820
H	-2.856305	-4.205505	-1.392633
H	-4.290712	-5.248872	-1.342511
H	-3.429923	-4.989875	-2.874712
C	-5.930188	-3.829486	-2.998945
H	-5.628298	-4.485288	-3.828453
H	-6.578921	-4.406113	-2.322135
H	-6.532488	-3.015564	-3.423446
H	-5.109866	0.612517	0.206128
C	-6.311061	0.827766	1.990001
H	-6.927631	0.204090	2.652350
H	-5.545943	1.326732	2.594766
H	-6.951657	1.589831	1.526385

Literature

[1] H. Eyring, *J. Chem. Phys.* **1935**, *3*, 107-115.

[2] D. Zhang, M. A. Hillmyer, W. B. Tolman, *Biomacromolecules* **2005**, *6*, 2091-2095.

[3] C. L. Wanamaker, L. E. O'Leary, N. A. Lynd, M. A. Hillmyer, W. B. Tolman, *Biomacromolecules* **2007**, *8*, 3634-3640.

[4] J. Shin, M. T. Martello, M. Shrestha, J. E. Wissinger, W. B. Tolman, M. A. Hillmyer, *Macromolecules* **2010**, *44*, 87-94.

[5] J. A. Wilson, S. A. Hopkins, P. M. Wright, A. P. Dove, *Biomacromolecules* **2015**, *16*, 3191-3200.

[6] F. Adams, T. M. Pehl, M. Kränzlein, S. A. Kernbichl, J.-J. Kang, C. M. Papadakis, B. Rieger, *Polym. Chem.* **2020**, *11*, 4426-4437.

[7] N. Zhao, X.-X. Cao, J.-F. Shi, Z.-B. Li, *Chin. J. Polym. Sci.* **2020**, *38*, 1092-1098.

[8] J. R. Lowe, M. T. Martello, W. B. Tolman, M. A. Hillmyer, *Polym. Chem.* **2011**, *2*, 702-708.

[9] J. Yang, S. Lee, W. J. Choi, H. Seo, P. Kim, G.-J. Kim, Y.-W. Kim, J. Shin, *Biomacromolecules* **2015**, *16*, 246-256.

[10] C.-X. Cai, L. Toupet, C. W. Lehmann, J.-F. Carpentier, *J. Organomet. Chem.* **2003**, *683*, 131-136.

[11] K. C. Hultzsch, P. Voth, K. Beckerle, T. P. Spaniol, J. Okuda, *Organometallics* **2000**, *19*, 228-243.

[12] E. Y. Tshuva, S. Groysman, I. Goldberg, M. Kol, Z. Goldschmidt, *Organometallics* **2002**, *21*, 662-670.

[13] TURBOMOLE V7.4.1 2020, a development of University of Karlsruhe and Forschungszentrum Karlsruhe GmbH, 1989-2007, TURBOMOLE GmbH, since 2007; available from https://www.turbomole.org.

[14] a) A. D. Becke, *Physical Review A* **1988**, *38*, 3098-3100; b) J. P. Perdew, *Physical Review B* **1986**, *33*, 8822-8824.

[15] S. Grimme, *Journal of Computational Chemistry* **2004**, *25*, 1463-1473.

[16] F. Weigend, R. Ahlrichs, *Physical Chemistry Chemical Physics* **2005**, *7*, 3297-3305.

[17] A. Klamt, G. Schüürmann, *J. Chem. Soc., Perkin Trans.* **1993**, 799-805.

[18] K. Eichkorn, O. Treutler, H. Öhm, M. Häser, R. Ahlrichs, *Chem. Phys. Lett.* **1995**, *240*, 283-290.

[19] M. Sierka, A. Hogekamp, R. Ahlrichs, *J. Chem. Phys.* **2003**, *118*, 9136-9148.

[20] a) A. D. Becke, *J. Chem. Phys.* **1993**, *98*, 5648-5652; b) C. Lee, W. Yang, R. G. Parr, *Physical Review B* **1988**, *37*, 785-789; c) S. H. Vosko, L. Wilk, M. Nusair, *Canadian Journal of physics* **1980**, *58*, 1200-1211.

[21] S. Kozuch, S. Shaik, *Acc. Chem. Res.* **2011**, *44*, 101-110.
